# Anti-Parkinsonian 4-hydroxy-2-pyridones from an endolichenic fungus*, Tolypocladium* sp. (strain CNC14)

**DOI:** 10.1093/jimb/kuaf027

**Published:** 2025-09-03

**Authors:** Jin Won Choi, Chaesun Kwon, Jin Woo Lee, Jae-Seoun Hur, Min-Kyoo Shin, Sang Hee Shim

**Affiliations:** Natural Products Research Institute, College of Pharmacy, Seoul National University, Seoul 08826, Republic of Korea; College of Pharmacy and Research Institute of Pharmaceutical Sciences, Seoul National University, Seoul 08826, Republic of Korea; College of Pharmacy, Duksung Women's University, Seoul 01369, Republic of Korea; Korean Lichen Research Institute, Sunchon National University, Suncheon 57922, Republic of Korea; Natural Products Research Institute, College of Pharmacy, Seoul National University, Seoul 08826, Republic of Korea; College of Pharmacy and Research Institute of Pharmaceutical Sciences, Seoul National University, Seoul 08826, Republic of Korea; Natural Products Research Institute, College of Pharmacy, Seoul National University, Seoul 08826, Republic of Korea

**Keywords:** *Tolypocladium* sp, Endolichenic fungus, 4-Hydroxy-2-pyridone alkaloids, Parkinson's disease

## Abstract

4-Hydroxy-2-pyridone alkaloids have attracted considerable attention because of their intriguing structures and diverse bioactivities. In our previous study, 4-hydroxy-2-pyridone alkaloids were shown to exhibit potent activity against neuron-associated targets. To discover this class of neuroactive compounds, an array of endolichenic fungal extracts was screened by analyzing liquid chromatography-ultraviolet-mass spectrometry (LC-UV-MS) profiles. The screening yielded strain *Tolypocladium* sp. (strain CNC14), which produced compounds with characteristic Ultraviolet patterns for 4-hydroxy-2-pyridone alkaloids using our in-house library. Based on these findings, we conducted a chemical investigation, which led to the isolation of four new (**1**–**4**) and ten known (**5**–**14**) compounds. Their structures were elucidated via spectroscopic methods such as nuclear magnetic resonance and mass spectrometry. The stereochemistry of the new compounds (**1**–**4**) was established using rotating frame overhauser effect spectroscopy (ROESY), and the electronic circular dichrosim (ECD) was compared with the calculated data. Interestingly, the side chains of 4-hydroxy-2-pyridone in **1** and **2** were cyclized in different directions to form benzopyrano[3,4-b]pyridinol from previously reported compounds, and all the new compounds were predicted to be biosynthesized from reduced tolypyridone C (**7**) via the hetero-Diels–Alder reaction. Among the isolated compounds, **4** significantly protected neuronal cells against treatment with 1-methyl-4-phenylpyridinium (MPP^+^), a Parkinsonian neurotoxin, in an *in vitro* Parkinson's disease model.

**One-Sentence Summary**: Four new neuroprotective 4-hydroxy-2-pyridone alkaloids were discovered from an endolichenic fungus *Tolypocladium* sp.

## Graphical Abstract

**Graphical Abstract:**
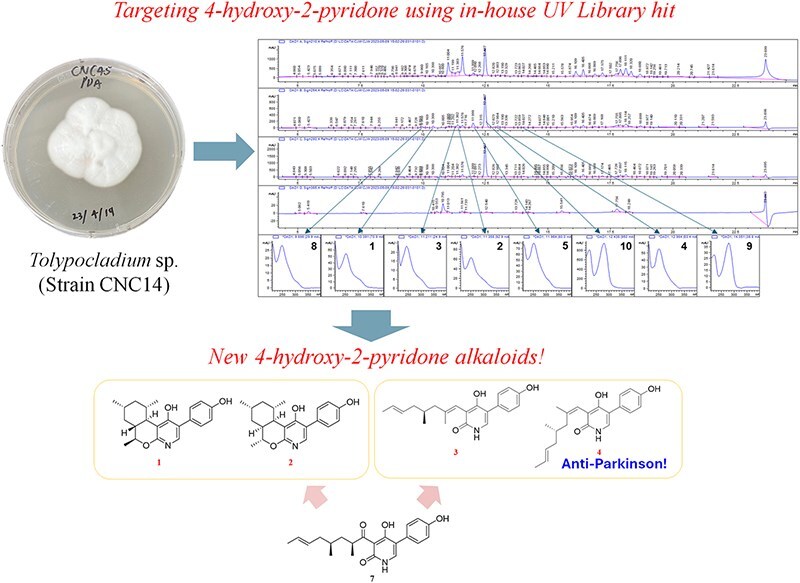

## Introduction

As 4-hydroxy-2-pyridone alkaloids are natural products that are extremely rich in diversity and biological activity, they are still being researched in these days. Ricinine, an acyl-chained pyridine alkaloid, was reported as an insecticidal and hepatotoxic natural compound, produced by a plant *Ricinus communis* and its endophytic fungi (Tamir & Ginsburg, 1959). Tenellin, another 4-hydroxy-3-acyl modified 2-pyridone, inhibits ATPase in membranes (McInnes et al., 1974). Furthermore, the biosynthetic pathway of 4-hydroxy-2-pyridone was unveiled through gene knockout, antisense RNA, and gene co-expression. They are generated by an enzymatic ring expansion from five-membered tetramate ring to six-membered pyridone (Laura et al., 2008). Referring to our recent report, new anti-Parkinson tolypyridone derivatives, tolypyridone I and J, derivatives of tolypyridone A, were discovered from a deep-sea marine fungus, *Tolypocladium cylindrosporum* (Jung et al., 2023). In another chemical investigation, sambutoxin and fischerin, 4-hydroxy-2-pyridone alkaloids with potent neuroprotective activities, were isolated from extracts of *Fusarium* sp. and *Neosartorya fischerii*, respectively (Bang et al., 2019). As demonstrated in the above-mentioned studies, 4-hydroxy-2-pyridones have intriguing structures and show neuron-associated activities.

Parkinson's disease (PD) is a prevalent neurodegenerative disease characterized by motor dysfunctions, such as tremors, bradykinesia, and gait rigidity. These symptoms arise from the progressive degeneration of dopaminergic neurons in the substantia nigra pars compacta, which results in the depletion of dopamine in the striatum (Lotankar et al., 2017). The primary therapeutic intervention for PD is L-3,4-dihydroxyphenylalanine (L-DOPA), which is a precursor of dopamine. However, L-DOPA administration is associated with limitations, including the lack of sustained beneficial effects and the occurrence of side effects, such as agitation, delusions, and hallucinations. Preserving dopaminergic neurons from degeneration represents a pivotal focus for PD drug development. Earlier investigations have demonstrated the neuroprotective potential of natural products, such as DA-9805 and WIN-1001X, which protect dopaminergic neurons in *in vitro* and *in vivo* PD models (Eo et al., 2019; Li et al., 2021). MT101, a natural product derived from herbs, improves motor function in animal models of PD by preventing the loss of dopaminergic neurons (Kim et al., 2022). These findings highlight the potential of natural products as candidates for developing novel therapeutics to mitigate dopaminergic neuronal loss in PD.

Lichen is among the most fascinating symbiotic biological organisms as a source of bioactive natural products and produces various structural compounds (Furmanek et al., 2022). Endolichenic fungi are also known to produce a large number of secondary metabolites with unique structures, varied skeletons, and fascinating bioactivities that may lead to the discovery of pharmacological lead compounds because of their mutualistic symbiotic relationship with hosts (Zhang et al., 2024). In an effort to discover new bioactive 4-hydroxy-2-pyridone alkaloids from natural sources, we conducted LC-UV-MS profiling of extracts of endolichenic fungi, collected from the Korean peninsula, and compared them with those in our in-house library of fungal metabolites. Among these, the fungus *Tolypocladium* sp. (strain CNC14) extract isolated from a lichen *Parmotrema tinctorum* produced several compounds with UV patterns characteristic of 4-hydroxy-2-pyridone alkaloids, such as tolypyridones and sambutoxin, but with different molecular weights. Thus, we predicted that *Tolypocladium* sp. (strain CNC14) could produce new secondary metabolites with a 4-hydroxy-2-pyridone moiety, which prompted us to cultivate this strain on a large scale in the laboratory for chemical investigations. Four new (**1**–**4**) and ten known (**5**–**14**) 4-hydroxy-2-pyridone type compounds were isolated from these cultures. Here, we describe the structure and bioactivity of these isolated metabolites.

## Materials and Methods

### General Experimental Procedures

Optical rotation was recorded on a JASCO P-2000 polarimeter (JASCO, Easton, PA, USA). High-resolution electrospray ionization mass spectrometry (HRESIMS) data were acquired with a Q-TOF mass spectrometer on a Q-TOF 6530 MS/1290 Infinity system (Agilent Technologies, Santa Clara, CA, USA). Nuclear magnetic resonance (NMR) spectra were obtained using Bruker 600 MHz (^1^H: 600 MHz, ^13^C: 150 MHz) (Bruker, Billerica, MA, USA) and Bruker 800 MHz (^1^H: 800 MHz, ^13^C: 200 MHz) (Bruker, Billerica, MA, USA) spectrometers with CD_3_OD (Cambridge Isotope Laboratories, Inc., Tewksbury, MA, USA), DMSO-*d*_6_ (Cambridge Isotope Laboratories, Inc., Tewksbury, MA, USA).

### Fungal Materials

The fungal strain *Tolypocladium* sp. (strain CNC14) (GenBank accession number: SUB15468886  CNC14_P20_ITS45  PV945089) was obtained from the Korean Lichen & Allied Bioresources Center (KoLABIC) at Sunchon National University, Korea. It was originally isolated from the lichen *P. tinctorum*. The strain was cultured on potato dextrose agar medium (24.0-g potato dextrose broth, 18.0-g agar per 1 L sterilized distilled water) for fourteen days at 29°C. Agar plugs were cut into small pieces (0.5 × 0.5 cm) under aseptic conditions, inoculated into 20 flasks of 2.8 L containing 500 ml of the same medium.

### Fermentation, Extraction, and Isolation

Culture media were extracted with ethyl acetate three times. The solvent was evaporated under reduced pressure at 33°C to yield the extract (4.0 g). The extract was fractionated by medium-pressure column chromatography over silica by a stepwise gradient of *n*-hexane-dichloromethane-MeOH (from 100:0:0, 50:50:0, 0:100:0, 0:99:1, 0:98:2, 0:96:4, 0:93:7, 0:90:10, 0:85:15, 0:75:25, 0:50:50, 0:25:75, to 0:0:100) to obtain 10 fractions (fraction 1–10). Fraction 4 (350.0 mg) was separated by reversed-phase high performance liquid chromatography (HPLC) (Phenomenex, Luna C_18_ [2], 5 µ, 250 × 10.0 mm, isocratic aqueous 60 % ACN, 2.0 ml/min, UV 210, 254, 280, and 365 nm) to obtain **1** (2.0 mg), **5** (10.0 mg), **6** (1.5 mg), **7** (1.0 mg), **9** (8.0 mg), and **10** (6.0 mg). To isolate **2** (0.8 mg), **3** (6.3 mg), **4** (6.5 mg), **13** (42.0 mg), and **14** (4.3 mg), fraction 5 (186.0 mg) was purified by reversed-phase HPLC (Phenomenex, Luna C_18_, 5 µ, 250 × 10.0 mm, isocratic aqueous 65 % ACN, 2.0 ml/min, UV 210, 254, 280, and 365 nm). Fraction 8 (470.0 mg) was separated by reversed-phase HPLC (Phenomenex, Luna C_18_, 5 µ, 250 × 10.0 mm, isocratic aqueous 75 % ACN, 2.0 ml/min, UV 210,254, 280, and 365 nm) to obtain **8** (2.0 mg), **11** (170.0 mg), and **12** (30.0 mg).

Tolypyridinol A (**1**): white powder; (α)_D_^20^ =–234.85 (*c* 0.1 MeOH); UV (MeOH) λ_max_ (log *ε*) 209 (2.09), 241 (2.06), 272 (1.72) nm; ECD (MeOH) *λ_max_* (Δ*ε*) 209 (−12.38), 241 (−21.77), 272 (−0.79); for ^1^H and ^13^C NMR data (in CD_3_OD), see Table 1; ^1^H-^1^H COSY, H-7 ↔ H-8, H-7 ↔ H-12, H-8 ↔ H_3_-16, H-8 ↔ H-9a, H-8 ↔ H-9b, H-9a ↔ H-10, H-9b ↔ H-10, H-10 ↔ H_3_-15, H-10 ↔ H-11a, H-10 ↔ H-11b, H-11a ↔ H-12, H-11b ↔ H-12, H-12↔ H-13, H-13↔ H_3_-14, H-2'↔ H-3', H-5'↔ H-6'; total heteronuclear multiple bond correlations (HMBC) correlations H-# → C-#, H-6 → C-2, C-4, C-5, and C-1', H-7 → C-2, C-3, C-4, C-8, and C-12, H-8 → C-7, C-9, C-12 and C-16, H_2_-9 → C-8, C-10, C-15, and C-16, H-10 → C-9, C-11, C-12, and C-15, H_2_-11 → C-10, C-12, C-13, and C-15, H-12 → C-7, C-11, C-13, and C-14, H_3_-14 → C-12, and C-13, H_3_-15 → C-9, C-10, and C-11, H_3_-16 → C-7, C-8, and C-9, H-2' → C-5, C-3', and C-4', H-3' → C-1', C-2', C-4', and C-5', H-5' → C-1', C-2', C-3', and C-4', H-6' → C-5, C-4', and C-5'.; (+)HRESIMS *m/z* 340.1913 (M + H)^+^ (calculated for C_21_H_25_NO_3_,340.1907).

**Table 1.** ^1^H and ^13^C NMR Data of 1–4 in CD_3_OD^a^

No.	1		2		3		4	
	*δ*_C_	*δ*_H_, Multi (*J* in Hz)	*δ*_C_	*δ*_H_, Multi (*J* in Hz)	*δ*_C_	*δ*_H_, Multi (*J* in Hz)	*δ*_C_	*δ*_H_, Multi (*J* in Hz)
1	–	–	–	–	–	–	–	–
2	159.0 (C)	–	155.9 (C)	–	165.2 (C)	–	165.0 (C)	–
3	111.1 (C)	–	110.7 (C)	–	111.6 (C)	–	111.3 (C)	–
4	175.2 (C)	–	170.5 (C)	–	164.3 (C)	–	164.3 (C)	–
5	126.2 (C)	–	126.5 (C)	–	117.7 (C)	–	117.7 (C)	–
6	134.1 (CH)	7.55 s	131.4 (CH)	7.31 s	132.7 (CH)	7.20 s	132.7 (CH)	7.20 s
7	44.9 (CH)	2.47 t (9.9)	38.1 (CH)	2.73 dd (11, 3.8)	117.0 (CH)	5.77 s	117.2 (CH)	5.82 s
8	42.0 (CH)	1.71 m	37.6 (CH)	1.60 m	114.4 (C)	–	145.1 (C)	–
9	46.9 (CH_2_)	1.81 brd (13.1) 1.08 dt (13.1, 11.8)	45.8 (CH_2_)	1.74 brd (13.3) 0.92 overlapped	48.3 (CH_2_)	2.25 dd (13, 5.9) 2.00 dd (13, 8.3)	46.9 (CH_2_)	2.02 dd (14, 5.6) 1.77 dd (14, 7.7)
10	34.15 (CH)	1.71 m	28.2 (CH)	1.67 m	32.8 (CH)	1.75 m	32.8 (CH)	1.37 m
11	38.1 (CH_2_)	1.90 brd (12.3 0.92 dt (12.3, 11.9)	37.2 (CH_2_)	1.89 brd (13.9 1.37 ddd (13.9, 13.5, 49.9)	41.0 (CH_2_)	2.10 m 1.85 m	41.3 (CH_2_)	1.90 m 1.68 m
12	50.6 (CH)	1.63 m	41.0 (CH)	1.74 overlapped	131.5 (CH)	5.46 m	131.3 (CH)	5.31 m
13	82.2 (CH)	4.01 m	76.2 (CH)	4.79 m	127.3 (CH)	5.46 m	127.3 (CH)	5.32 m
14	19.2 (CH_3_)	1.43 d (4.7)	19.2 (CH_3_)	1.43 d (6.2)	18.3 (CH_3_)	1.64 d (4.1)	18.3 (CH_3_)	1.58 d (5.1)
15	22.8 (CH_3_)	0.85 d (6.5)	23.3 (CH_3_)	0.92 d (6.4)	20.1 (CH_3_)	0.96 d (6.5)	20.3 (CH_3_)	0.76 d (6.3)
16	23.4 (CH_3_)	1.16 d (6.8)	21.3 (CH_3_)	0.91 d (6.7)	23.9 (CH_3_)	1.59 s	18.9 (CH_3_)	1.90 d (1.4)
1'	125.9 (C)	–	128.5 (C)	–	127.2 (C)	–	126.8 (C)	–
2'/6'	131.8 (CH)	7.42 (brd, 8.4)	131.4 (CH)	7.31 (brd, 8.7)	131.5 (CH)	7.24 (brd, 8.4)	131.7 (CH)	7.26 (brd, 8.7)
3'/5'	116.8 (CH)	6.84 (brd, 8.4)	116.1 (C)	6.79 (brd, 8.7)	116.2 (CH)	6.81 (brd, 8.4)	116.4 (CH)	6.80 (brd, 8.7)
4'	159.7 (C)	–	157.9 (C)	–	158.3 (C)	–	158.1 (C)	–

^a1^H and ^13^C NMR were measured at 800 and 200 MHz, respectively.

Tolypyridinol B (**2**): white powder; (α)_D_^20^ =–77.90 (*c* 0.02 MeOH); UV (MeOH) λ_max_ (log *ε*) 209 (1.94), 242 (1.93), 272 (1.53) nm; ECD (MeOH) *λ_max_* (Δ*ε*) 209 (−3.59), 241 (−8.16), 272 (+0.52); for ^1^H and ^13^C NMR data (in CD_3_OD), see Table 1; ^1^H-^1^H COSY, H-7 ↔ H-8, H-7 ↔ H-12, H-8 ↔ H_3_-16, H-8 ↔ H_2_-9a, H-8 ↔ H_2_-9b, H-9a ↔ H-10, H_2_-9b ↔ H-10, H-10 ↔ H_3_-15, H-10 ↔ H_2_-11a, H-10 ↔ H_2_-11b, H_2_-11a ↔ H-12, H_2_-11b ↔ H-12, H-12↔ H-13, H-13↔ H_3_-14, H-2'↔ H-3', H-5'↔ H-6'; total HMBC correlations H-# → C-#, H-6 → C-2, C-4, C-5, and C-1', H-7 → C-2, C-3, C-4, C-8, and C-12, H-8 → C-7, C-9, C-12, and C-16, H_2_-9 → C-8, C-10, C-15, and C-16, H-10 → C-9, C-11, C-12, and C-15, H_2_-11 → C-10, C-12, C-13, and C-15, H-12 → C-7, C-11, C-13, and C-14, H_3_-14 → C-12, and C-13, H_3_-15 → C-9, C-10, and C-11, H_3_-16 → C-7, C-8, and C-9, H-2' → C-5, C-3', and C-4', H-3' → C-1', C-2', C-4', and C-5', H-5' → C-1', C-2', C-3', and C-4', H-6' → C-5, C-4', and C-5'.; (+)HRESIMS *m/z* 340.1910 [M + H]^+^ (calculated for C_21_H_25_NO_3_,340.1907).

Tolypyridone K (**3**): colorless gel; [α]_D_^20^ = + 66.40 (*c* 0.1 MeOH); UV (MeOH) λ_max_ (log *ε*) 209 (2.40), 254 (2.31); ^1^H NMR and ^13^C NMR data, see Table 1; ^1^H-^1^H COSY, H_2_-9a ↔ H-10, H_2_-9b ↔ H-10, H-10 ↔ H_3_-15, H-10 ↔ H-11, H-11 ↔ H-12, H-12 ↔ H-13, H-13 ↔ H_3_-14, H-2'↔ H-3', H-5'↔ H-6'; total HMBC correlations H-# → C-#, H-6 → C-2, C-4, C-5, and C-1', H-7 → C-2, C-3, C-4, C-8, C-9, and C-16, H_2_-9 → C-8, C-10, C-15, and C-16, H-10 → C-8, C-9, C-11, C-12, and C-15, H_2_-11 → C-10, C-12, C-13, and C-15, H-12 → C-10, C-11, C-13, and C-14, H-13 → C-11, C-12, and C-14, H_3_-14 → C-12, and C-13; (+)HRESIMS *m/z* 340.1913 [M + H]^+^ (Calculated for C_21_H_25_NO_3_,340.1907).

Tolypyridone L (**4**): colorless gel; [α]_D_^20^ = + 19.40 (*c* 0.05 MeOH); UV (MeOH) λ_max_ (log *ε*) 207 (2.26), 254 (2.19) nm; for ^1^H and ^13^C NMR data (in CD_3_OD), see Table 1; ^1^H-^1^H COSY, H_2_-9a ↔ H-10, H_2_-9b ↔ H-10, H-10 ↔ H_3_-15, H-10 ↔ H-11, H-11 ↔ H-12, H-12 ↔ H-13, H-13 ↔ H_3_-14, H-2'↔ H-3', H-5'↔ H-6'; total HMBC correlations H-# → C-#, H-6 → C-2, C-4, C-5, and C-1', H-7 → C-2, C-3, C-4, C-8, C-9, and C-16, H_2_-9 → C-8, C-10, C-15, and C-16, H-10 → C-8, C-9, C-11, C-12, and C-15, H_2_-11 → C-10, C-12, C-13, and C-15, H-12 → C-10, C-11, C-13, and C-14, H-13 → C-11, C-12, and C-14, H_3_-14 → C-12, and C-13; (+)HRESIMS *m/z* 340.1912 [M + H]^+^ (calculated for C_21_H_25_NO_3_,340.1907).

### DP4 + Calculations

Models for compound **3** were constructed using the Chem3D software, and energy minimization was performed using the molecular mechanics force field (MMFF) approach. Conformers within a 10-kJ/mol energy threshold were further refined using density functional theory (DFT) at the B3LYP/6–31 G (d, p) level. The shielding tensors for the optimized conformers were averaged according to Boltzmann weighting. Subsequent DP4 + analysis was conducted on these averaged tensors using a spreadsheet provided in the original study. Chemical shifts were calculated from the previously reported GIAO magnetic shielding tensor values (Grimblat et al., 2015).

### ECD Calculations of Compounds 1 and 2

Computation DFT calculations were utilized to find the lowest energy conformation of compounds **1** and **2**. The energy-minimized structures of compounds were carried out using Avogadro 1.2.0 with the MMFF force field. Then, the ground-state geometry optimizations were conducted via Tmolex 3.4 by using DFT. The calculated ECD data corresponding to the optimized structures were obtained with TDDFT with the def2-SV(P) basis set for all atoms at the DFT level (functional B3-LYP/gridsize m3). The calculated ECD spectra were simulated by overlapping each transition, where σ is the width of the band at 1/e height. The observed ECD spectrum of **1** and **2** showed negative Cotton effects at 209 and 241 nm.

### Cell Culture

SH-SY5Y cells were cultured in Dulbecco's Modified Eagle Medium (Welgene, cat # LM 001-05, South Korea), which contained 1% antibiotic-antimycotic solution (Welgene, cat # LS 203-01, South Korea) and 10% FBS (Gibco, cat # 16000-044, USA). The cells were kept at 37°C with 5% CO_2_.

### Cell Viability Assay

Cell viability was measured as described previously (Jung et al., 2023). Briefly, SH-SY5Y cells were seeded in 96-well plates at 3 × 10^4^ cells/well density. Cells were pretreated with compounds for 24 hr, followed by incubation with 2 mmol/L MPP^+^ for 24 hr. Ten microliters of MTT solution (Sigma-Aldrich, cat # M2128, USA) were added to serum-free culture media for 3.5 hr at 37°C. To dissolve the purple formazan, dimethylsulfoxide was added. A plate reader (Molecular Devices, cat # M5e, USA) was used to measure optical density at 570 nm.

### Reverse Transcription Quantitative Polymerase Chain Reaction

Total RNA was extracted from SH-SY5Y cells using TRIzol (Molecular Research Center). To eliminate genomic DNA contamination, 1 µg of RNA was treated with DNase I (Enzynomics) prior to cDNA synthesis. cDNA was synthesized from 1 µg of DNase-treated RNA using MMLV Reverse Transcriptase (Enzynomics) and Random Hexamers (Enzynomics). Quantitative polymerase chain reaction (PCR) was conducted on a QuantStudio 5 Real-Time PCR System using a real-time PCR master mix containing SYBR Green (BIOFACT). The following primer sequences were used: GAPDH (forward: 5′-GTCTCCTCTGACTTCAACAGCG-3', reverse: 5′-ACCACCCTGTTGCTG TAGCCAA-3′), IL-1β (forward: 5'-TTGTTGAGCCAGGCCTCTCT-3', reverse: 5'-ACCAAA TGTGGCCGTGGTT-3'), and TNF-α (forward: 5'-CCTCTCTCTAATCAGCCCTCTG-3', reverse: 5'-GAGGACCTGGGAGTAGATGAG-3'). Relative gene expression levels were calculated using the 2^−ΔΔCt method, with GAPDH as the internal normalization control. All experiments were performed in three independent biological replicates.

### Statistical Analysis

All statistical analyses were conducted using GraphPad Prism, version 10.1.1 (GraphPad Software, Inc). One-way analysis of variance (ANOVA) with Tukey's post hoc analysis was used for statistical differences. Values are presented as mean ± SEM.

## Results and Discussion

### Structure Elucidation of the Isolated Compounds

During the LC-UV-MS profiling of endolichenic fungal extracts, *Tolypocladium* sp. (strain CNC14) produced compounds with a UV spectrum that was highly similar to those of sambutoxtin and tolypyridone A (Choi & Shim, 2017; Jung et al., 2023). Thus, several compounds with UV and different molecular weights were targeted by this strain (Fig. S1).

**Fig. 1.**
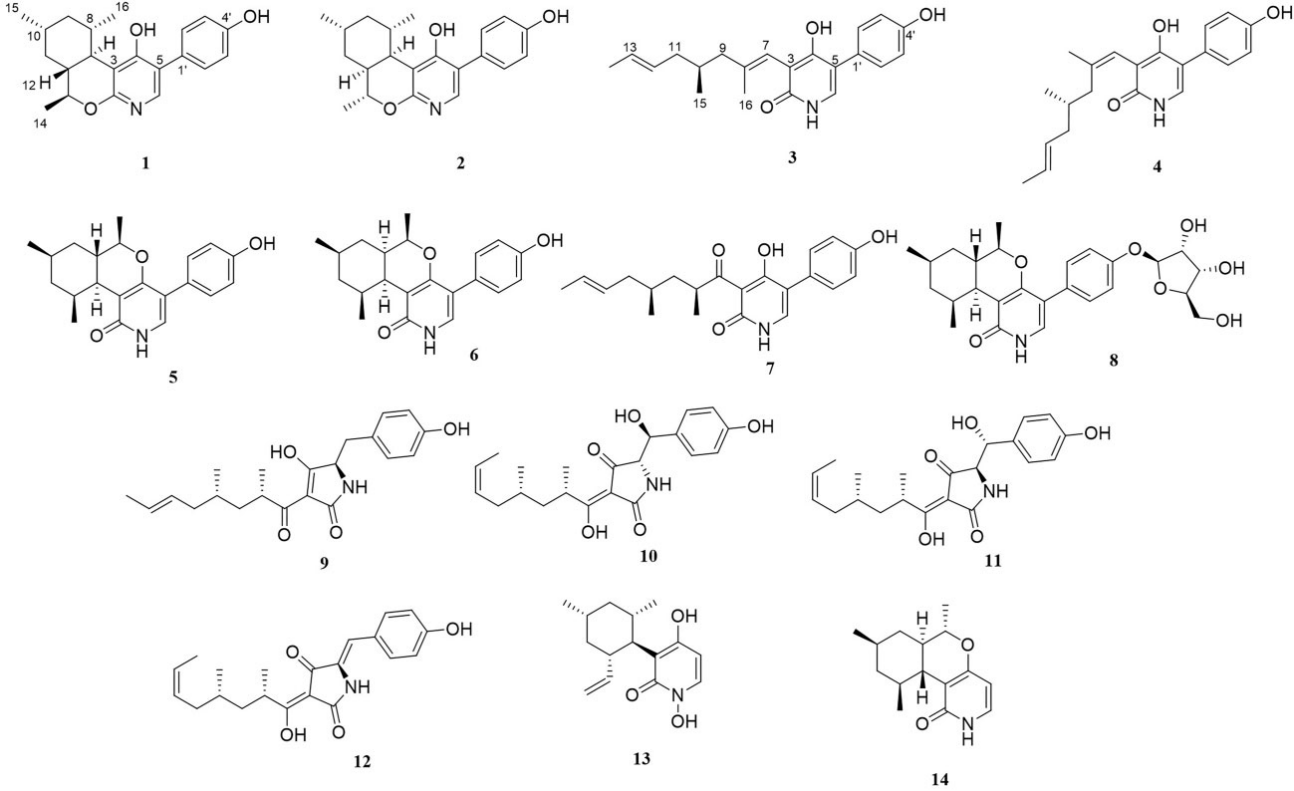
Structures of compounds isolated from the culture extract of *Tolypocladium* sp. (strain CNC14).

**Fig. 2.**
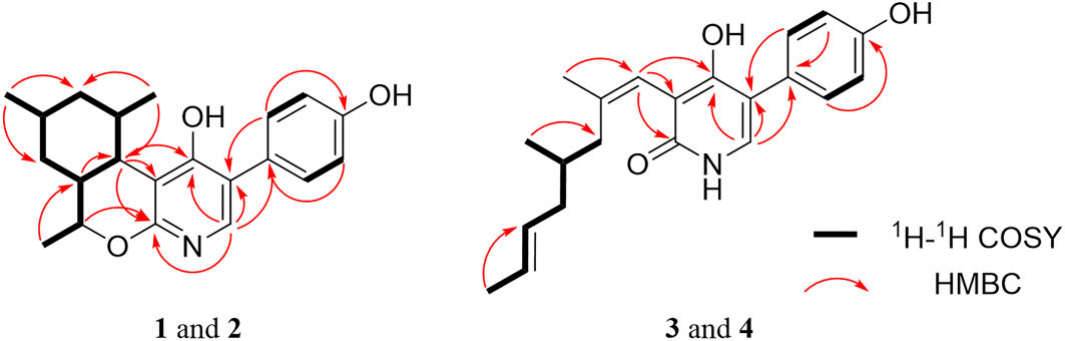
Key ^1^H-^1^H COSY (bold lines) and HMBC (arrows) correlations for compounds **1**–**4**.

Compound **1** was isolated as a white powder (Fig. 1). Its molecular formula was established as C_21_H_25_NO_3_ (10 unsaturations) using (+)HRESIMS (observed at *m/z* 339.1913 [M + H]^+^, calculated for C_21_H_25_NO_3_,339.1907). Analysis of ^1^H, ^13^C, and HSQC NMR data for **1** revealed that it has six double bonds, one sp^3^ oxymethine group, four non-oxygenated sp^3^ methines, two non-oxygenated methylenes, and three methyl groups (Table 1). As ^1^H and ^13^C NMR data were very similar to those of tolypyridone A (**5**) (Li et al., 2015), **1** was predicted to have a similar moiety as well. The *J* values of the aromatic protons (8.4 Hz) and their characteristic symmetric patterns indicated A_2_B_2_ system of benzene. The complete structure was established by analyzing 1D and 2D NMR data. Strong HMBC of the proton of pyridone moiety (H-6) at *δ*_H_ 7.55 and aromatic methine H-2', 6' (*δ*_H_ 7.42) with sp^2^ carbons, C-1' (*δ*_C_ 110.4) and C-5 (*δ*_C_ 126.2), supported the attachment of the *para*-hydroxy phenyl group at C-5 of the pyridone. Interpretation of ^1^H-^1^H COSY spectrum provided a partial structure (I), CH(CH)-CH(CH_3_)–CH_2_–CH(CH_3_)–CH_2_–CH(CH)–CH_3_ corresponding to C7(C12)–C8(C16)–C9–C10(C15)–C11–C12(C7)–C14, which indicated the presence of dimethyl-cyclohexane linked to an ethyl group (Fig. 2). The HMBC of the methine H-7 (*δ*_H_ 2.47) with C-2 (*δ*_C_ 159.0), C-3 (*δ*_C_ 111,1), and C-4 (*δ*_C_ 175.2) of the pyridone connected C-7 of the partial structure I, ethyl-dimethyl-cyclohexane with C-3 of the pyridone (Fig. 2). In addition, the methyl-bearing methine carbon C-13 was linked to C-2 of the pyridone through oxygen, forming a pyran ring by HMBC of the oxymethine H-13 (*δ*_H_ 4.01) with C-2 and C-4 the pyridone ring (Fig. 2). Thus, the planar structure of **1** was established, as shown in the Fig. 1.

**Fig. 3.**
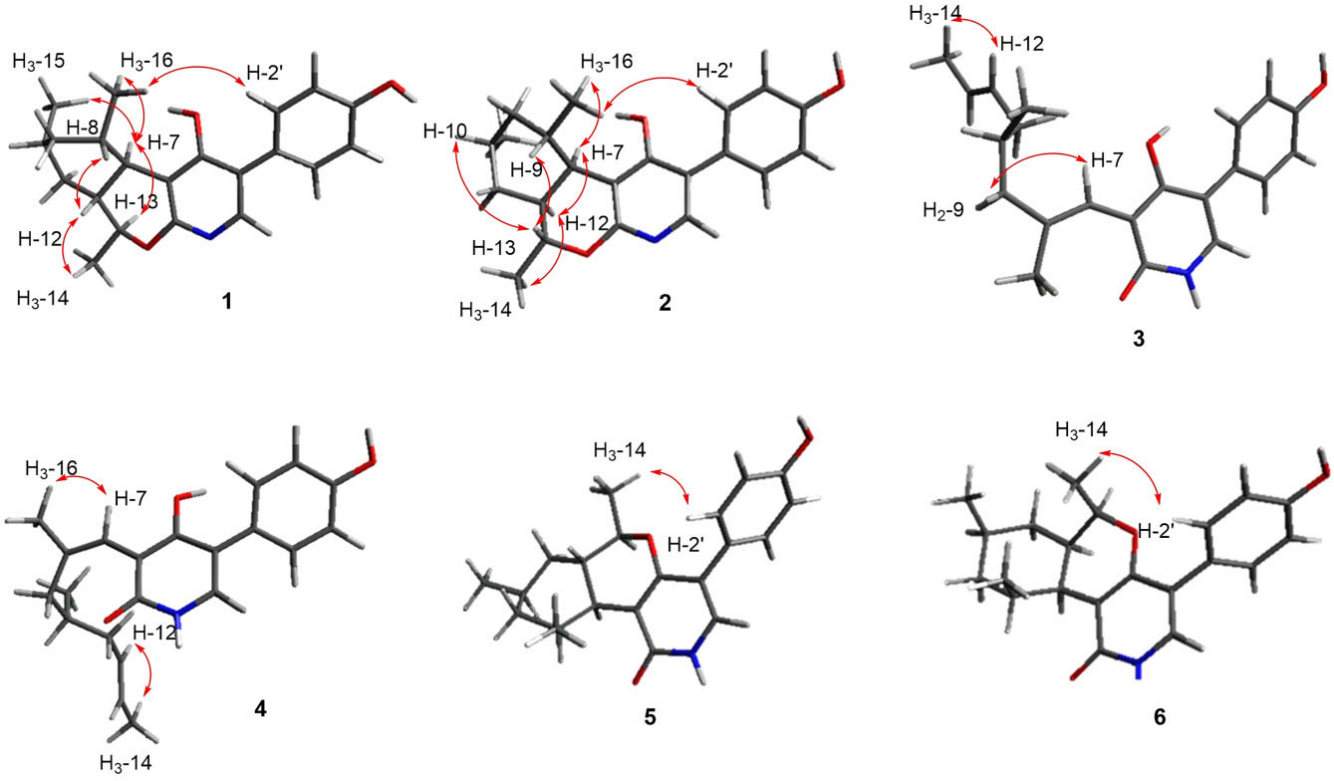
Key ROESY correlations for compounds **1**–**6**.

**Fig. 4.**
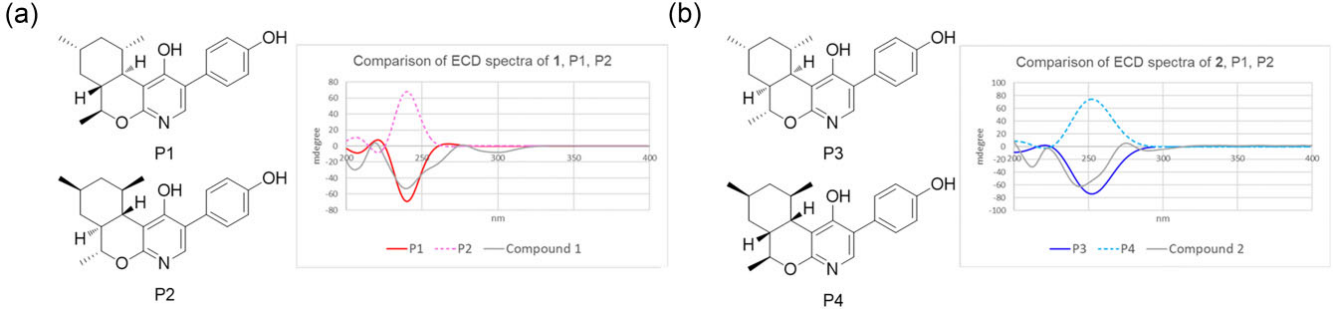
(a) Comparison of ECD for **1** with two possible stereoisomers P1 and P2. (b) Comparison of ECD for **2** with two possible stereoisomers P3, and P4.

To establish the relative configurations of the chiral carbons from C-7–C-13 in the aliphatic ring, ROESY analysis was performed. Strong ROESY correlations for H-7/H-13 and H-12/H_3_-14 indicated that the two rings were in *trans* configuration, and H-12 and H_3_-14 were on the same face of the molecule. In addition, the strong ROESY correlations for H_3_-15/H_3_-16, H_3_-15/H-7, and H_3_-16/H-7 indicated that the two methyl groups on the cyclohexane ring and methine H-7 were in the same direction (Fig. 3). The absolute configuration of the chiral centers in compound **1** was determined by comparing its ECD data with the calculated spectra (Mandi & Kurtan, 2019). Based on the relative configuration obtained from the ROESY data, we predicted two possible enantiomers: P1 with 7*R*8*S*10*R*12*R*13*S* and P2 with 7*S*8*R*10*S*12*S*13*R*). The calculated Cotton effect of P1 with the 7*R*8*S*10*R*12*R*13*S* configuration matches well with the experimental ECD data (Fig. 4a).

Based on the (+) HR-ESI-MS and NMR data, the molecular formula of compound **2** was established to be C_21_H_25_NO_3_, which was the same as that of **1**. Furthermore, **2** had ^1^H and ^13^C NMR patterns highly similar to those of **1**, suggesting that it is a stereoisomer of **1**. Comparison of the ^1^H NMR data of **2** with that of **1** showed that it had different splitting pattern of H-7 (dd, *J* = 11.0, 3.8 Hz) and lower chemical shift of H-13 (*δ*_H_ 4.79). Detailed analysis of the 1D and 2D NMR spectra of **2** confirmed that **2** has the same planar structure as **1** (Fig. 2). The relative configuration of **2** was established by analyzing the ROESY correlations of *sp*^3^ methines in the cyclohexane ring of **2**. The strong ROESY correlation between H-7 and H-12 suggested *cis* configuration of the ring junction (Fig. 3). Additional ROESY correlations for H-13/H-9, H-13/H-10, H_3_-14/H-12, and H-7/H_3_-16 demonstrated that all secondary methyl groups were in the same direction as the methine protons H-7 and H-12 (Fig. 3). To establish the absolute configuration of **2**, we compared the experimental ECD spectrum of **1** with the computationally calculated spectra of the two enantiomers, P3 and P4. The absolute stereochemical description of the chiral centers in **2** was confirmed to be 7*R*8*R*10*S*12*R*13*S*, because the calculated Cotton effect of P3 matched well with the experimental ECD data (Fig. 4b).

The structures of **1** and **2** only differed from that of tolypyridone A (**5**) and tolypyridone I (**6**) in the cyclization of the pyran ring, while the oxygen at C-4 of pyridone was connected to C-13 in **5** and **6**, the oxygen at C-2 was used to generate the pyran ring in **1** and **2**. In comparison of UV spectra of four compounds, while **1** and **2** showed strong UV absorbance at 209, 241, and 272 nm, **5** and **6** had λ_max_ at 215 and 252 nm (Supplementary Fig. S34). This indicated new compounds **1** and **2** have a different UV chromophore system from those of tolypyridone A (**5**) and tolypyridone I (**6**). Furthermore, by careful analysis of ROESY spectra of four compounds, we could determine certainly the new compounds’ cyclization of the side chain in the opposite direction. In ROESY spectra of **1** and **2**, methyl group CH_3_-16 showed remarkable correlation with methine protons H-2' or H-6' in phenol ring. On the other side, in ROESY spectra of **5** and **6**, different methyl group, CH_3_-14, correlated strongly with methine protons H-2' or H-6' in phenol ring (Fig. 3). This evidence could prove that **1** and **2** had different cyclization of decalin ring in the structure from those of tolypyridone A (**5**) and I (**6**). To assist the elucidation of planar structures of **1** and **2**, we compared the ^1^H-NMR data of four compounds which measured in DMSO-*d*_6_ to check exchangeable protons. Only OH-4' was showed in the ^1^H NMR spectrum, and we found big difference at the point of existence of *N*H signal (Fig. S35). Tolypyridone A (**5**) and tolypyridone I (**6**) showed each singlet *N*H peak at 10.98 and 11.01 ppm respectively; however, new compounds **1** and **2** did not have any peak around the similar chemical shift, as we expected (Supplementary Table S2).

**Fig. 5.**
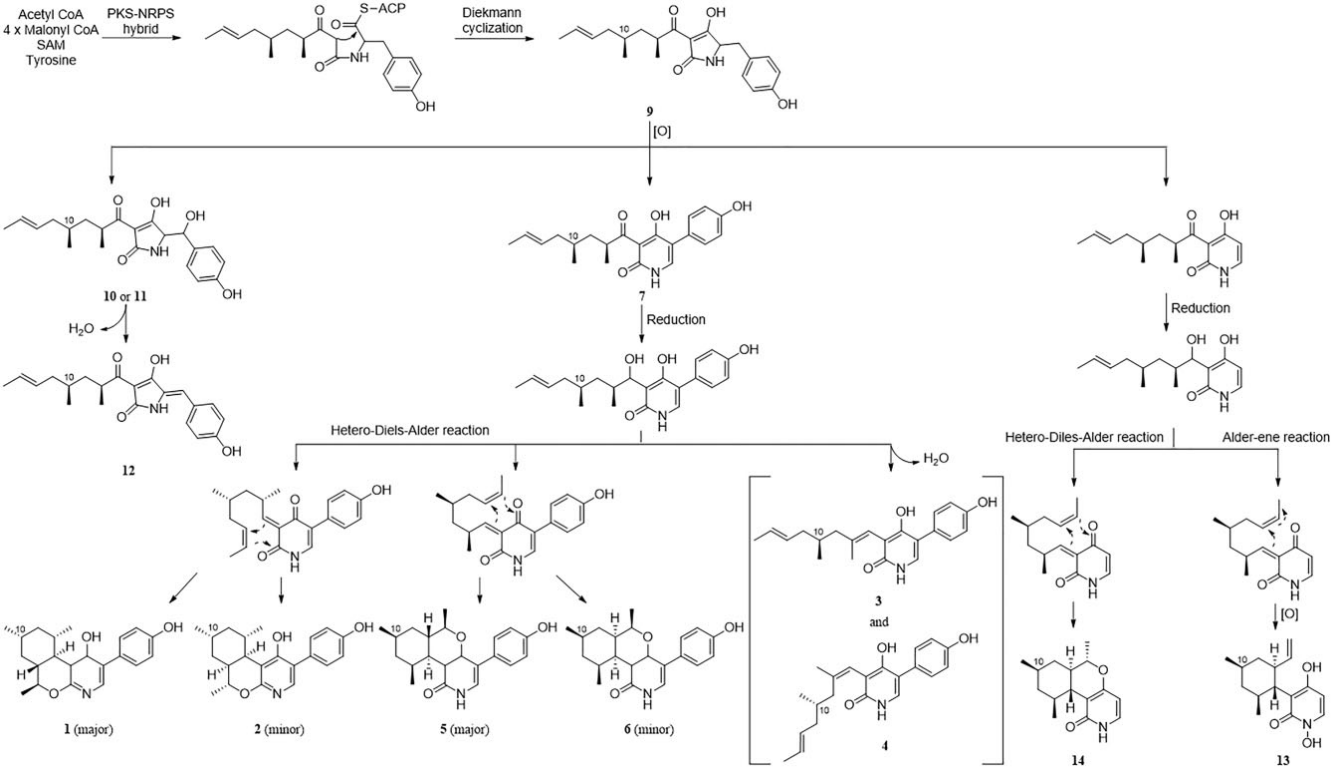
Putative biosynthetic pathway of the isolated compounds.

Compound **3** was isolated as a colorless gel. Its molecular formula was established as C_21_H_25_NO_3_ (10 unsaturations) using (+)HRESIMS (observed at *m/z* 339.1912 [M + H]^+^, calculated for C_21_H_25_NO_3_,339.1907). Analysis of ^1^H, ^13^C, and HSQC NMR data for **3** revealed that it has seven double bonds, one non-oxygenated *sp*^3^ methine, two non-oxygenated methylenes, three methyl groups, and one amide carbonyl group (Table 1). Because ^1^H and ^13^C NMR data were highly similar to those of tolypyridone C (**7**), **3** was predicted to have a 4-hydroxy-2-pyridone core structure. One methyl group attached to an sp^3^ methine carbon (C-8) and one ketone group (C-7) disappeared in **7**. Instead, a methyl group attached to an sp^2^ carbon and a subsequent olefinic group appeared in **3**. Enolization, hydration, and subsequent dehydration can occur between C-7 and C-8 to generate **3**. The complete structure was confirmed using 1D and 2D NMR analyses. Interpretation of the ^1^H-^1^H COSY spectrum provided a spin system, CH_3_-CH = CH-CH_2_-CH(CH_3_)-CH_2_ corresponding to C14-C13 = C12-C11-C10(C15)-C9, which was connected to the methyl-bearing double-bonded carbon C-8 by HMBC of the methylene protons (H_2_-9) with C-7 and C-8 and of the methyl protons (H_3_-16) with C-7, C-8, and C-9. Further HMBC of H-7 with C-2 (*δ*_C_ 165.2), C-3 (*δ*_C_ 111.6), and C-4 (*δ*_C_ 164.3) of the 4-hydroxy-2-pyridone demonstrated that the side chain from C-14 to C-7 was linked to C-3 of the pyridone (Fig. 2). Based on strong ROESY correlations for H-12/H_3_-14 and H-7/H_3_-16, the geometry of the double bonds in the side chain was determined to be *trans* for C-12 and C-13 and *cis* for C-7 and C-8, respectively. The presence of a *para*-substituted phenyl group was evident based on the characteristic symmetric ^1^H and ^13^C resonances, which were connected to C-5 of the 4-hydroxy-2-pyridone moiety by strong HMBC of H-6 with C-1' and of H-2' and 6' with C-5. The absolute configuration of the chiral center, C-10, was difficult to be determined on the experimental ground. We attempted to establish the absolute configuration of the chiral center C-10 in **3** by comparing its ECD data with the computational spectra. More than 30 conformers were obtained for each enantiomer, P1(10*S*) and P2(10*R*), as shown in the supplementary information. Despite this large number, we performed ECD calculations for all conformers. However, the calculated ECD data did not match the experimental results, possibly because the chiral center is located in a region not significantly influenced by the chromophores. Therefore, a quantum mechanics-based DP4 + analysis for compound **3**, comparing the 10*S* (isomer 1) and 10*R* (isomer 2) configurations, was employed, which subsequently yielded reasonable results. Conformers of two enantiomers were optimized using DFT(B3LYP/6-31G(d, p) and their shielding tensors for the optimized conformers were averaged according to Boltzmann weighting (Grimblat et al., 2015). The DP4 + analysis indicated a 99.34% probability that isomer 1 adopts the 10S configuration, as shown in Fig. S36. Since DP4 + primarily predicts relative stereochemistry rather than absolute configuration, we also examined the absolute configurations of C-10 in biosynthetically related compounds. Our analysis confirmed that C-10 consistently adopts the *S* configuration. Thus, the complete structure of **3** was established (Fig. 2). We proposed the biosynthetic pathways of all isolated compounds. Compound **3** is likely derived from compound **7** through a sequence of reduction and dehydration steps. Given that all known compounds in the proposed pathway including compound **7**, and the newly identified compounds **1** and **2** share the same stereochemistry at C-10, we infer that compounds **3** and **4** also retain the same configuration at this position (Fig. 5).

Compound **4** was also isolated as a colorless gel and had the molecular formula C_21_H_25_NO_3_ similar to that of **3**, based on (+)HRESIMS data. The UV spectrum of **4** showed a pattern analogous to that of **3**, with two large absorptions at 209 and 254 nm. The ^1^H and ^13^C NMR data for **4** were very similar to those of **3**, except for the resonances of C-8 and H_3_-16/C-16. Interpretation of the 1D and 2D NMR spectra suggests geometrical isomerism between **3** and **4**. While a strong ROESY correlation was observed between H-7 and H_2_-9a in **3**, a strong ROESY correlation was observed between H-7 and H_3_-16 in **4**, suggesting different geometries of the double bond at C-7 and C-8 (Fig. 2). Thus, the geometry of C-7 was determined to be *cis*, which explains remarkably different chemical shift of C-8 and C-16 (Table 1).

### Identification of Neuroprotective Compounds for Treating PD

**Fig. 6.**
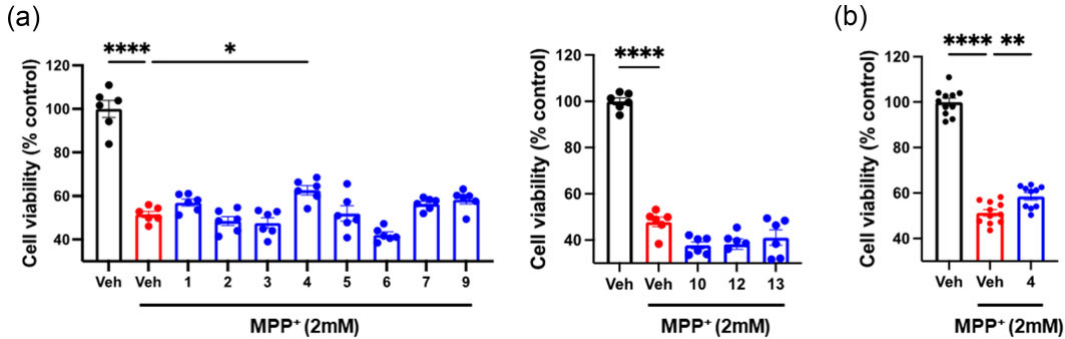
Compound **4** exhibits neuroprotective properties in an *in vitro* PD model. (a) Of the 11 compounds screened, compound **4** shows a protective effect in an *in vitro* PD model (** *p* < 0.01, **** *p* < 0.0001, one-way ANOVA with Tukey's post hoc test). (b) Pretreatment of compound **4** protects SH-SY5Y cells against MPP^+^-induced cell death (** *p* < 0.01, **** *p* < 0.0001, one-way ANOVA with Tukey's post hoc test).

1-Methyl-4-phenylpyridinium, MPP^+^, a Parkinsonian neurotoxin that induces dopaminergic neuronal cell death, was used to identify neuroprotective compounds in an *in vitro* PD model. We tested 11 isolated compounds and found that among them, compound **4** significantly increased cell viability compared with MPP^+^-treated cells (Fig. 6a). To validate this screening, we pretreated cells with 10 µmol/L of compound **4** for 24 hr, followed by incubation with 2 mmol/L of MPP^+^ for an additional 24 hr. Treatment with MPP^+^ induced SH-SY5Y cell death; however, pretreatment with compound **4** attenuated this effect (Fig. 6b), suggesting that it has therapeutic potential for the treatment of PD.

### Evaluation of the Anti-Inflammatory Effects of Compound 3 in an *in Vitro* Model of Parkinson's Disease

**Fig. 7.**
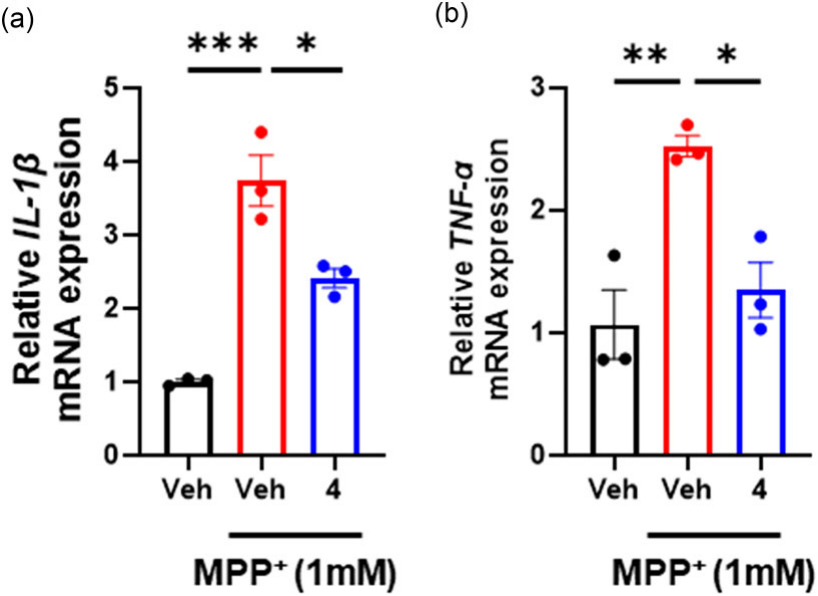
Compound **4** shows anti-inflammatory effects in an *in vitro* PD model. (a) Compound **4** attenuates MPP⁺-induced IL-1β expression in SH-SY5Y cells (*p < 0.05, *** *p* < 0.001, one-way ANOVA with Tukey's post hoc test). (b) Compound **4** reduces MPP⁺-induced TNF-α expression in SH-SY5Y cells (*p < 0.05, **p < 0.01, one-way ANOVA with Tukey's post hoc test). Data represent the mean ± SEM from three independent experiments.

The neurotoxin MPP^+^ induces neuroinflammation, which plays a pivotal role in the pathogenesis of PD (Bae et al., 2022). To further evaluate the potential of compound **4**, we examined its anti-inflammatory activity in the *in vitro* PD model. Exposure to MPP^+^ significantly upregulated the expression of pro-inflammatory cytokines, including IL-1β and TNF-α, in SH-SY5Y cells. However, pretreatment with compound **4** (10 µmol/L) for 24 hr significantly reduced the levels of IL-1β and TNF-α (Fig. 7a and b), indicating that compound **4** exerts anti-inflammatory effects in this model.

## Conclusions

Chemical investigation of *Tolypocladium* sp. (strain CNC14) extracts targeting 4-hydroxy-2-pyridone alkaloids using our in-house UV library led to the isolation of four new (**1**–**4**) and ten known (**5**–**14**) compounds. All the isolated compounds contained tetramic acid or 4-hydroxy-2-pyridone moieties, which reportedly have diverse biological activities. Based on previous research, we predicted their biosynthetic pathways, starting with a tetramic acid alkaloid, tolypoalbin (**9**), which is biosynthesized by a PKS-NRPS hybrid, followed by Diekmann cyclization. The enzymatic oxidation of **9** yielded three pathways: hydroxylation, ring expansion of the tetramate ring, and dephenylation (Wasil et al., 2013). Compound **12** was derived by dehydration in compounds **10** and **11** (Zhang et al., 2020). The ring expansion of the tetramate in **9** can generate tolypyridone C (**7**), which is predicted to be reduced and dehydrated to afford compounds **3** and **4**, further hetero-Diels–Alderized to generate compounds **1** and **2** (Fig. 5). The hetero-Diels–Alder reaction might have occurred for cyclization in the opposite direction in **1** and **2** to that of tolypyridone A (**5**) (Zhang et al., 2020). Pyridoxatin (**13**) and compound **14** were synthesized through hetero-Diels–Alder and Alder-ene reactions, respectively (Fig. 5) (Ohashi et al., 2020). Among the 11 isolated compounds, compound **4** exhibits anti-Parkinson's effects. However, the current study has several limitations, including the lack of in vivo experiments and an incomplete understanding of the mode of action of compound **4** in neuroprotective mechanisms. Therefore, further in vivo studies, as well as the identification of compound **4**’s targets, pharmacodynamics, and pharmacokinetics, are necessary for its development as an anti-Parkinson's drug.

## Supplementary Material

kuaf027_Supplemental_File
