# Supplementary material for: Anti-Parkinsonian 4-hydroxy-2-pyridones from an endolichenic fungus, Tolypocladium sp. (strain CNC14)
Source: J Ind Microbiol Biotechnol. 2025 Sep 3;52:kuaf027. doi: 10.1093/jimb/kuaf027 (PMC12455187; doi:10.1093/jimb/kuaf027)
Supplement: kuaf027_Supplemental_File [file kuaf027_supplemental_file.docx]

**Supplementary Information**

**Anti-Parkinsonian 4-hydroxy-2-pyridones from an endolichenic fungus, *Tolypolcadium* sp. CNC14**

Jin Won Choi ^1,†^, Chaesun Kwon^2,†^, Jin Woo Lee ^3^, Jae-Seoun Hur ^4^, Min-Kyoo Shin,^1,2,*^ and Sang Hee Shim^1,*^

^1^Natural Products Research Institute, College of Pharmacy, Seoul National University, Seoul 08826, Republic of Korea

^2^College of Pharmacy and Research Institute of Pharmaceutical Sciences, Seoul National University, Seoul 08826, Republic of Korea

^3^College of Pharmacy, Duksung Women’s University, Seoul 01369, Republic of Korea

^4^Korean Lichen Research Institute, Sunchon National University, Suncheon 57922, Republic of Korea

*Corresponding authors

E-mail addresses: [minkyooshin@snu.ac.kr](mailto:minkyooshin@snu.ac.kr) (M-K. Shin), [sanghee_shim@snu.ac.kr](mailto:sanghee_shim@snu.ac.kr) (S. H. Shim).

^†^Both authors equally contributed to this work.

**List of Supplementary materials**

**Figure S1.** Targeting 4-hydroxy-2-pyridone alkaloids using in-house UV library.

**Figure S2.** UV spectrum of compound **1** (in MeOH).

**Figure S3.** ^1^H NMR spectrum (800 MHz, CD_3_OD) of compound **1**.

**Figure S4.** ^1^H NMR spectrum (800 MHz, DMSO-*d*_6_) of compound **1**.

**Figure S5.** ^13^C NMR spectrum (200 MHz, CD_3_OD) of compound **1**.

**Figure S6.** ^1^H-^1^H COSY spectrum (800 MHz, CD_3_OD) of compound **1**.

**Figure S7.** HSQC spectrum (800 MHz, CD_3_OD) of compound **1**.

**Figure S8.** HMBC spectrum (800 MHz, CD_3_OD) of compound **1**.

**Figure S9.** ROESY spectrum (800 MHz, CD_3_OD) of compound **1**.

**Figure S10.** HRESIMS spectrum of compound **1**.

**Figure S11.** UV spectrum of compound **2** (in MeOH).

**Figure S12.** ^1^H NMR spectrum (800 MHz, CD_3_OD) of compound **2**.

**Figure S13.** ^13^C NMR spectrum (200 MHz, CD_3_OD) of compound **2**.

**Figure S14.** ^1^H-^1^H COSY spectrum (800 MHz, CD_3_OD) of compound **2**.

**Figure S15.** HSQC spectrum (800 MHz, CD_3_OD) of compound **2**.

**Figure S16.** HMBC spectrum (800 MHz, CD_3_OD) of compound **2**.

**Figure S17.** ROESY spectrum (800 MHz, CD_3_OD) of compound **2**.

**Figure S18.** HRESIMS spectrum of compound **2**.

**Figure S19.** UV spectrum of compound **3** (in MeOH).

**Figure S20.** ^1^H NMR spectrum (800 MHz, CD_3_OD) of compound **3**.

**Figure S21.** ^13^C NMR spectrum (200 MHz, CD_3_OD) of compound **3**.

**Figure S22.** ^1^H-^1^H COSY spectrum (800 MHz, CD_3_OD) of compound **3**.

**Figure S23.** HSQC spectrum (800 MHz, CD_3_OD) of compound **3**.

**Figure S24.** HMBC spectrum (800 MHz, CD_3_OD) of compound **3**.

**Figure S25.** ROESY spectrum (800 MHz, CD_3_OD) of compound **3**.

**Figure S26.** HRESIMS spectrum of compound **3**.

**Figure S27.** UV spectrum of compound **4** (in MeOH).

**Figure S28.** ^1^H NMR spectrum (800 MHz, CD_3_OD) of compound **4**.

**Figure S29.** ^13^C NMR spectrum (200 MHz, CD_3_OD) of compound **4**.

**Figure S30.** ^1^H-^1^H COSY spectrum (800 MHz, CD_3_OD) of compound **4**.

**Figure S31.** HSQC spectrum (200 MHz, CD_3_OD) of compound **4**.

**Figure S32.** HMBC spectrum (800 MHz, CD_3_OD) of compound **4**.

**Figure S33.** ROESY spectrum (800 MHz, CD_3_OD) of compound **4**.

**Figure S34.** HRESIMS spectrum of compound **4**.

**Figure S35.** UV spectra comparison of compounds **1**, **2**, **5**, and **6** (in MeOH).

**Figure S36**. ^1^H-NMR spectra (800 MHz, DMSO-*d*_6_) of **1**, **2**, **5**, and **6**.

**Figure S37**. Conformer calculation and DP4+ calculation result of isomers **1** and **2**.

**Table S1.** Coordinates and energies of the low-energy conformers of **1** and **2** calculated with def2-TZVPP atomic attributes in vacuo.

**Table S2.** ^1^H NMR data of **1**, **2**, **5**, and **6** in DMSO-*d*_6_ at 800 MHz.

**Table S3.** ^1^H and ^13^C NMR data of compound **4** and its isomers.

 **Figure S1**. Targeting 4-hydroxy-2-pyridone alkaloids using in-house UV library. (a) UV spectrum of sambutoxin and tolypyridone A (b) LC-UV profile of extract of *Tolypocladium* sp. (strain CNC14) showed similar UV spectra of **1**-**4** to that of sambutoxin and tolypyridone A. The strain was cultivated on a large scale and extracted using organic solvents. The extracts were subjected to stepwise chromatographic procedures, which isolated fourteen compounds (**1**–**14**), including four new (**1**–**4**) and six known pyridone derivatives (**5**–**8**, **13**, **14**) and four known tetramic acids (**9**–**12**) (Fig. 2). Tolypyridone A (**5**) (Li et al., 2015), tolypyridone I (**6**) (Jung et al., 2023), tolypyridone C (**7**) (Zhang et al., 2020), trichodin B (**8**) (Wu et al., 2014), tolypoalbin (**9**) (Li et al. 2015), F-14329 (**10**) (Li et al., 2015), tolypocladenol B (**11**) (Li et al., 2015), tolypocladenol A (**12**) (Li et al., 2015), pyridoxatin (**13**) (Li et al., 2015), and trimethyl-octahydro-isochromeno-pyridinone (**14**) (Ohashi et al., 2020) were the known compounds identified by comparing their spectroscopic data with those provided in the literature.

**Figure S2.** UV spectrum of compound **1** (in MeOH).


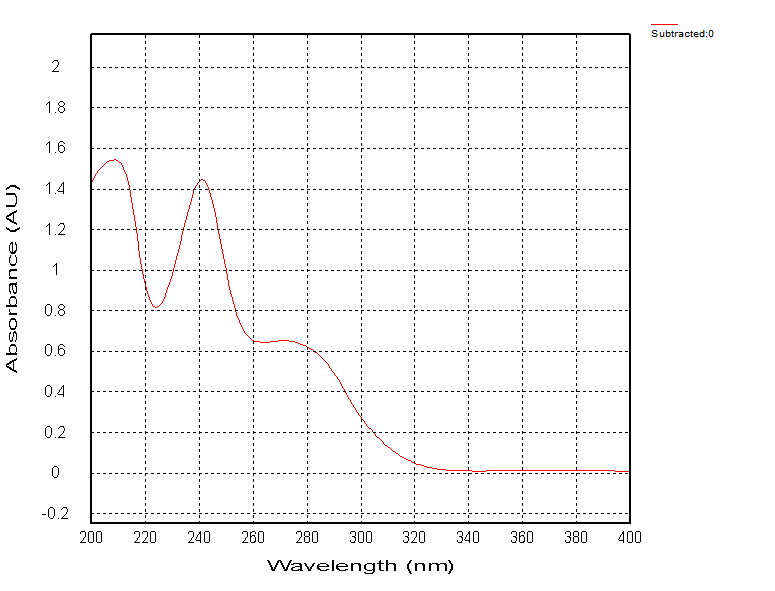


**Figure S3.** ^1^H NMR spectrum (800 MHz, CD_3_OD) of compound **1**.

**
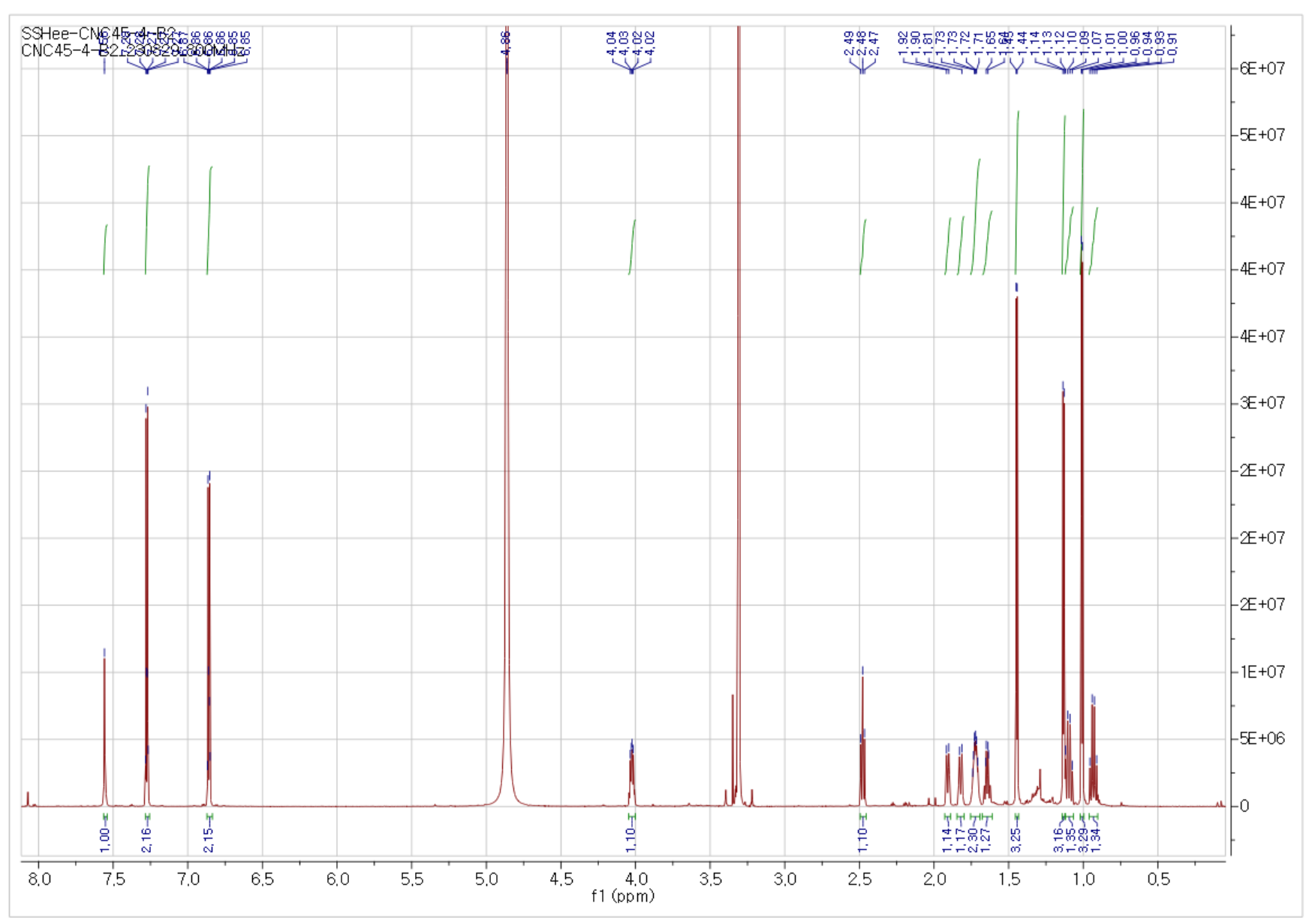
**

**Figure S4.** ^1^H NMR spectrum (800 MHz, DMSO-*d*_6_) of compound **1**.


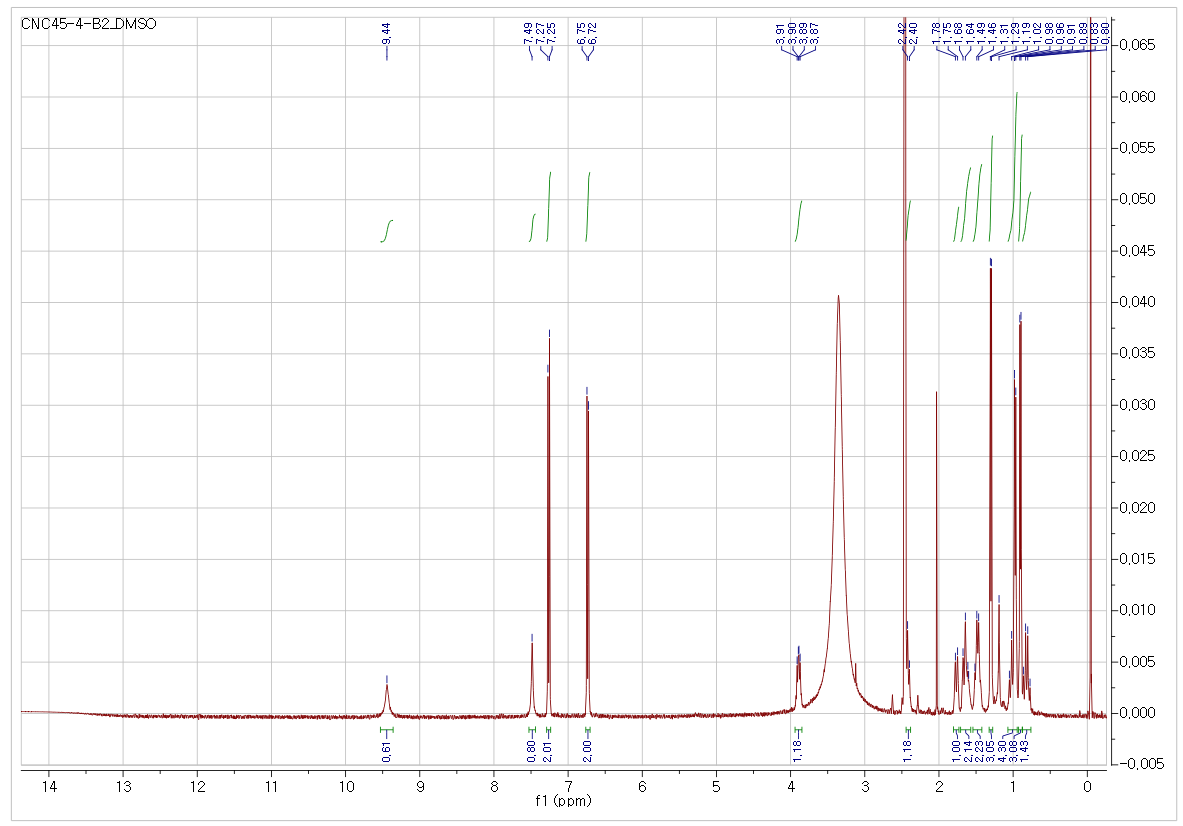


**Figure S5.** ^13^C NMR spectrum (200 MHz, CD_3_OD) of compound **1**.
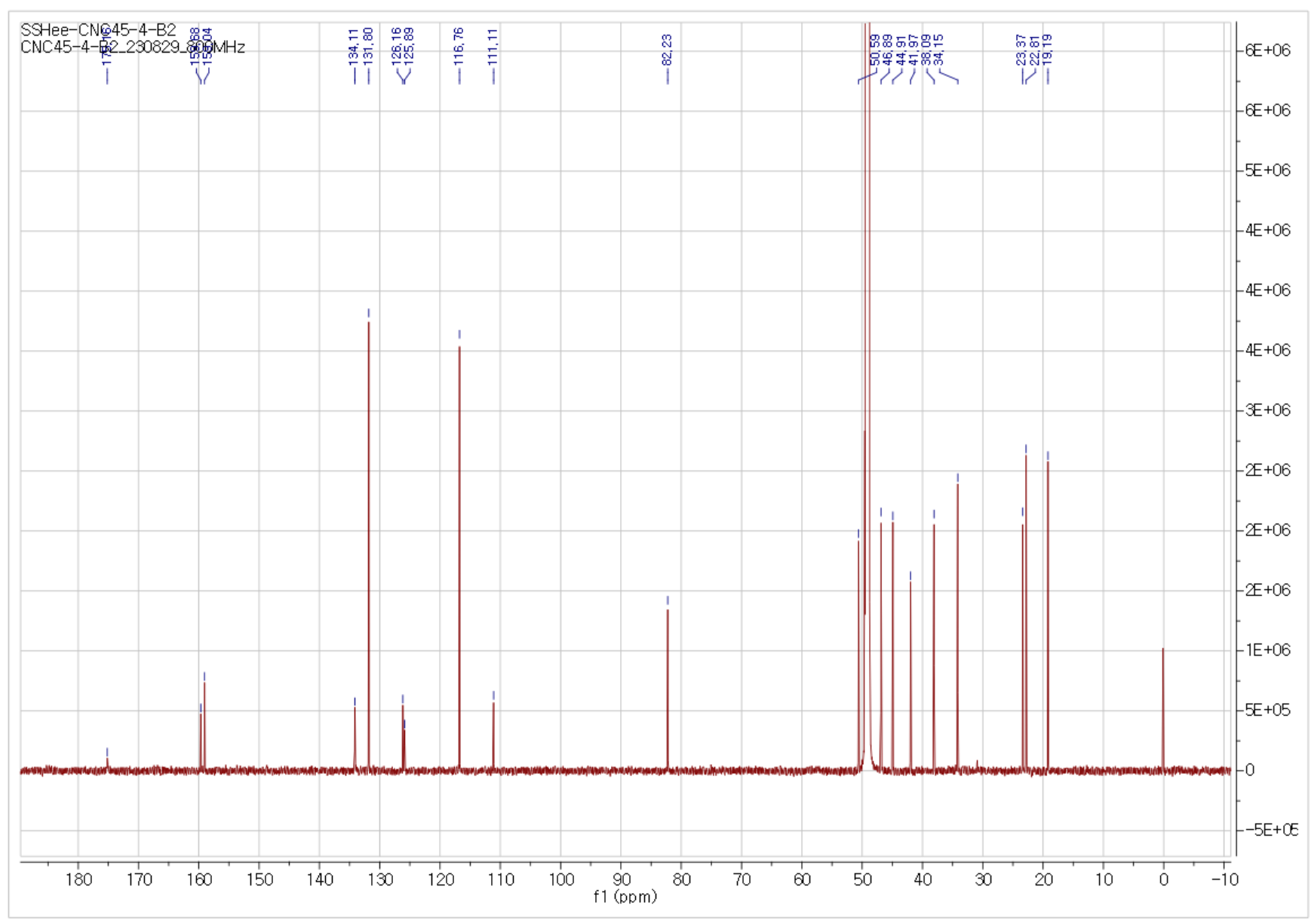


**Figure S6.** ^1^H-^1^H COSY spectrum (800 MHz, CD_3_OD) of compound **1**.


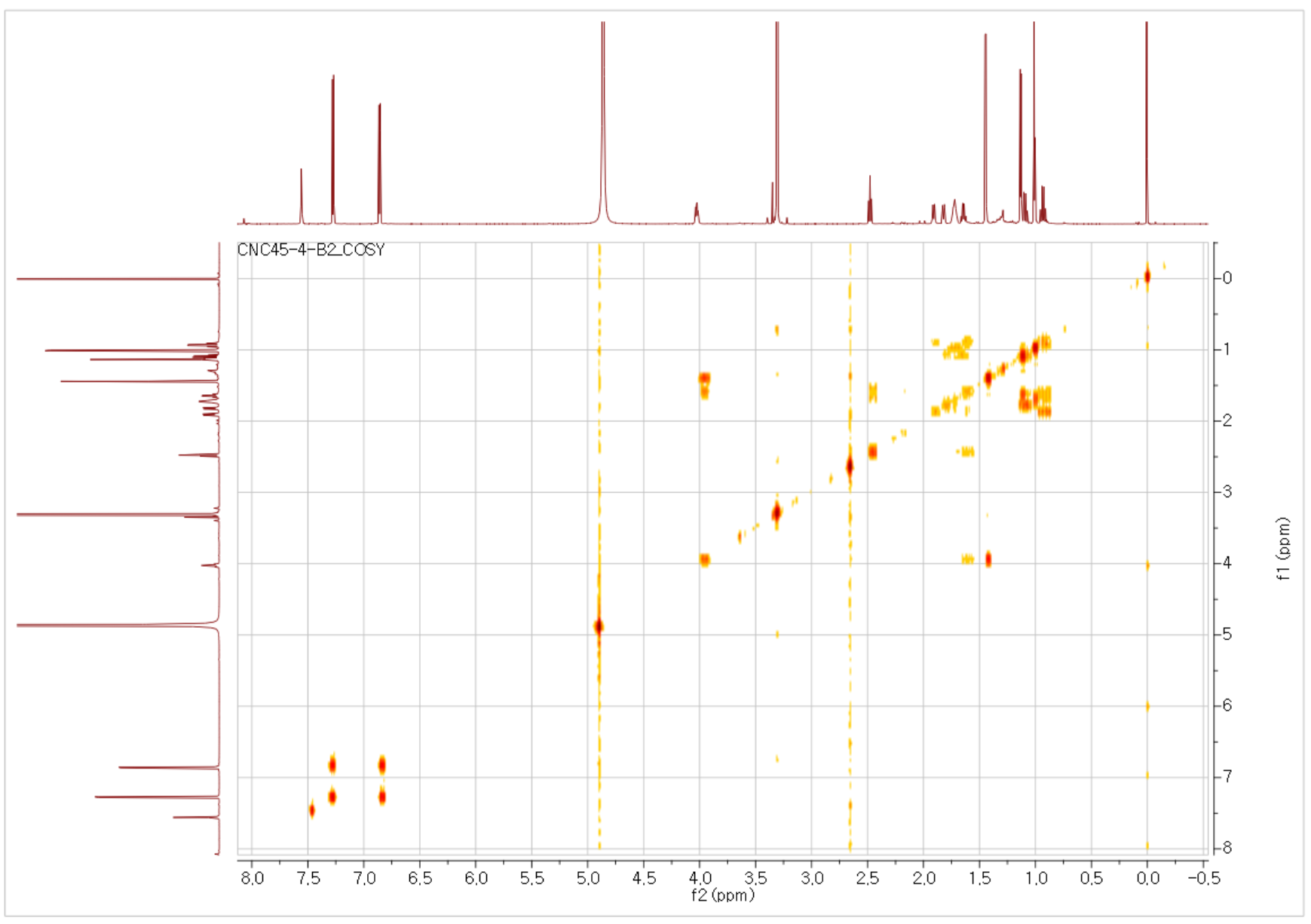


**Figure S7.** HSQC spectrum (800 MHz, CD_3_OD) of compound **1**.**
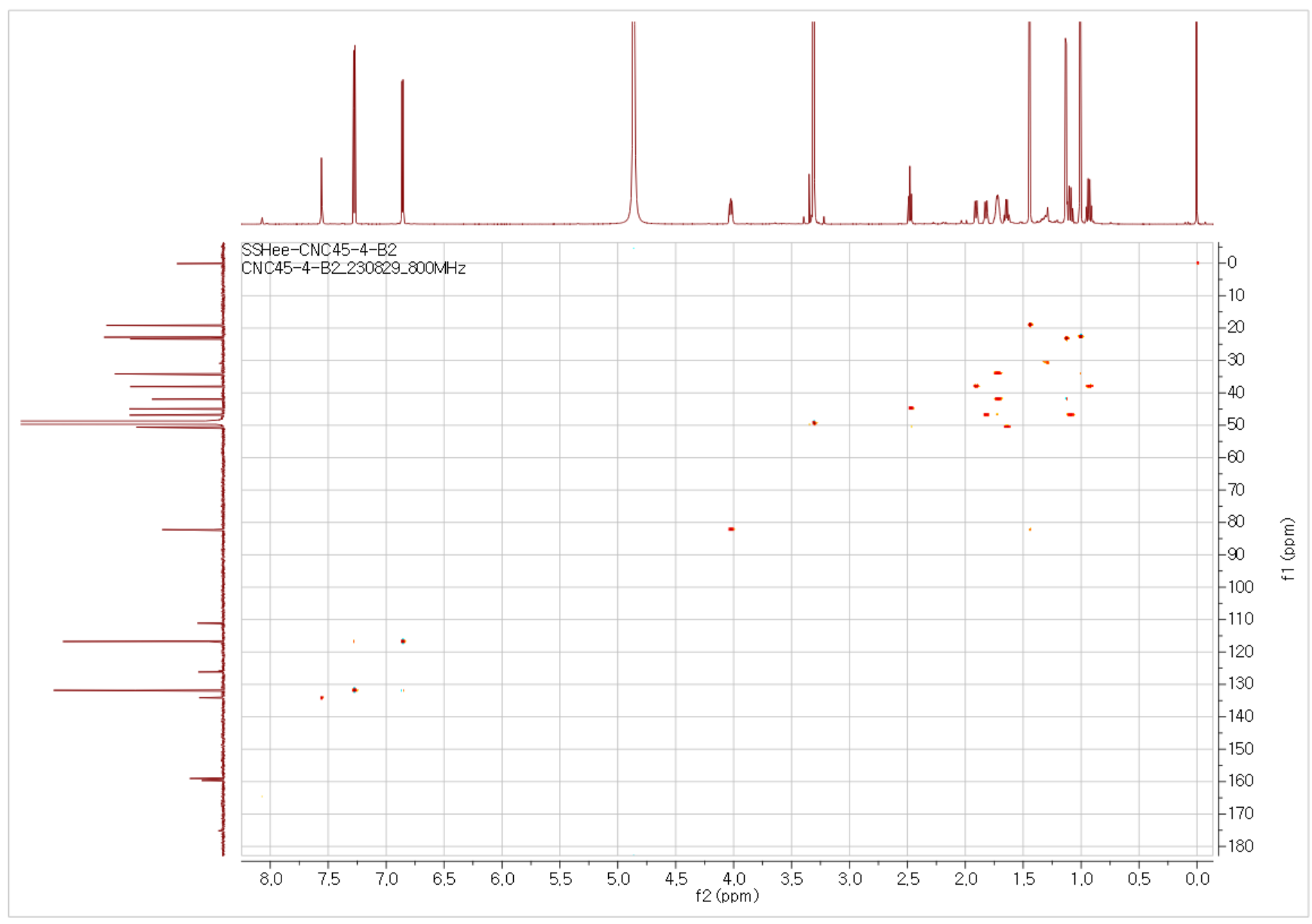
**

**Figure S8.** HMBC spectrum (800 MHz, CD_3_OD) of compound **1**.


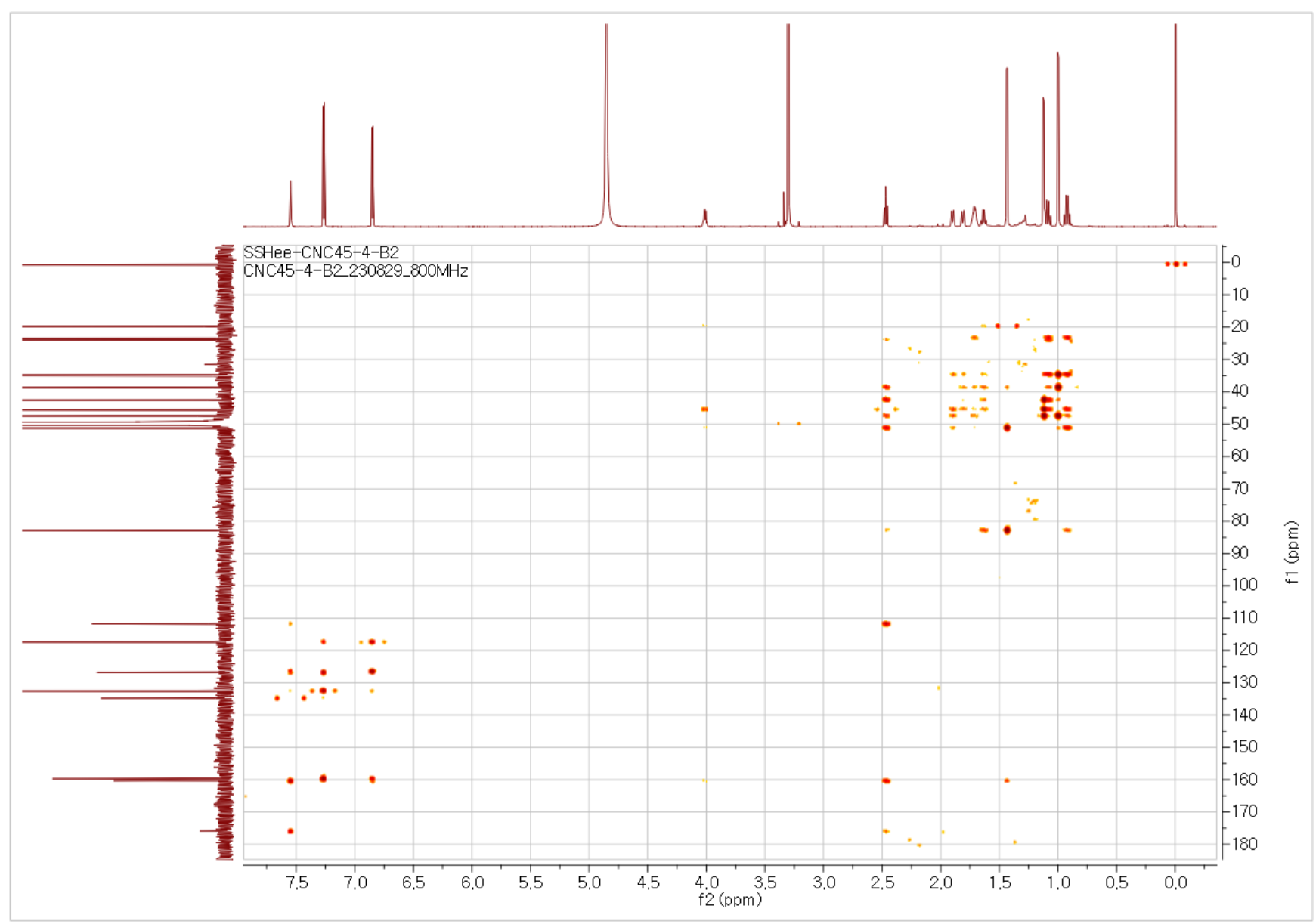


**Figure S9.** ROESY spectrum (800 MHz, CD_3_OD) of compound **1**.


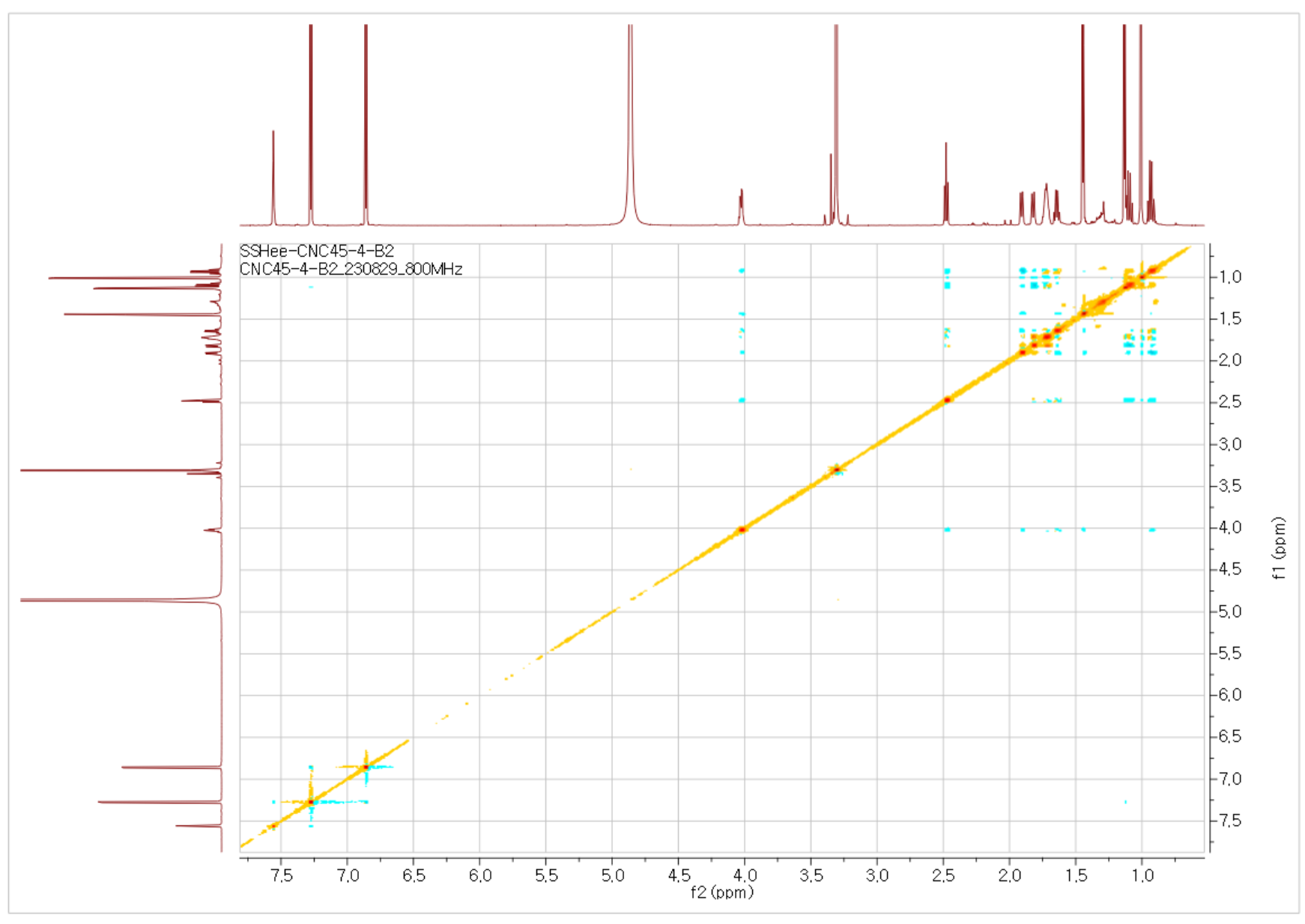


**Figure S10.** HRESIMS spectrum of compound **1**.


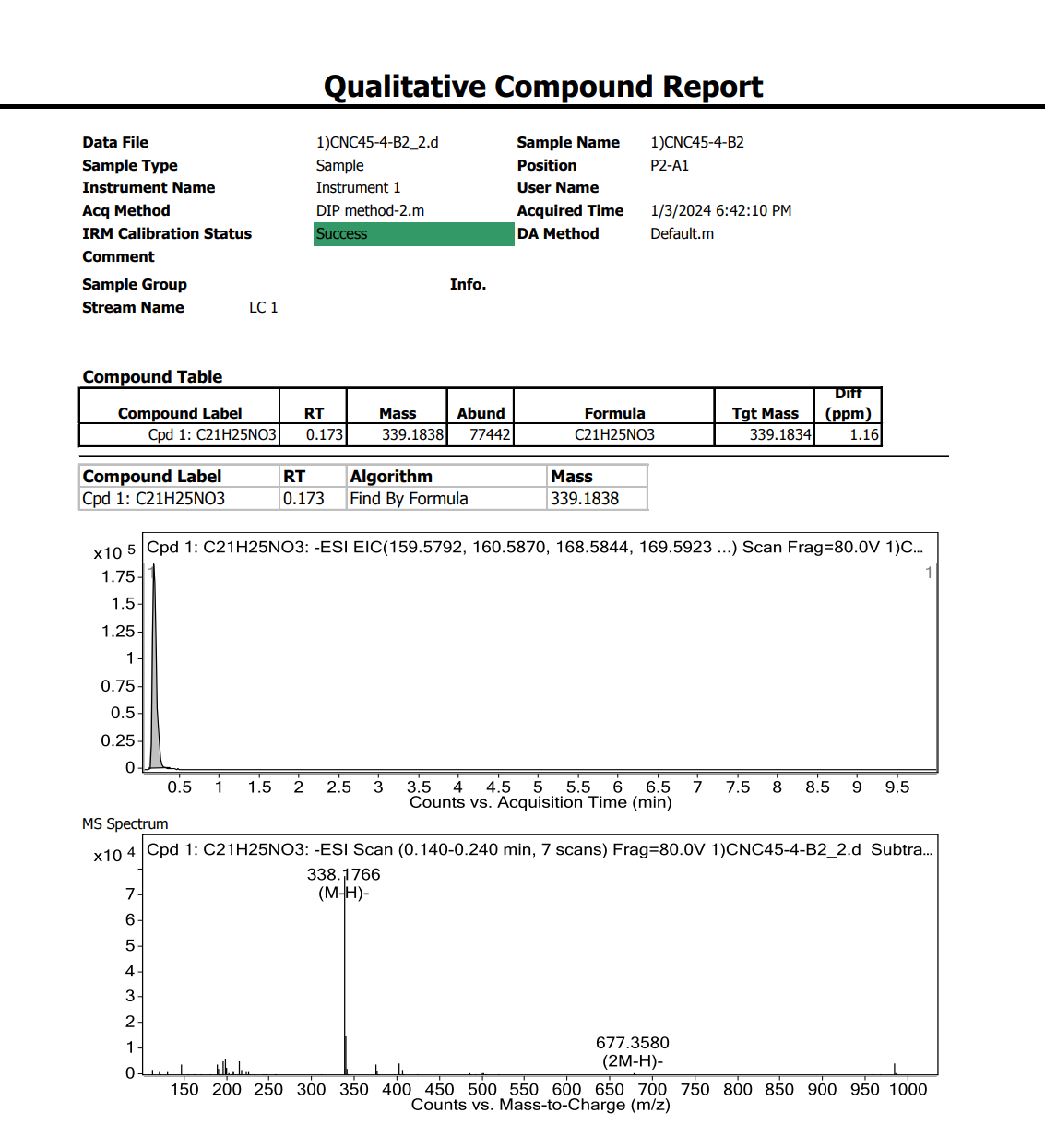


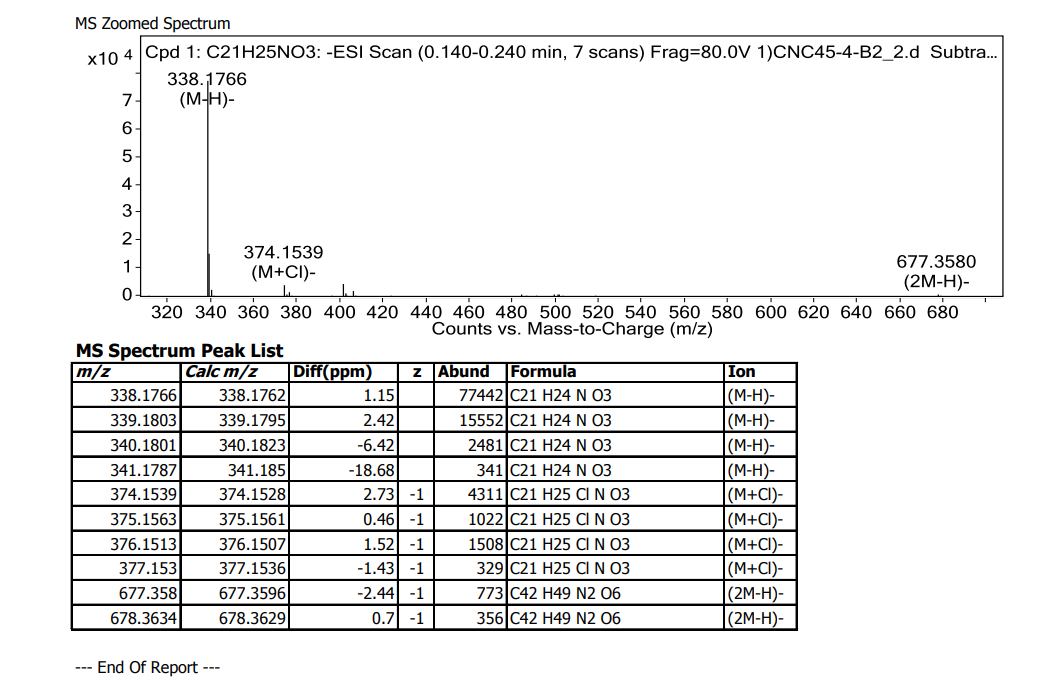


**Figure S11.** UV spectrum of compound **2** (in MeOH).


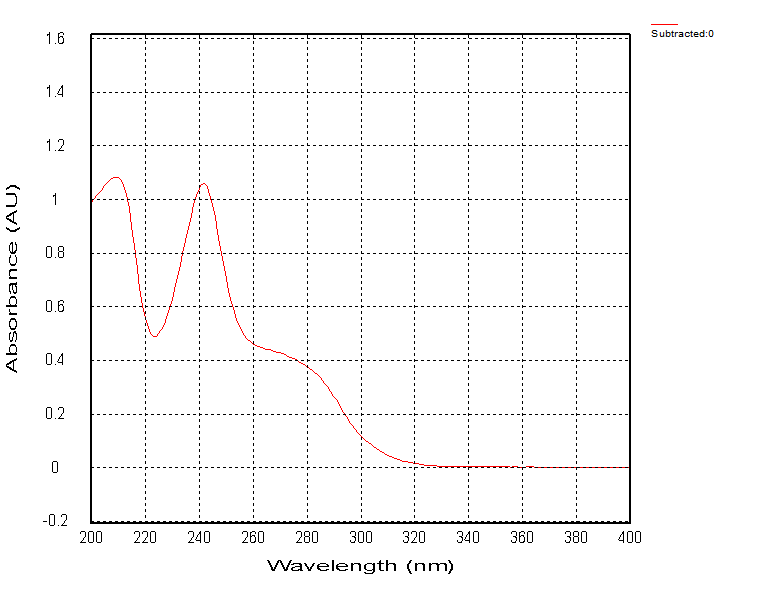


**Figure S12.** ^1^H NMR spectrum (800 MHz, CD_3_OD) of compound **2**.

**
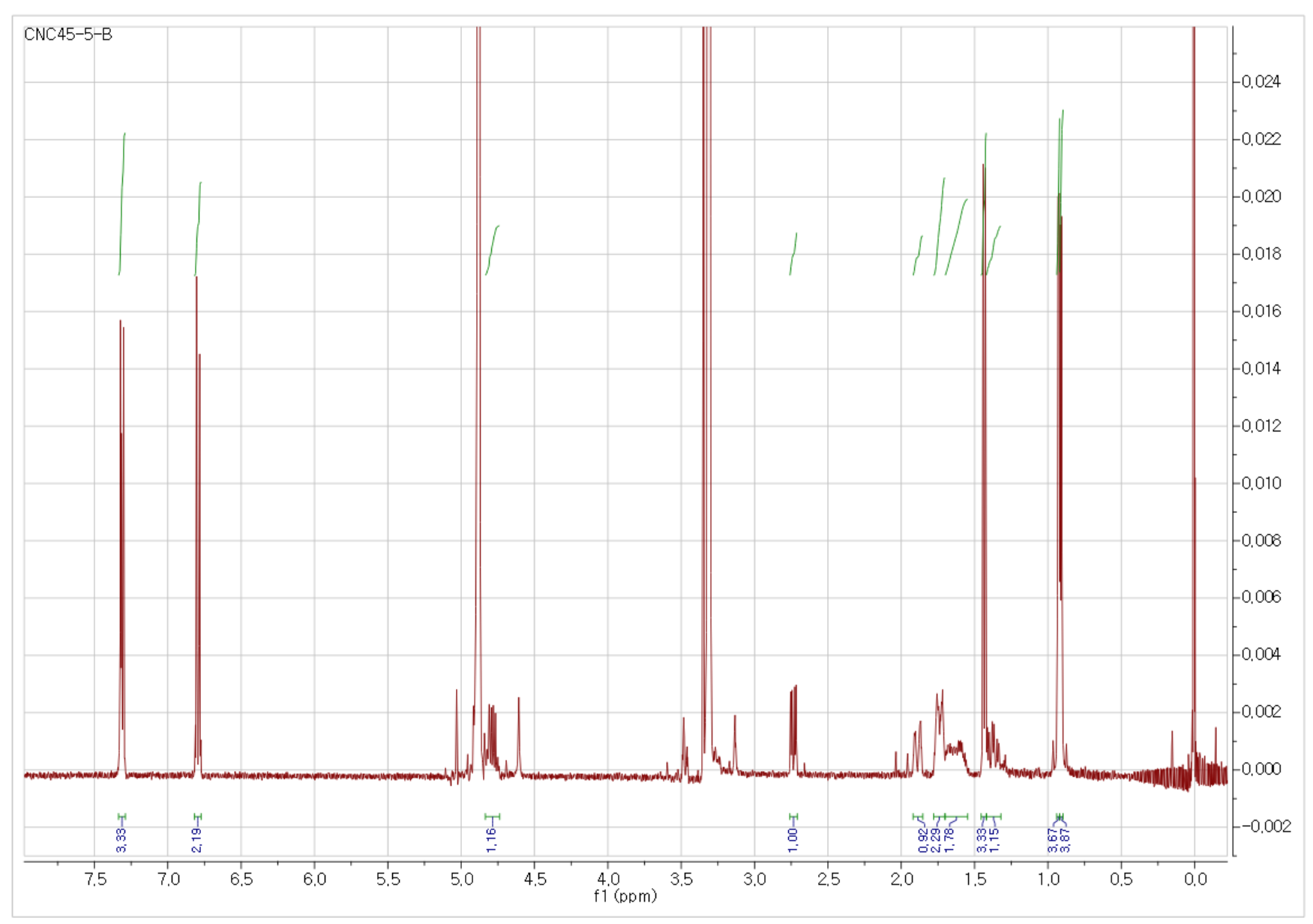
**

**Figure S13.** ^13^C NMR spectrum (200 MHz, CD_3_OD) of compound **2**.


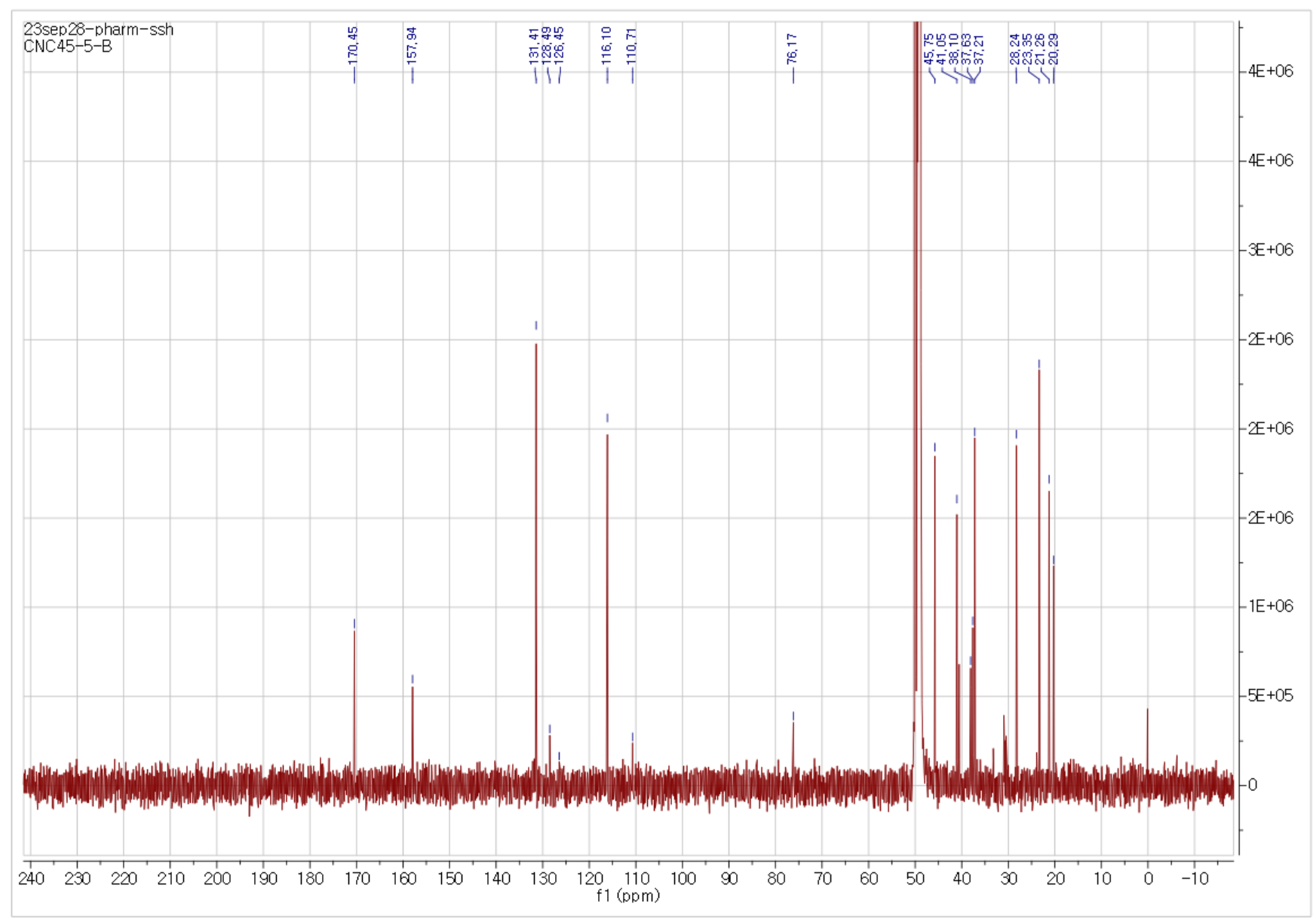


**Figure S14.** ^1^H-^1^H COSY spectrum (800 MHz, CD_3_OD) of compound **2**.


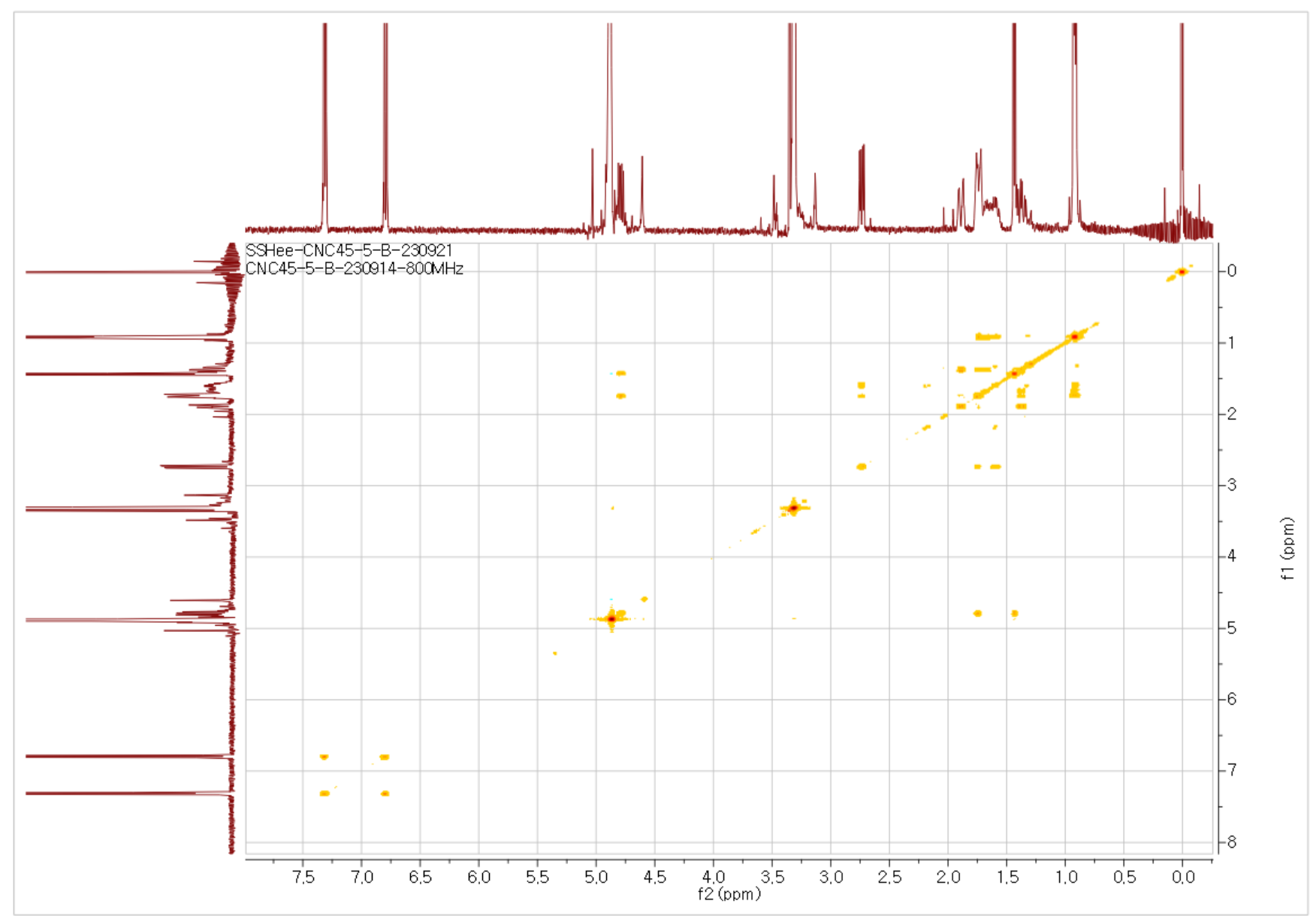


**Figure S15.** HSQC spectrum (800 MHz, CD_3_OD) of compound **2**.

**
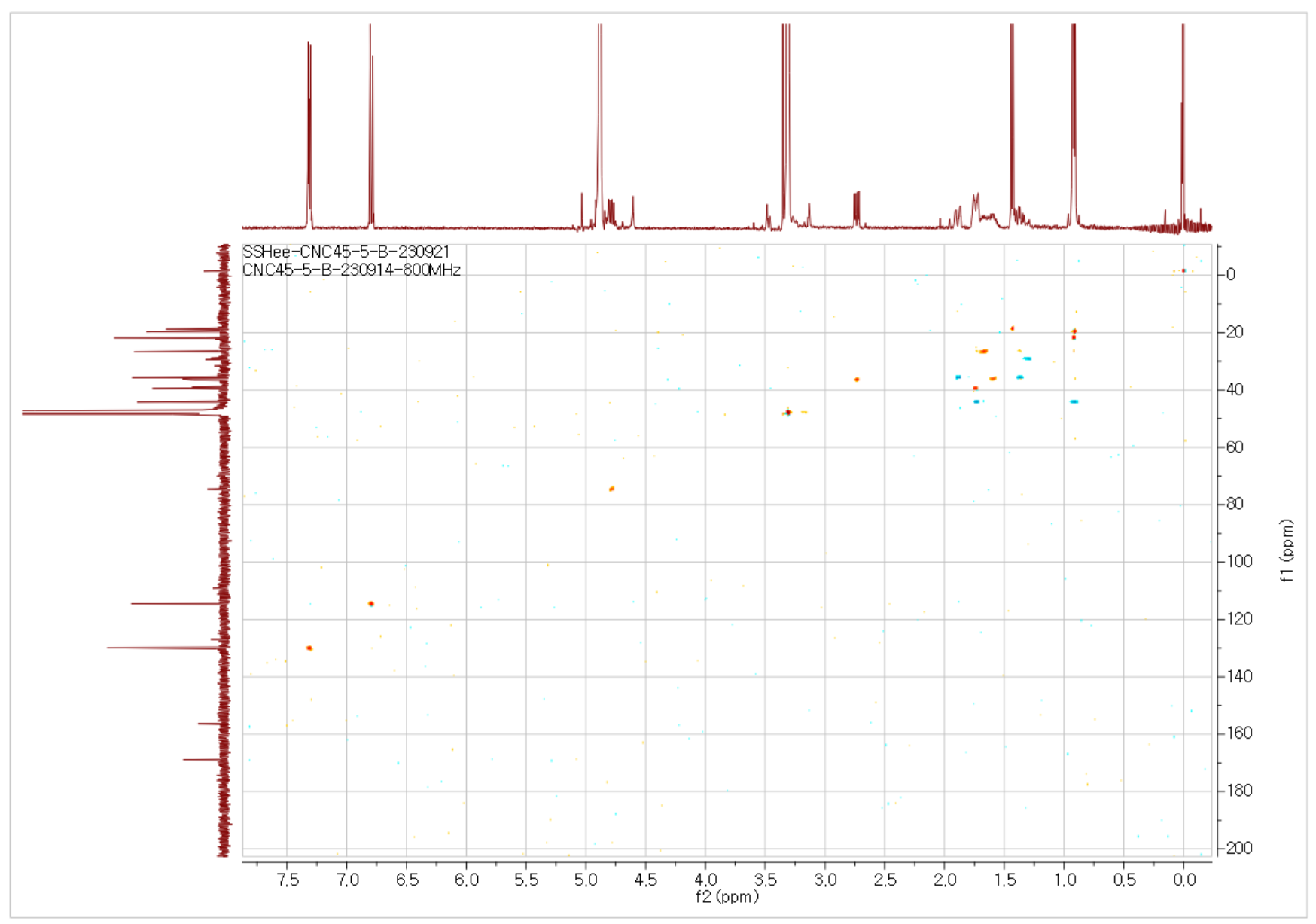
**

**Figure S16.** HMBC spectrum (800 MHz, CD_3_OD) of compound **2**.**
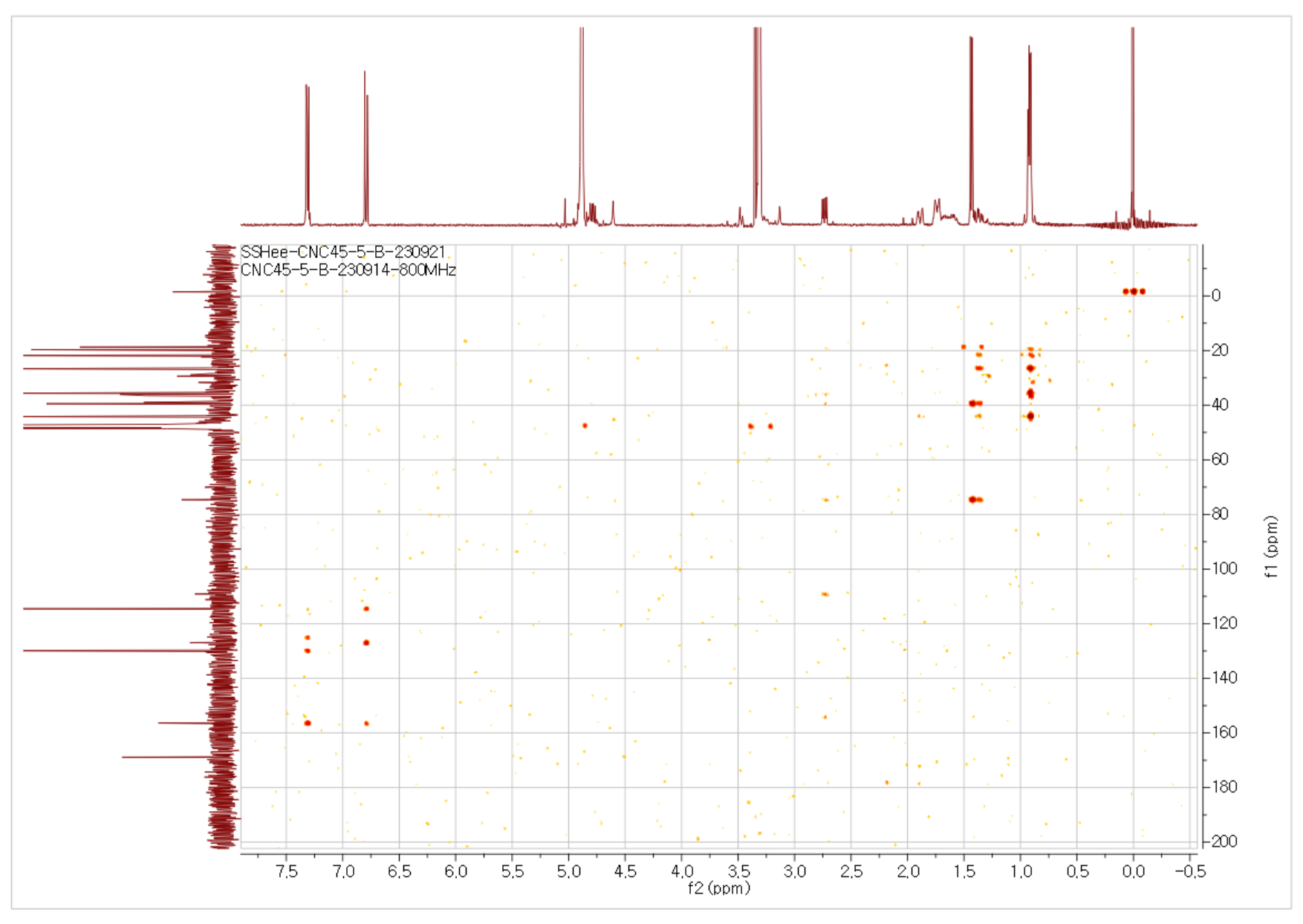
**

**Figure S17.** ROESY spectrum (800 MHz, CD_3_OD) of compound **2**.

**
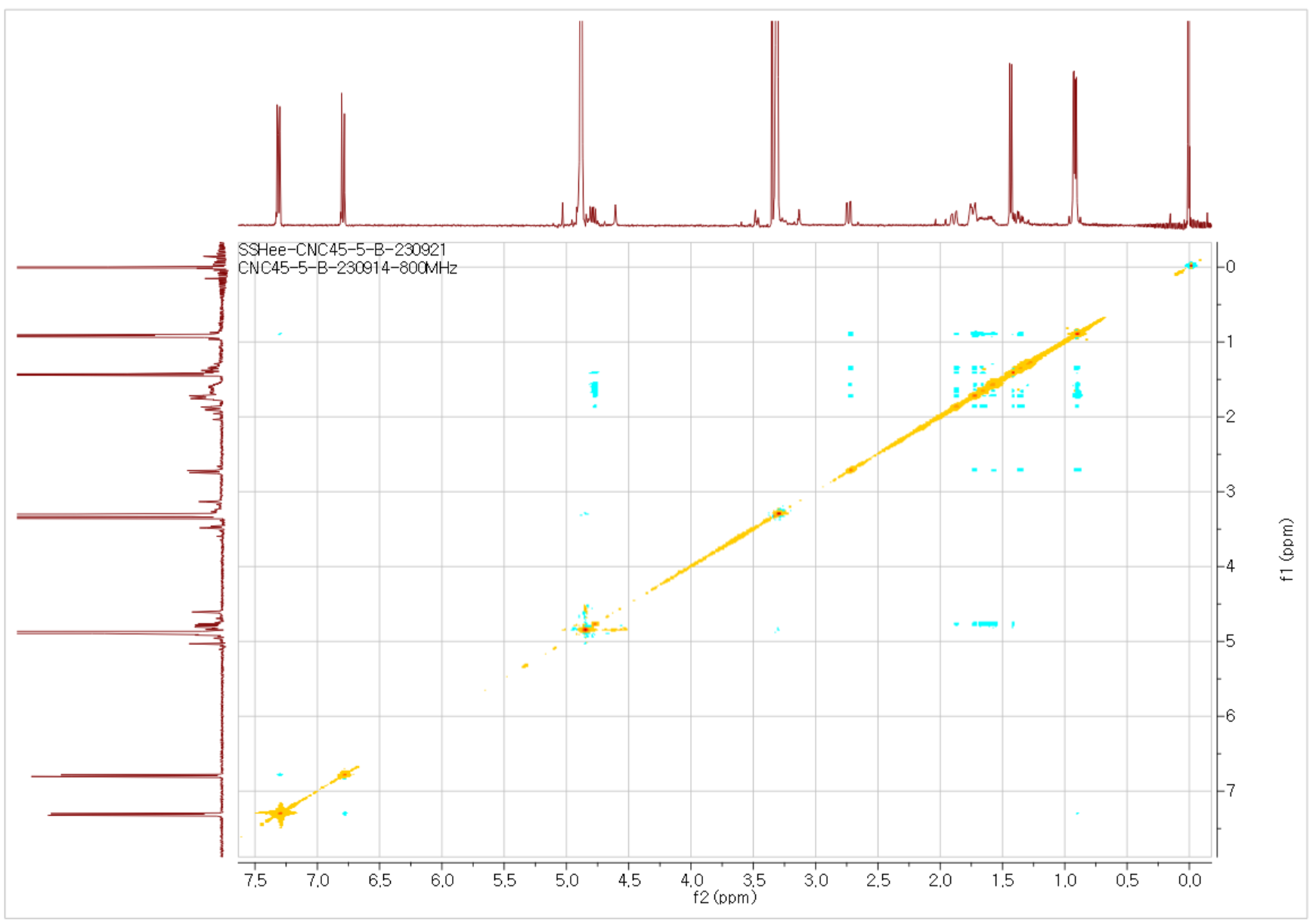
**

**Figure S18.** HRESIMS spectrum of compound **2**.


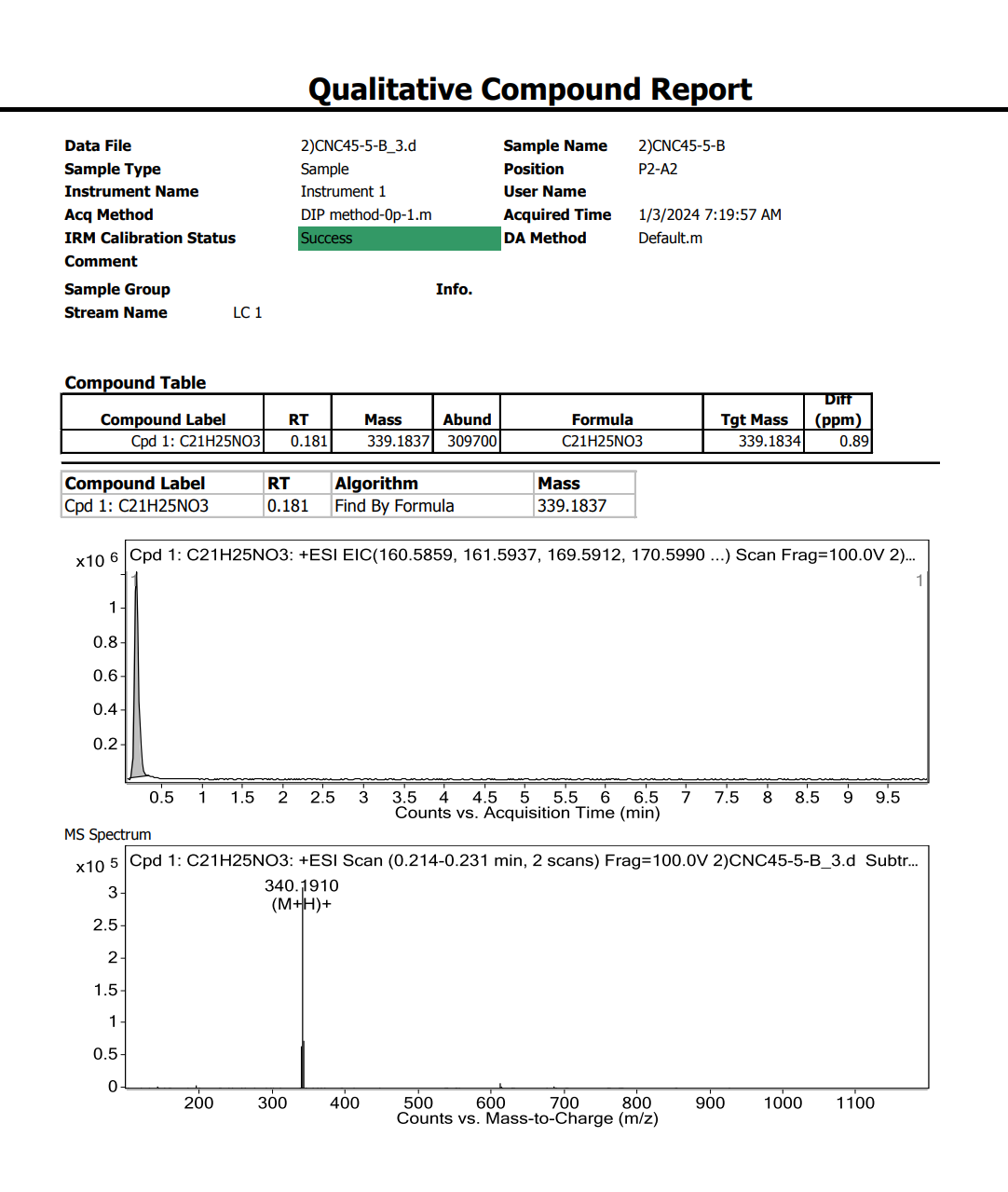

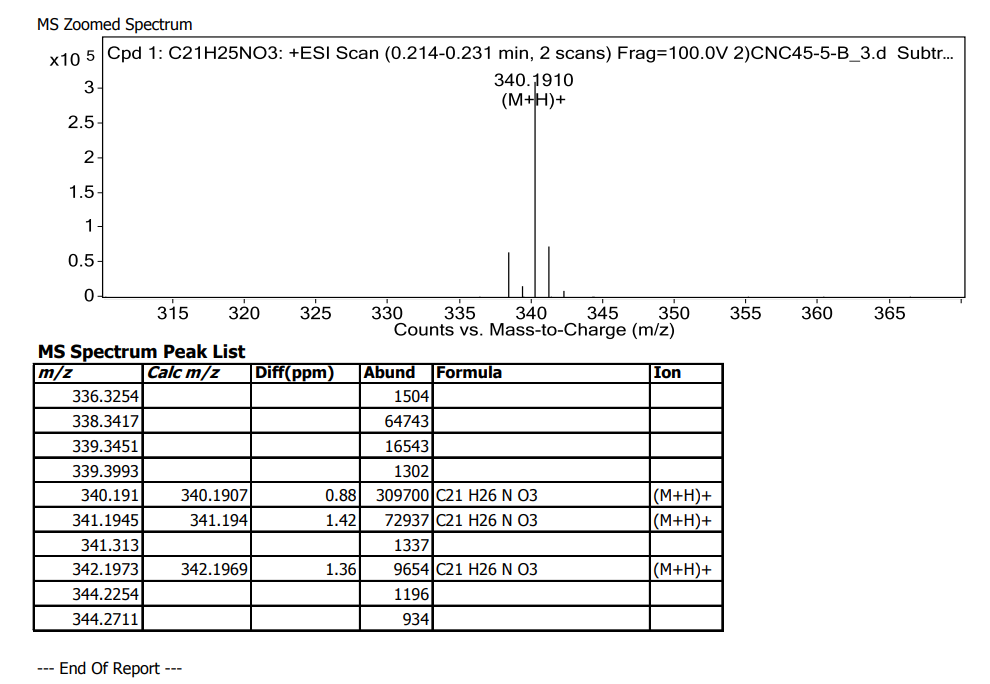


**Figure S19.** UV spectrum of compound **3** (in MeOH).


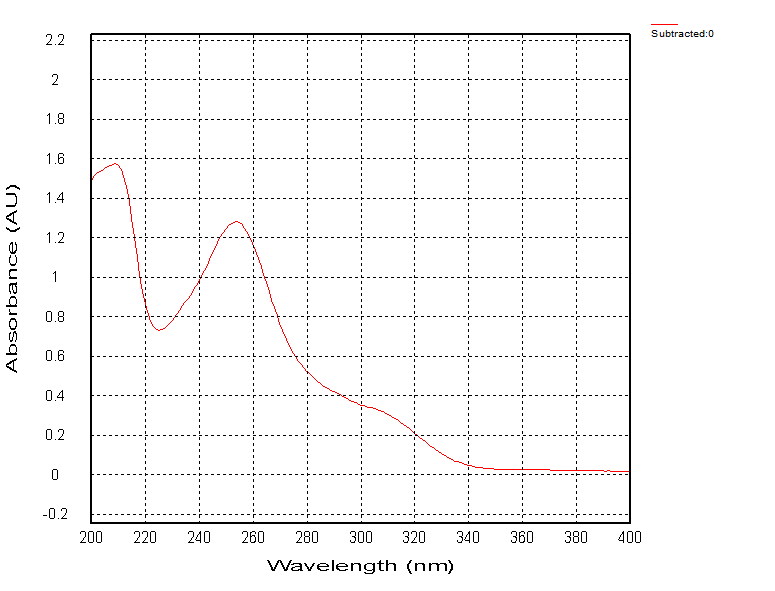


**Figure S20.** ^1^H NMR spectrum (800 MHz, CD_3_OD) of compound **3**.

**
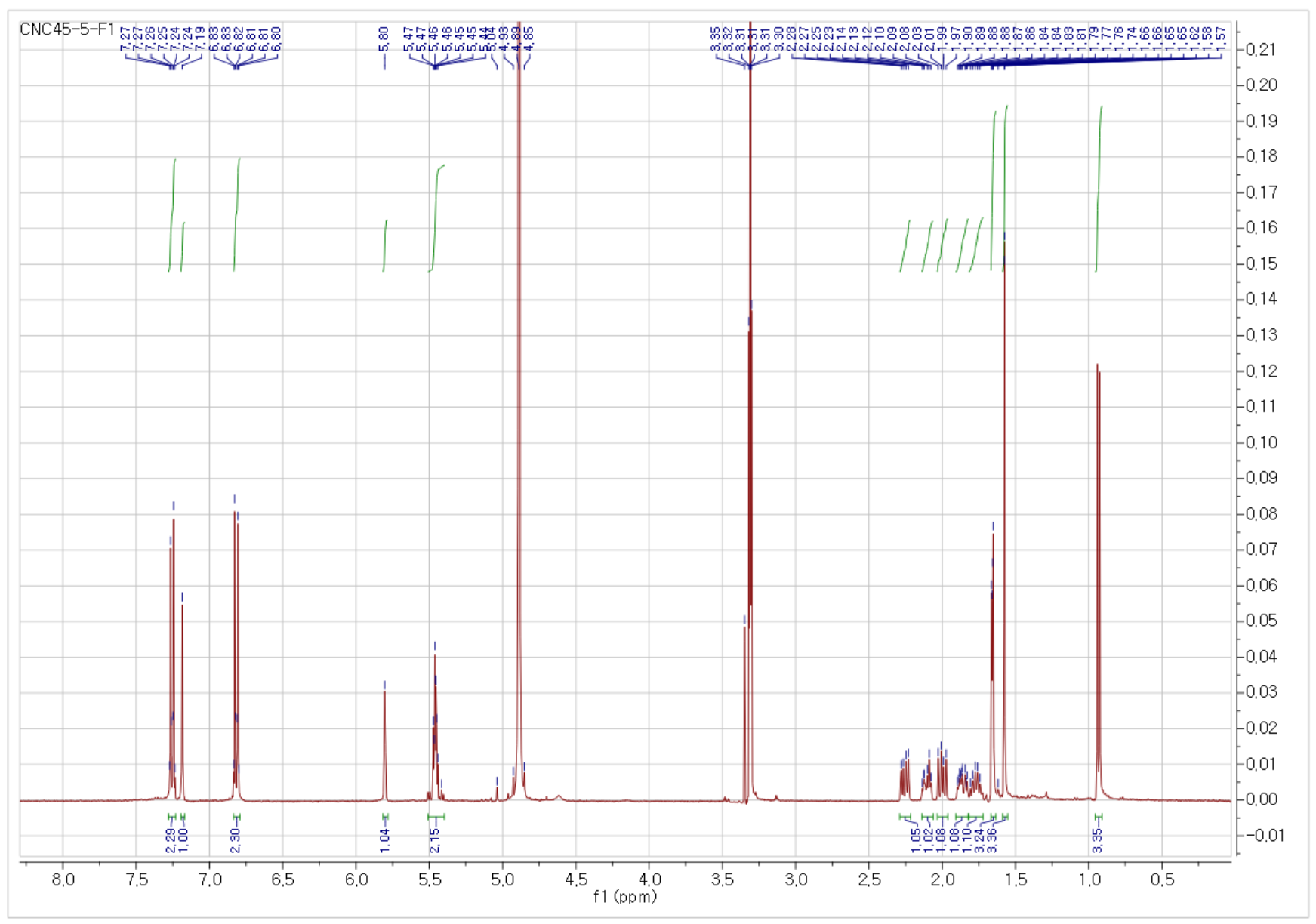
**

**Figure S21.** ^13^C NMR spectrum (200 MHz, CD_3_OD) of compound **3**.


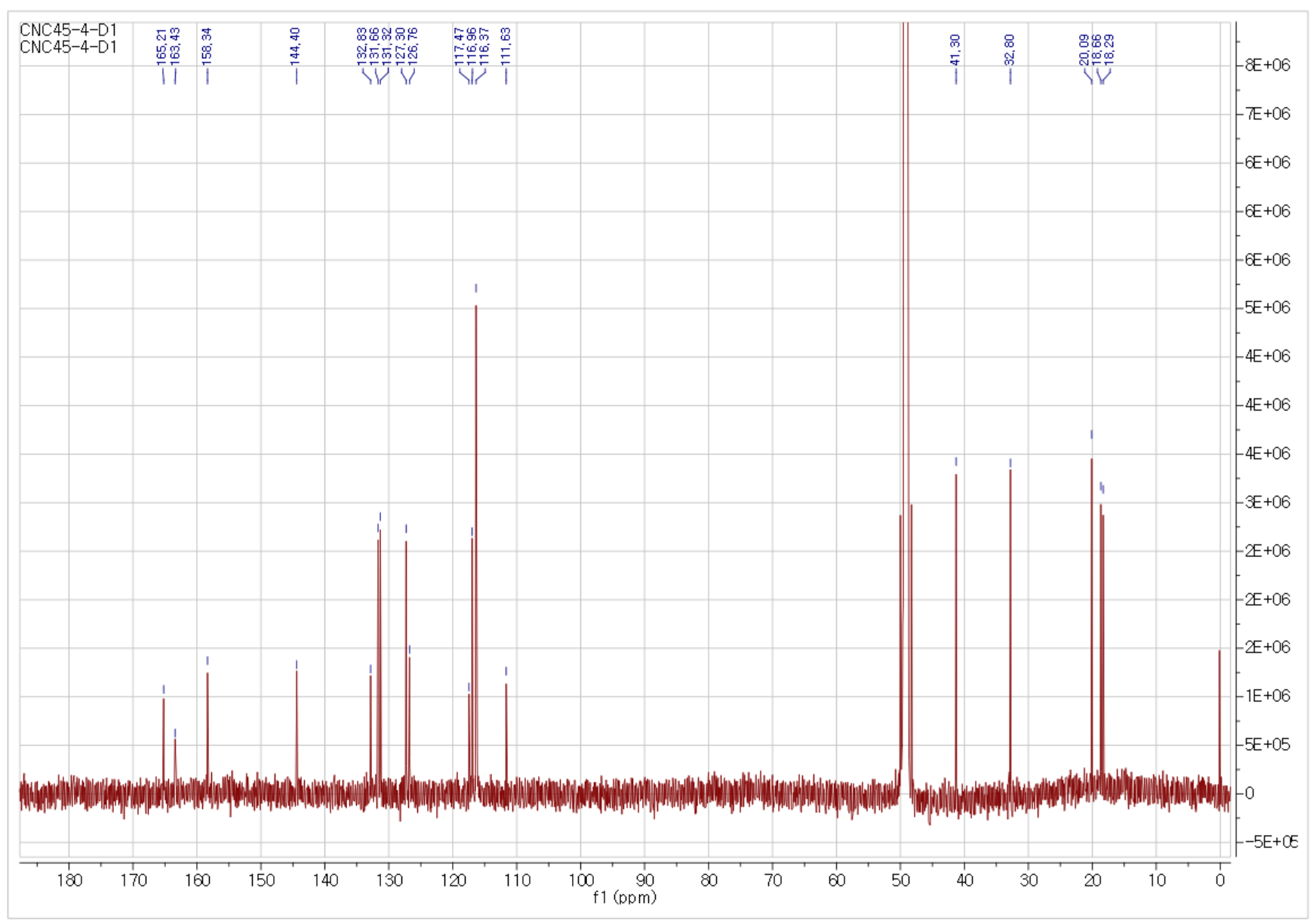


**Figure S22.** ^1^H-^1^H COSY spectrum (800 MHz, CD_3_OD) of compound **3**.


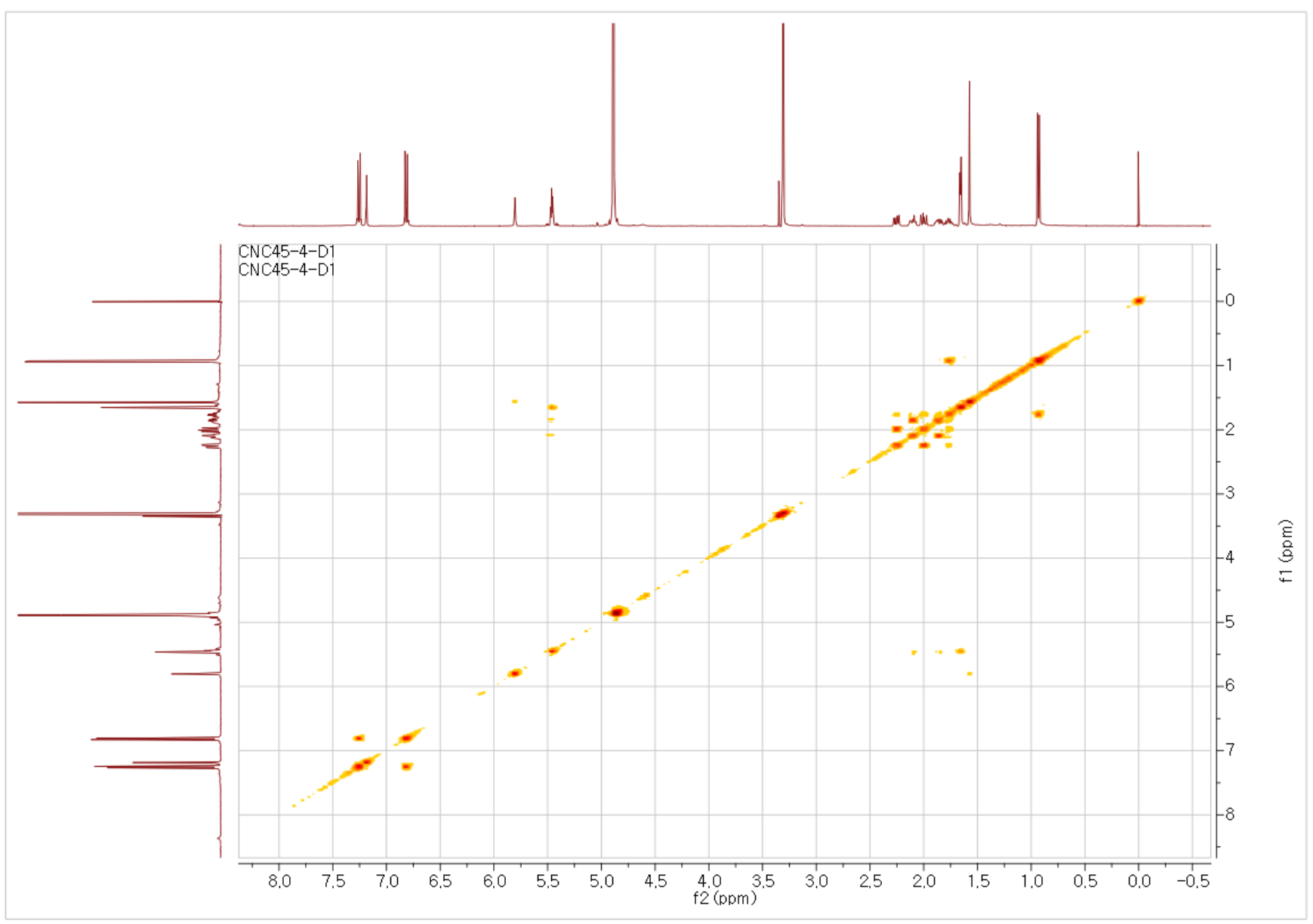


**Figure S23.** HSQC spectrum (800 MHz, CD_3_OD) of compound **3**.

**
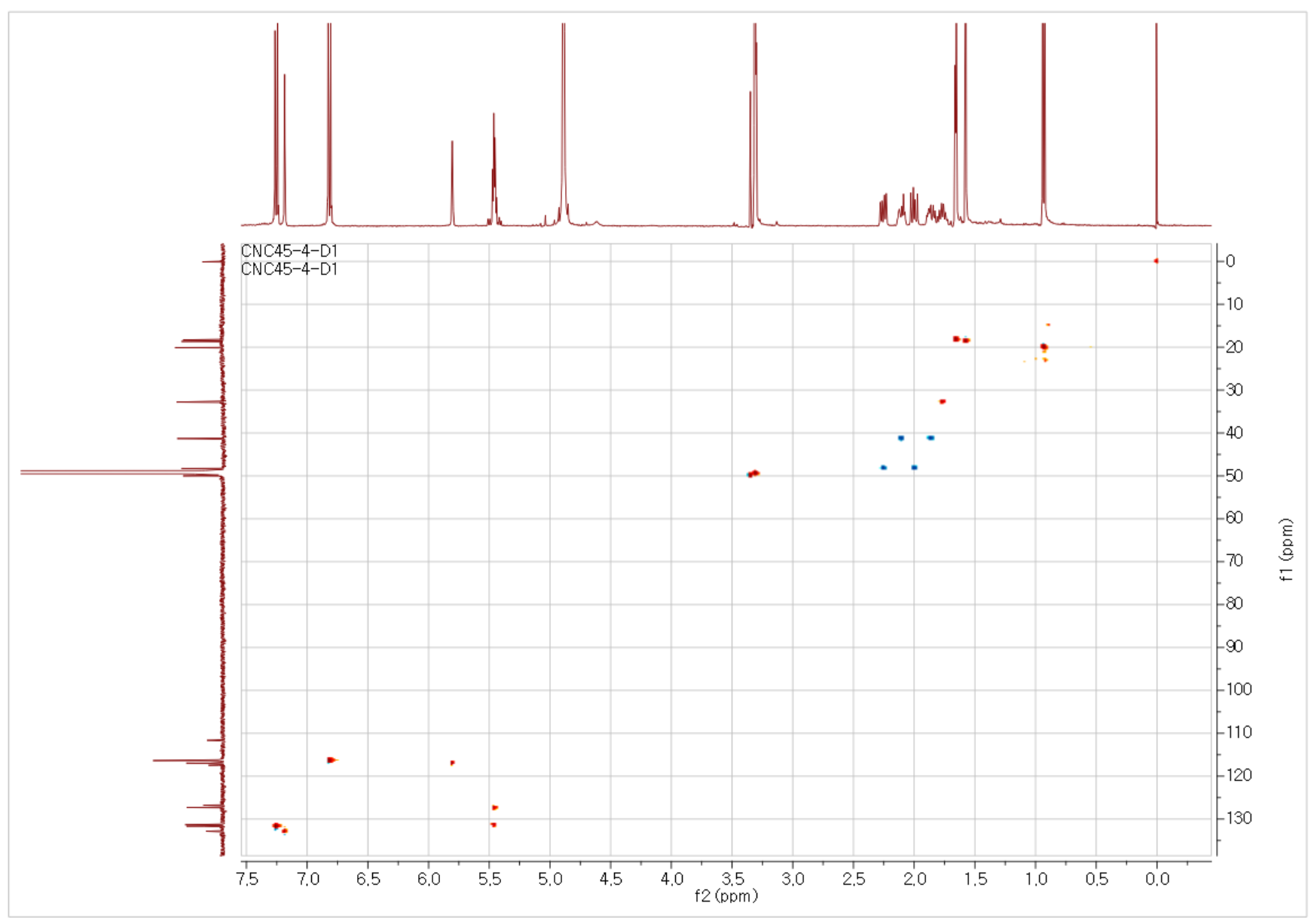
**

**Figure S24.** HMBC spectrum (800 MHz, CD_3_OD) of compound **3**.**
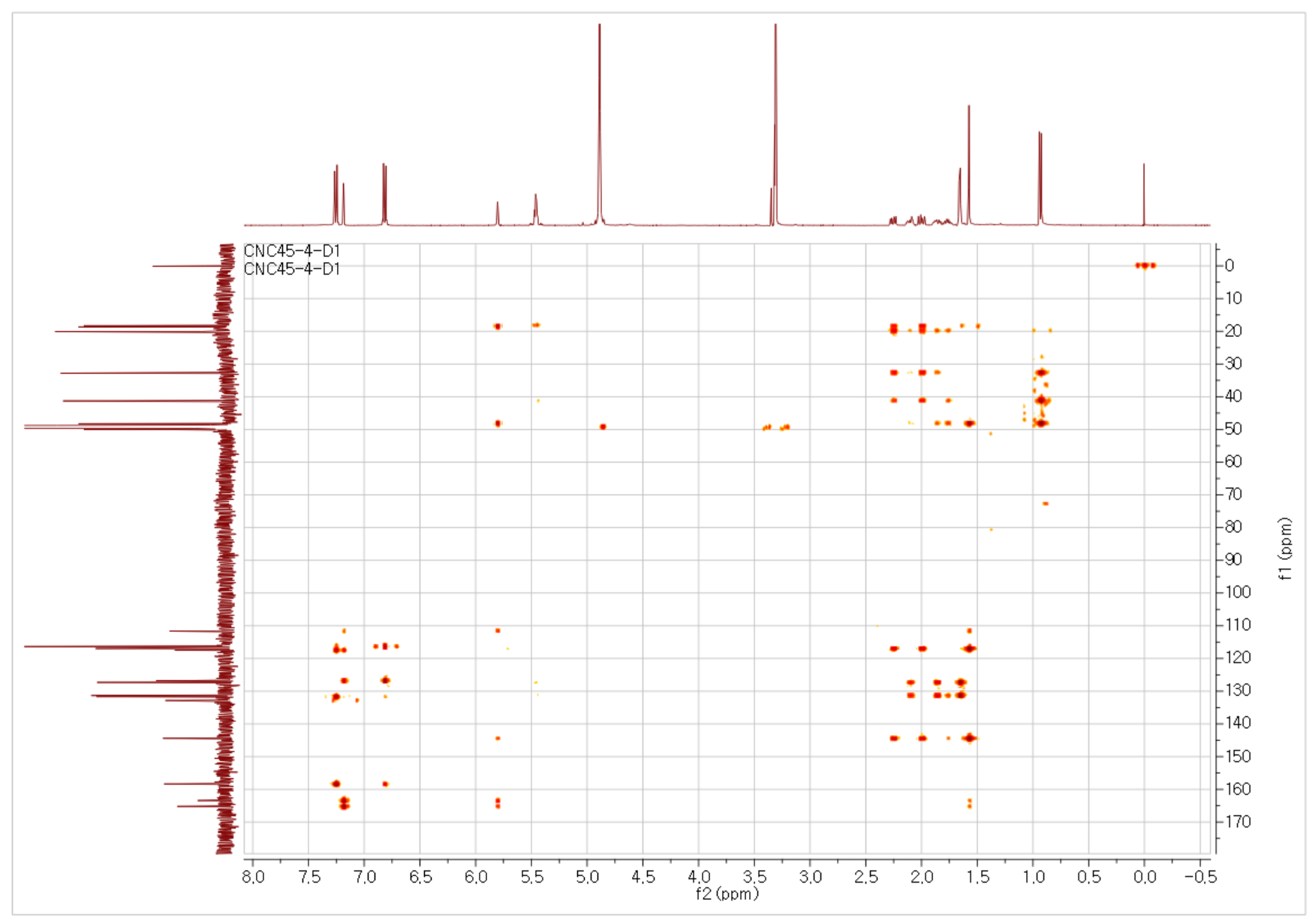
**

**Figure S25.** ROESY spectrum (800 MHz, CD_3_OD) of compound **3**.

**
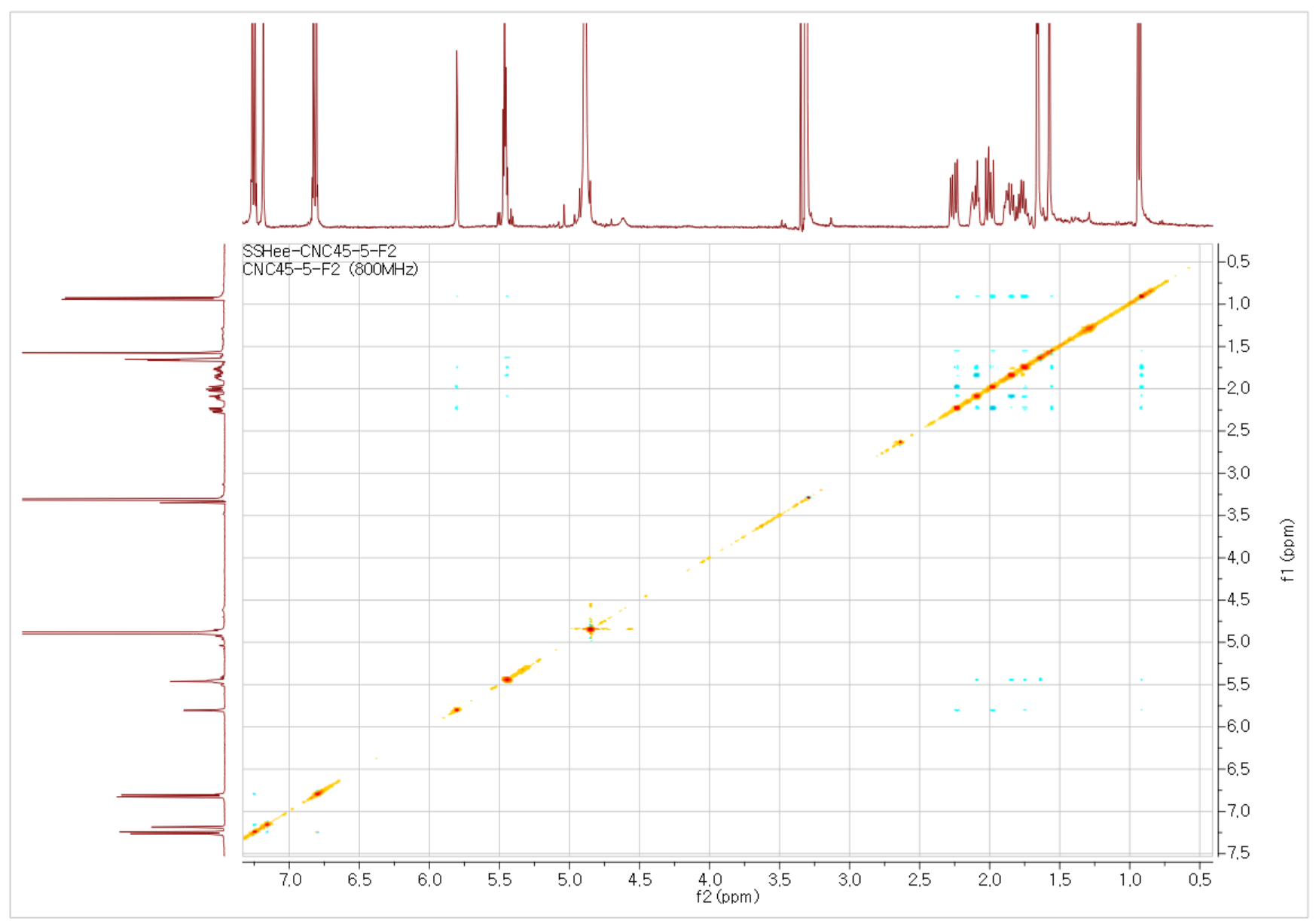
**

**Figure S26.** HRESIMS spectrum of compound **3**.


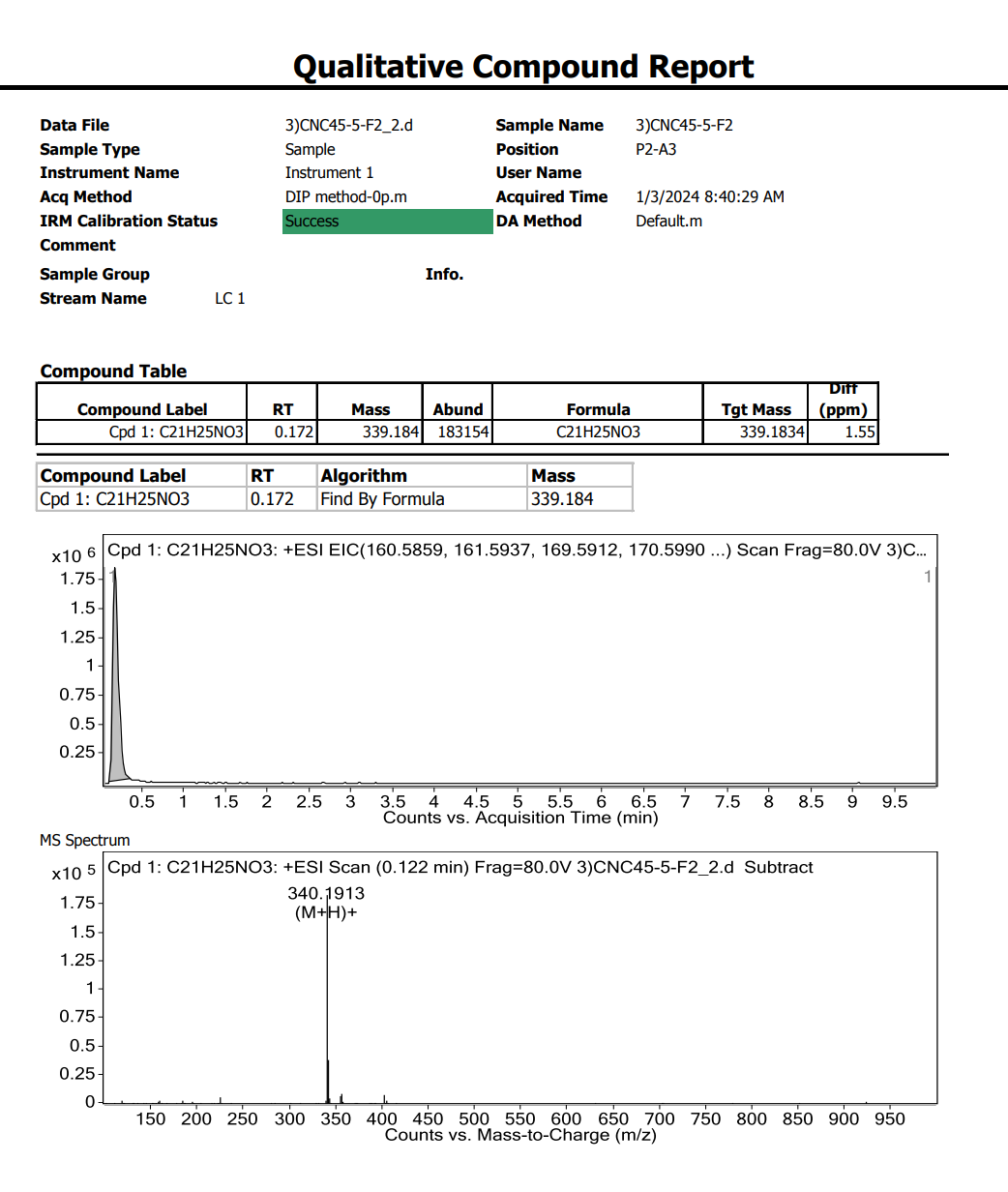


**
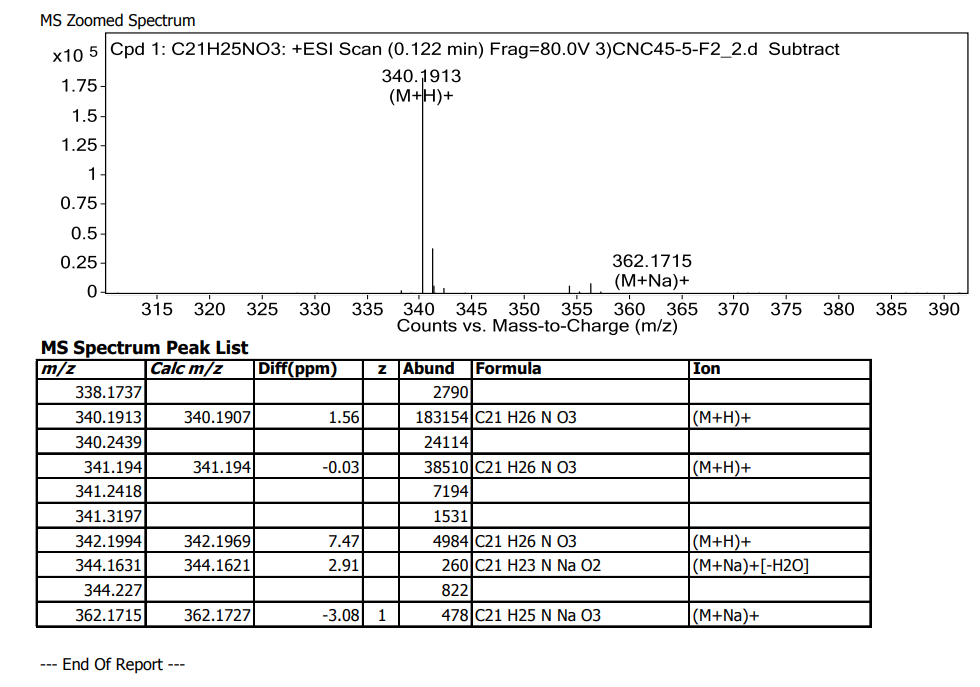
**

**Figure S27.** UV spectrum of compound **4** (in MeOH).


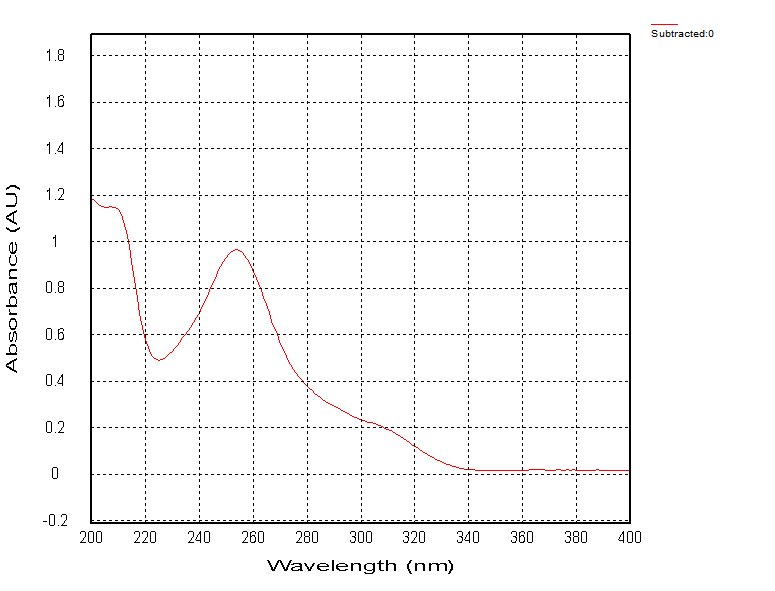


**Figure S28.** ^1^H NMR spectrum (800 MHz, CD_3_OD) of compound **4**.

**
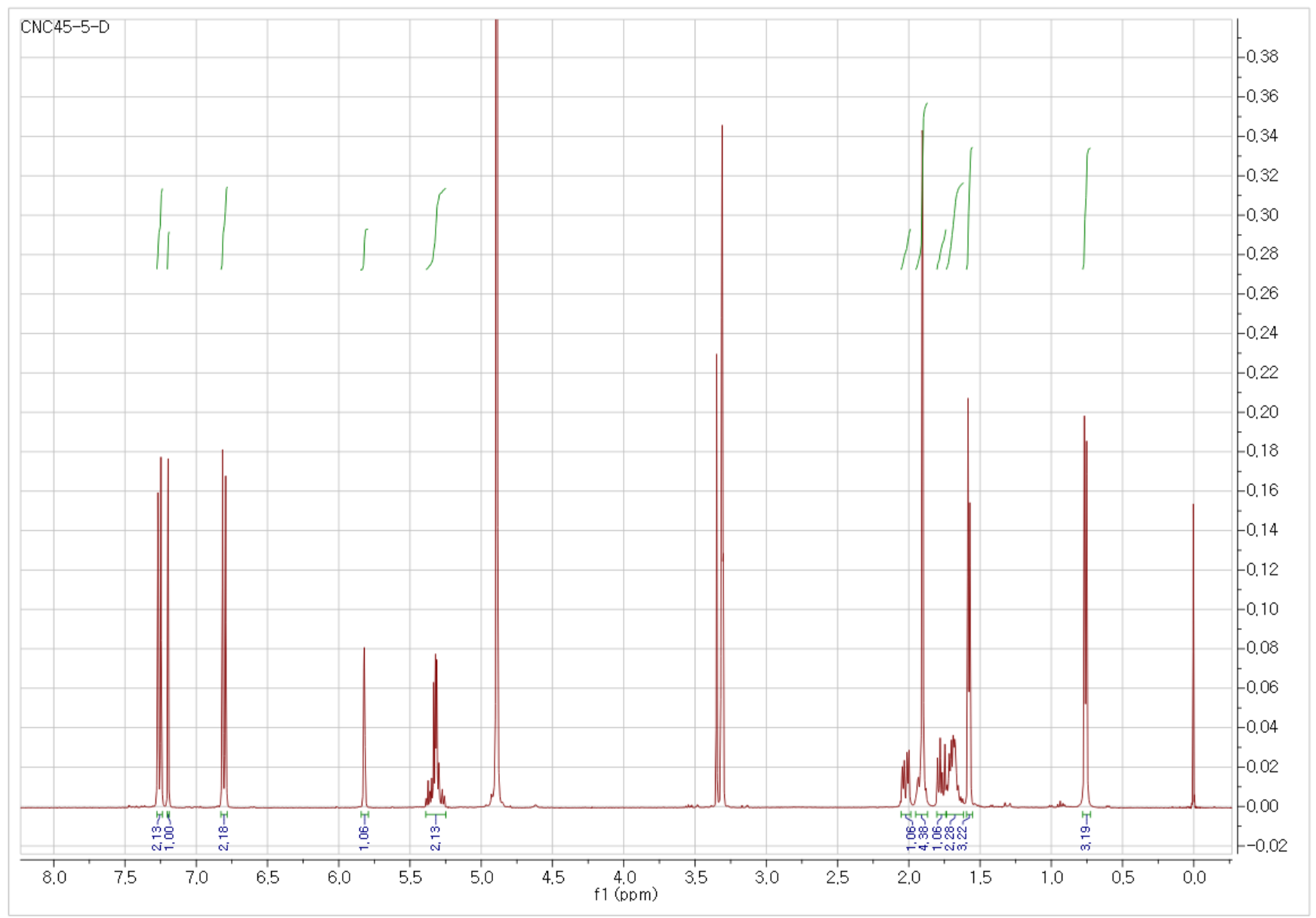
**

**Figure S29.** ^13^C NMR spectrum (200 MHz, CD_3_OD) of compound **4**.


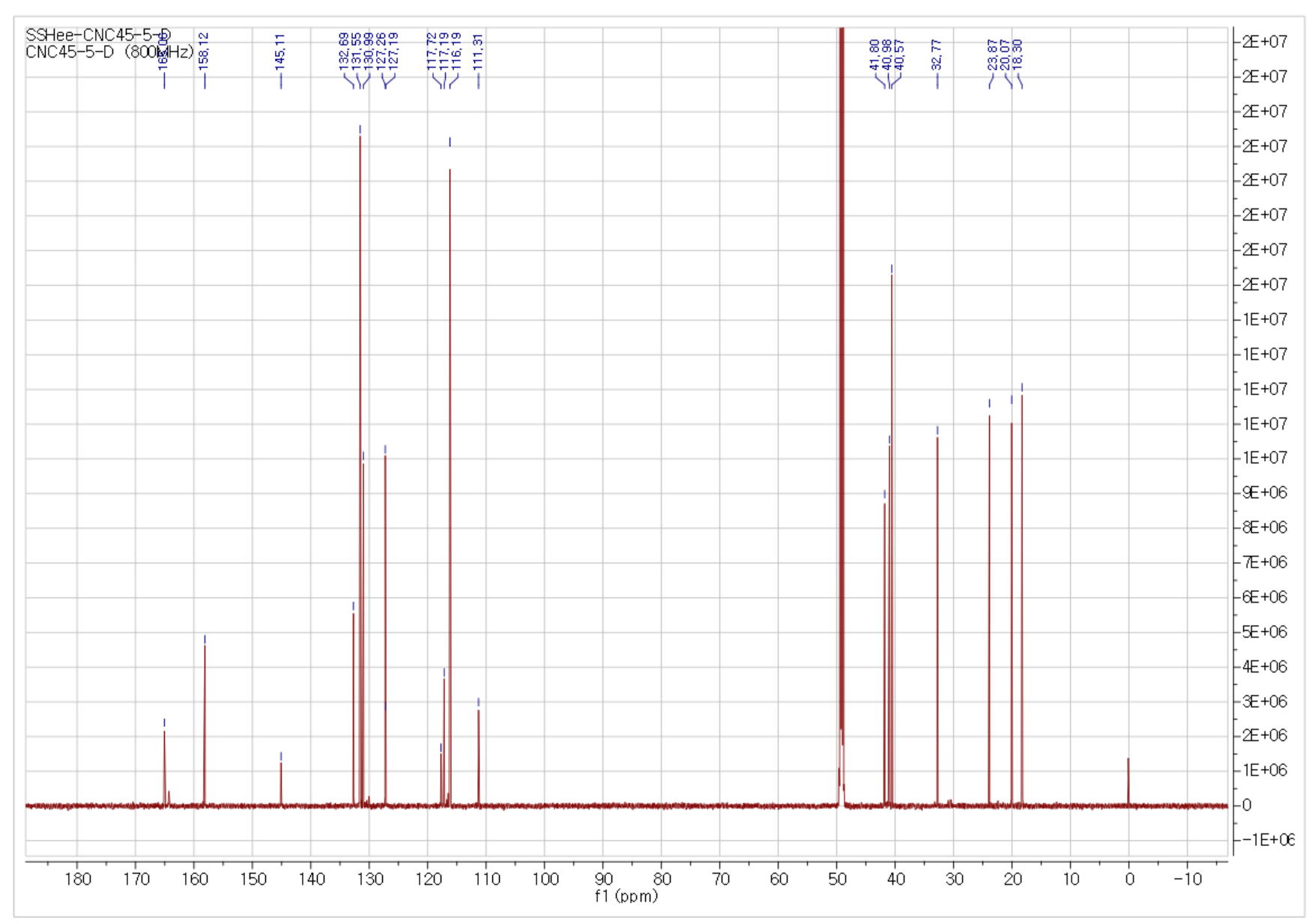


**Figure S30.** ^1^H-^1^H COSY spectrum (800 MHz, CD_3_OD) of compound **4**.


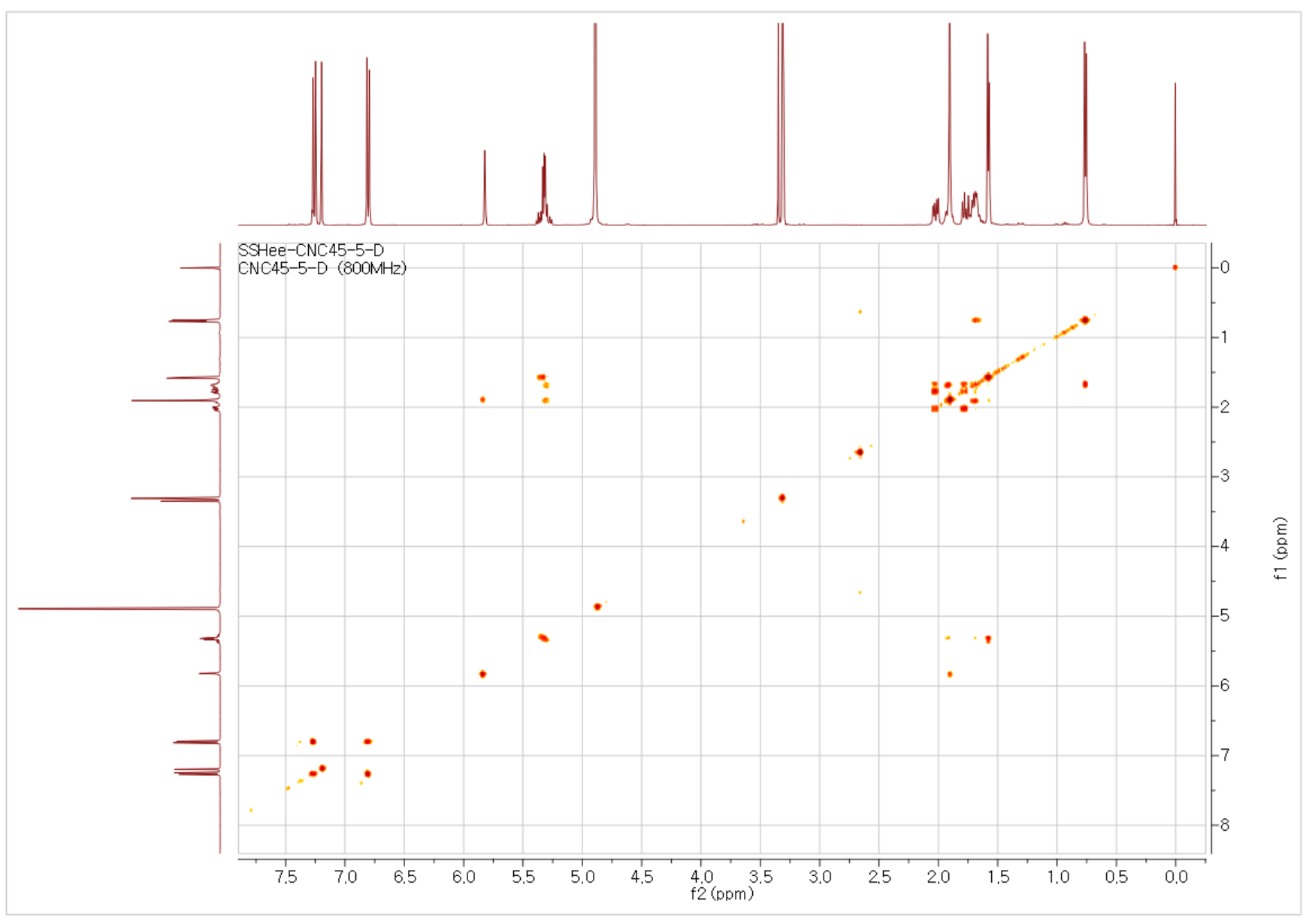


**Figure S31.** HSQC spectrum (800 MHz, CD_3_OD) of compound **4**.

**
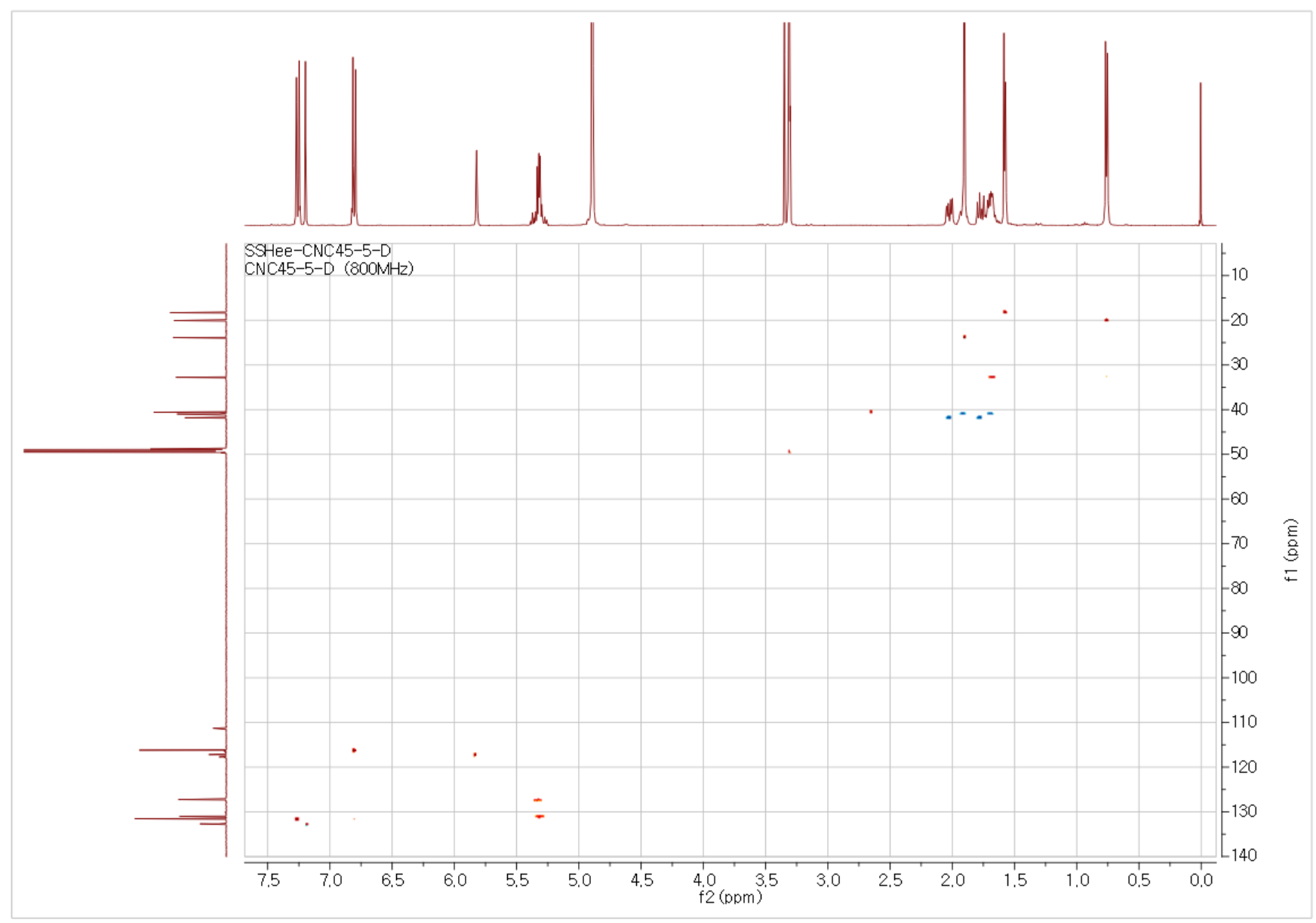
**

**Figure S32.** HMBC spectrum (800 MHz, CD_3_OD) of compound **4**.

**
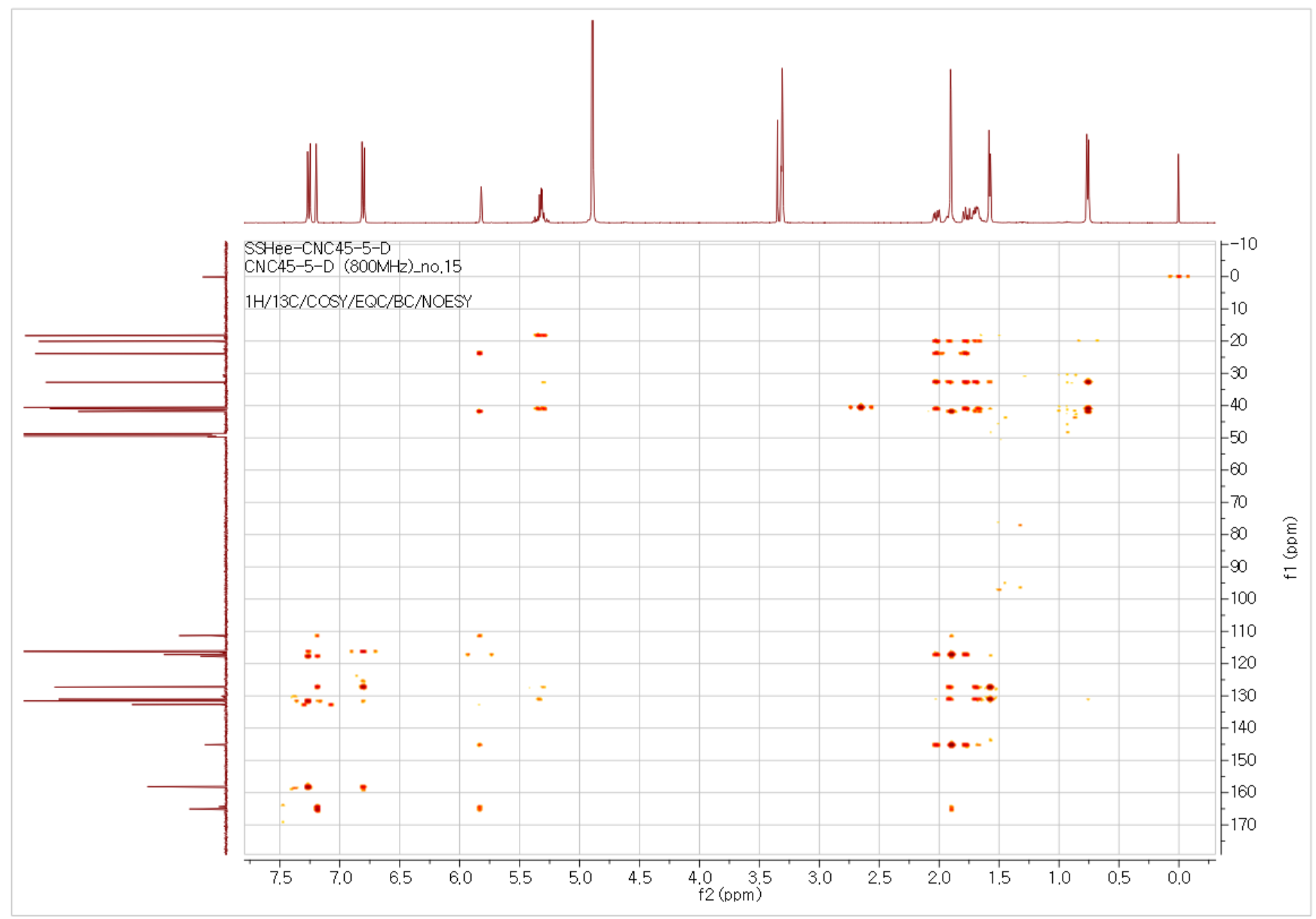
**

**Figure S33.** ROESY spectrum (800 MHz, CD_3_OD) of compound **4**.

**
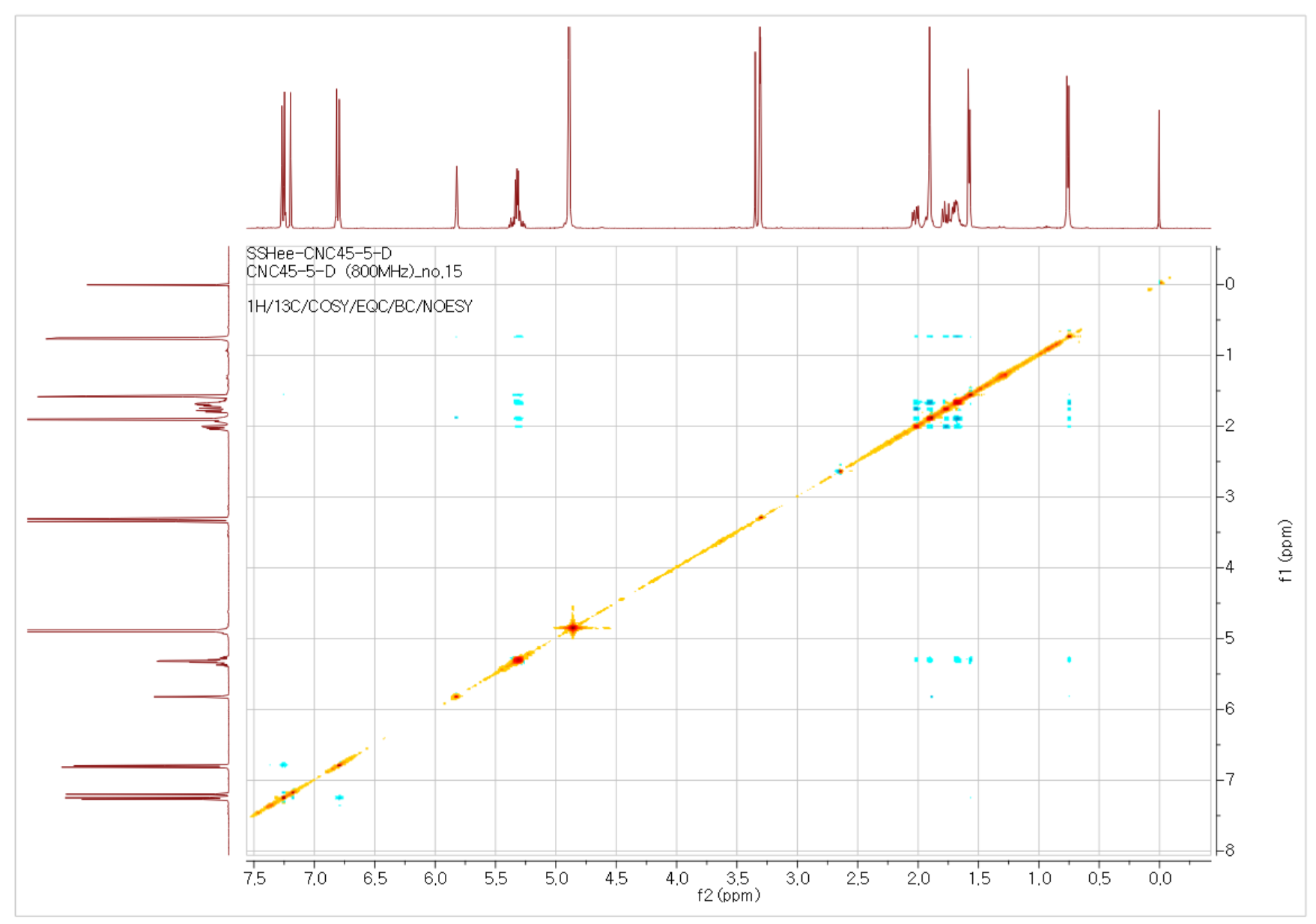
**

**Figure S34.** HRESIMS spectrum of compound **4**.


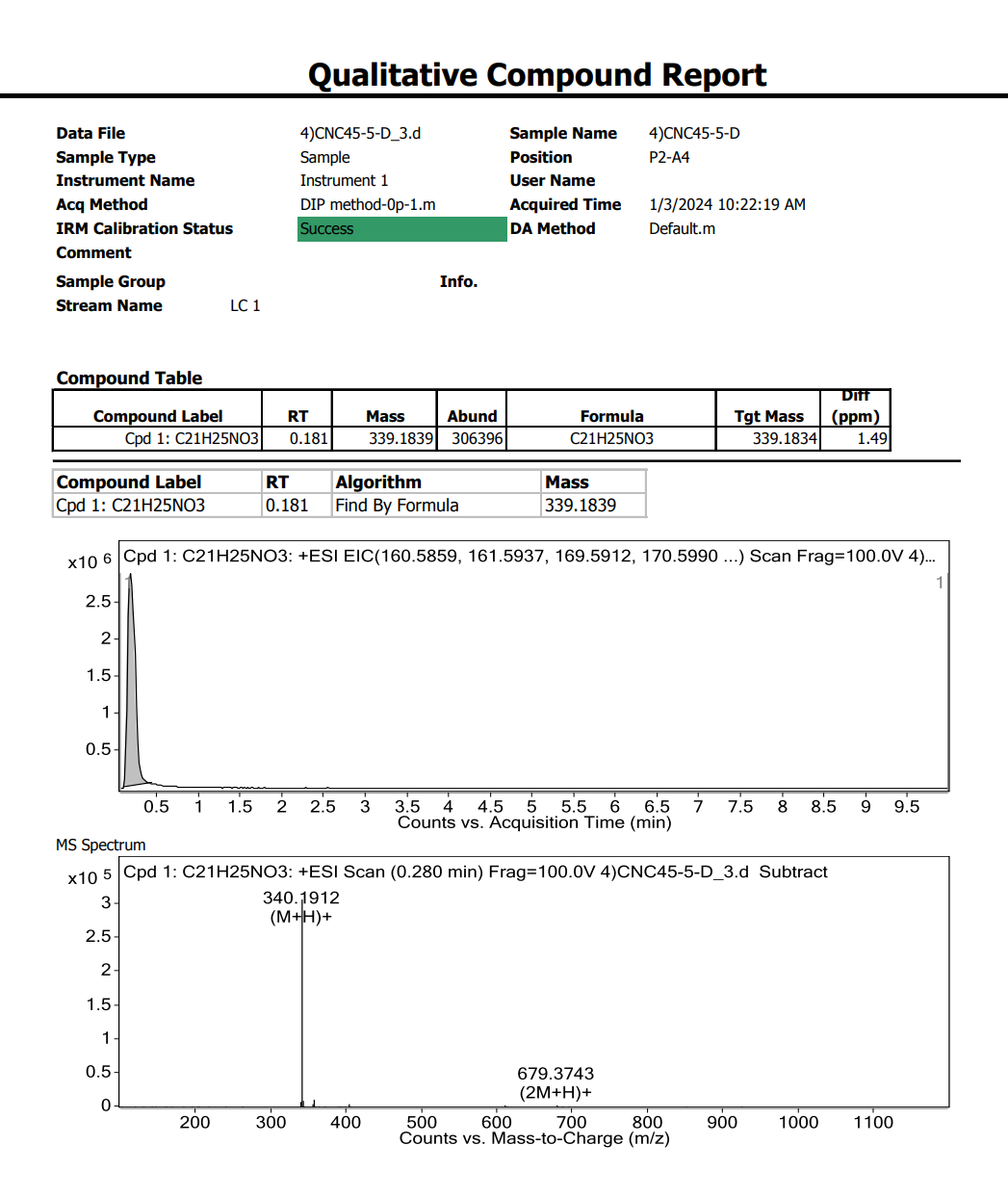

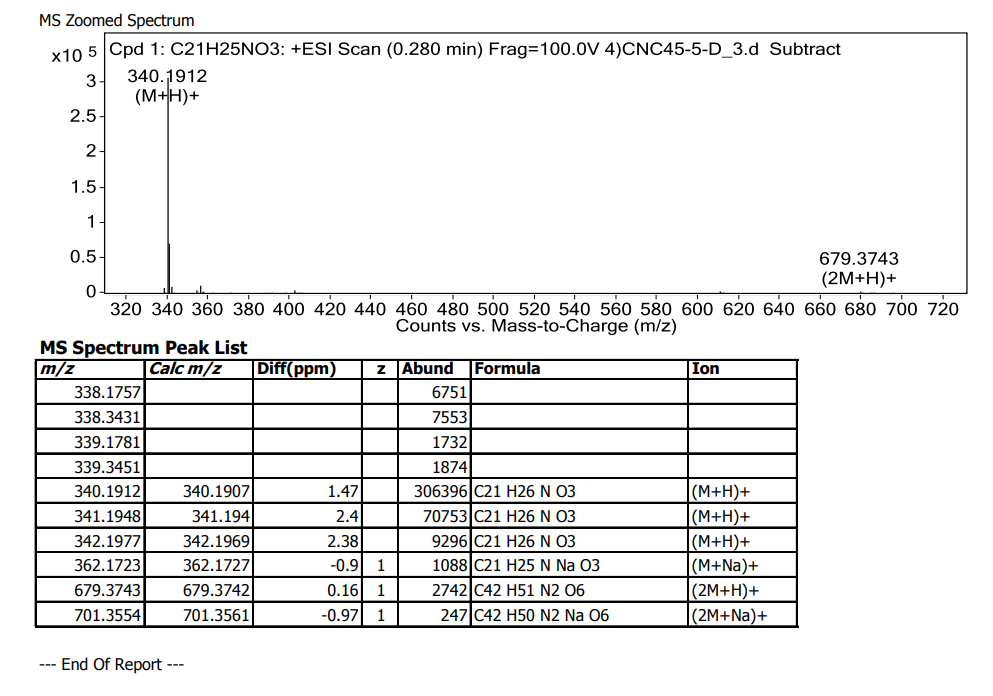


**Figure S35.** UV spectra comparison of compounds **1**, **2**, **5**, and **6** (in MeOH).


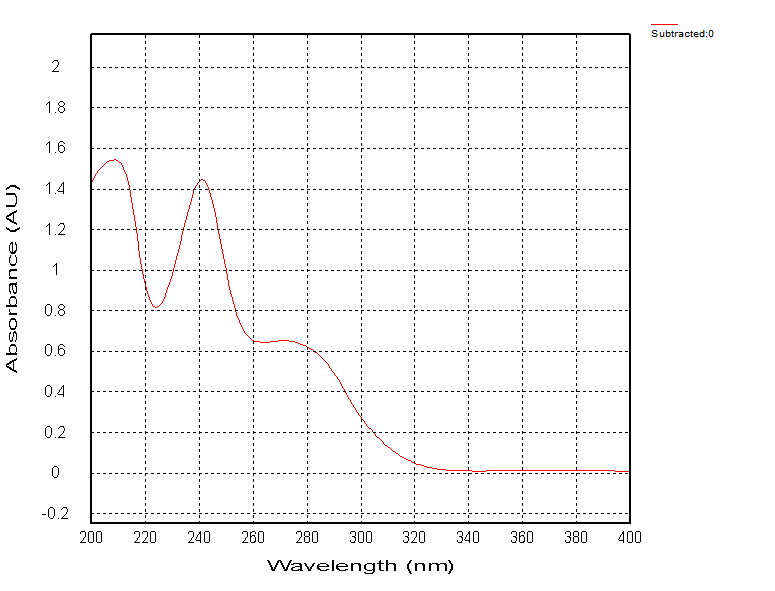

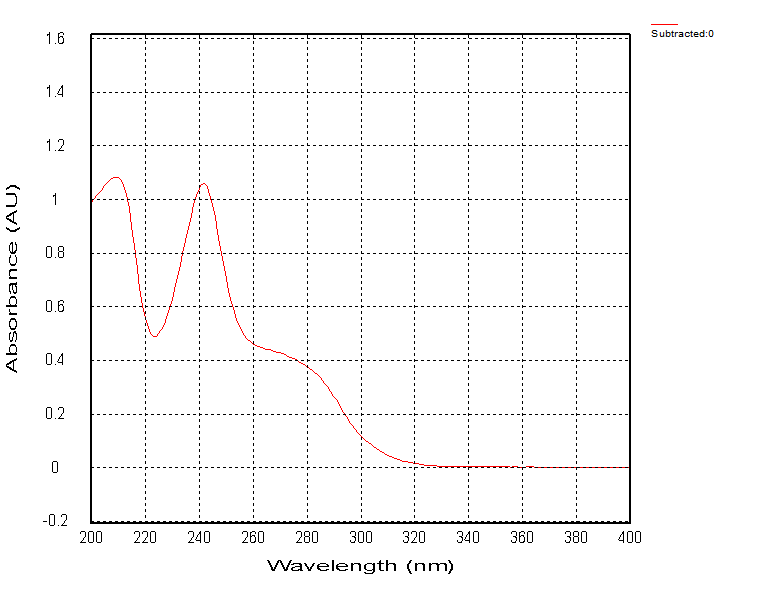


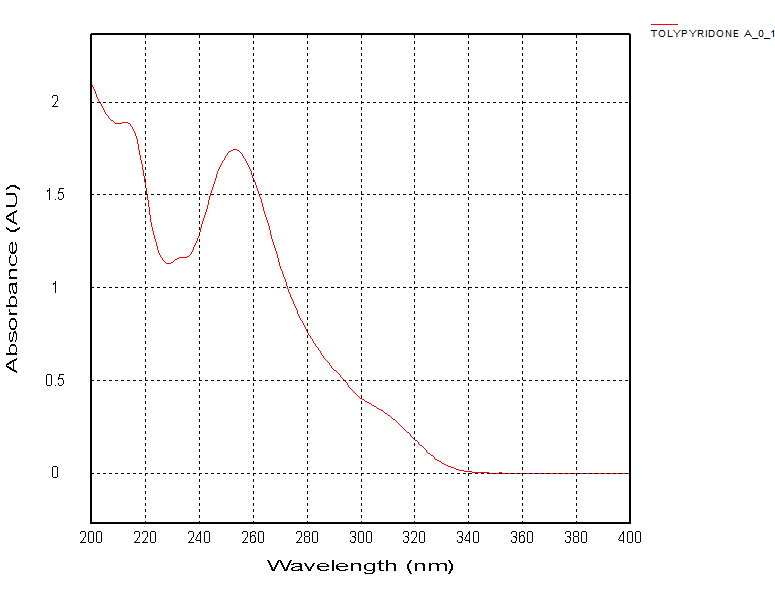

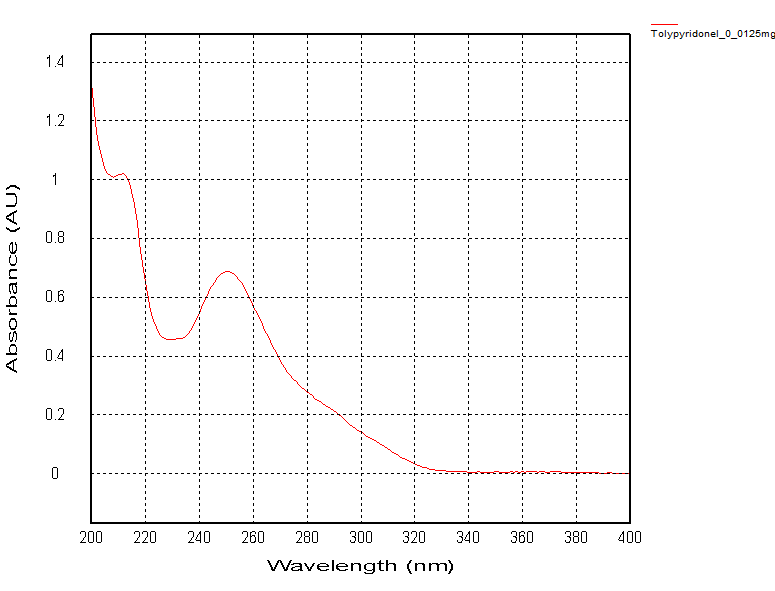


**Figure S36**. ^1^H-NMR spectra (800 MHz, DMSO-*d*_6_) of **1**, **2**, **5**, and **6**.


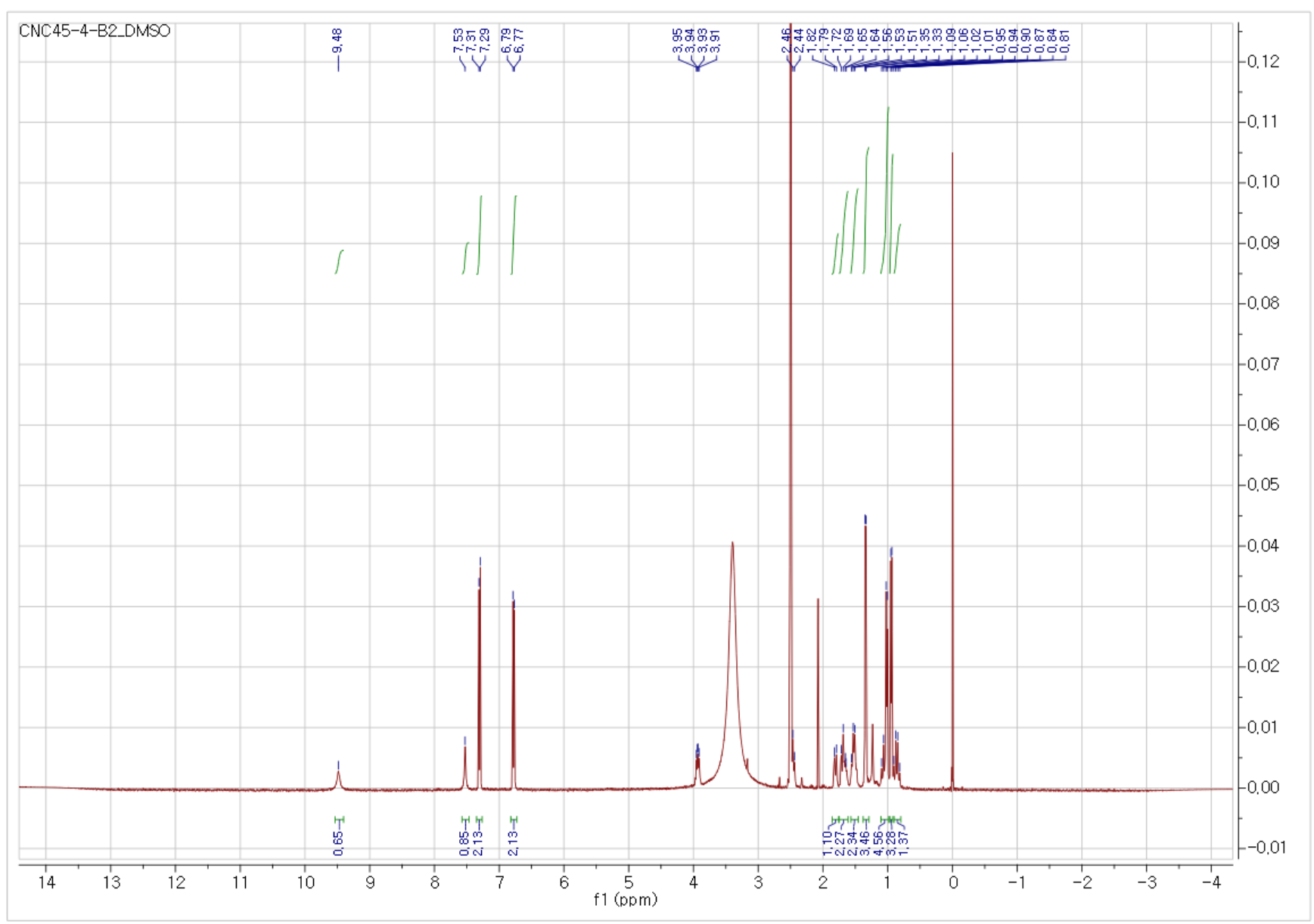

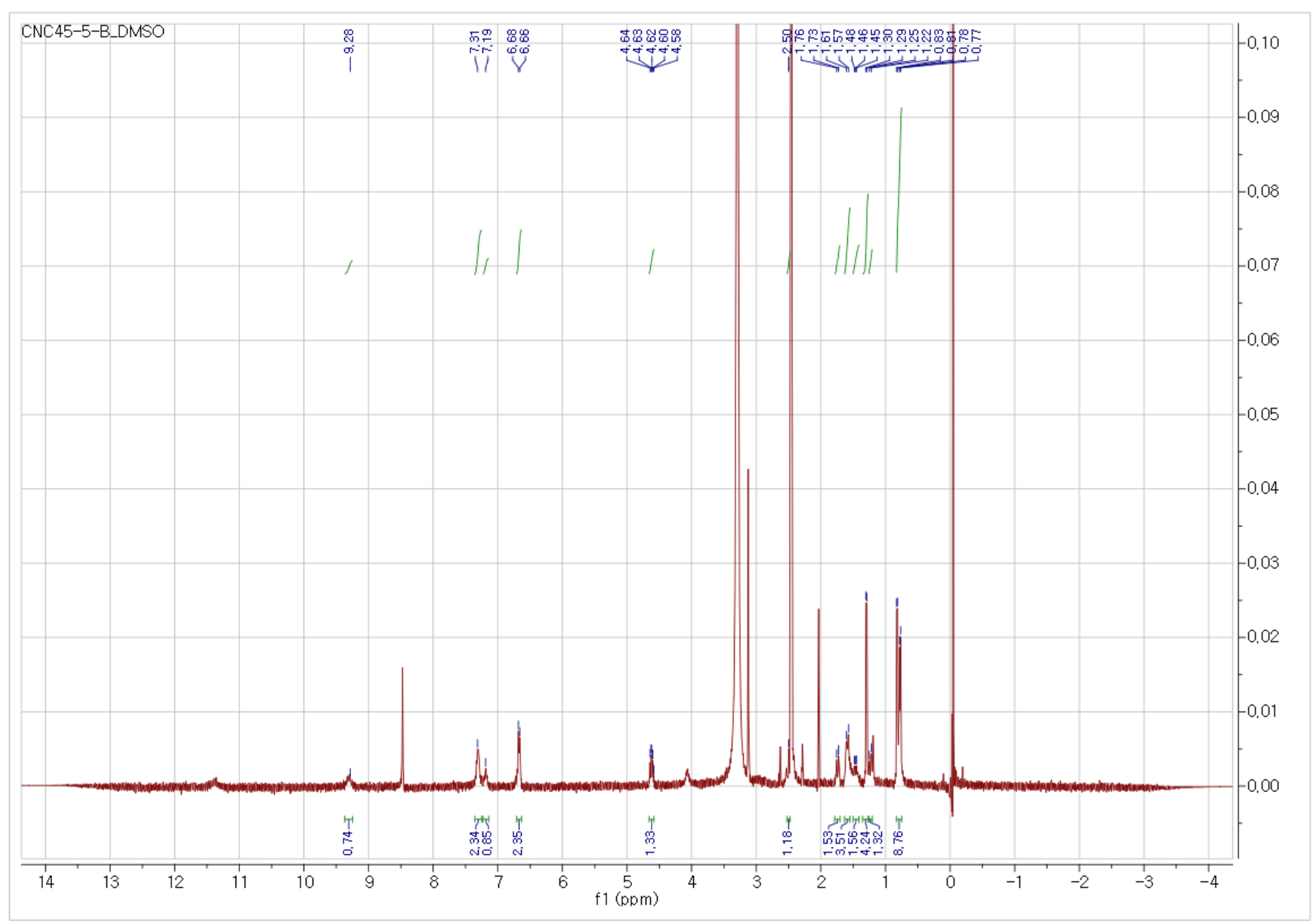

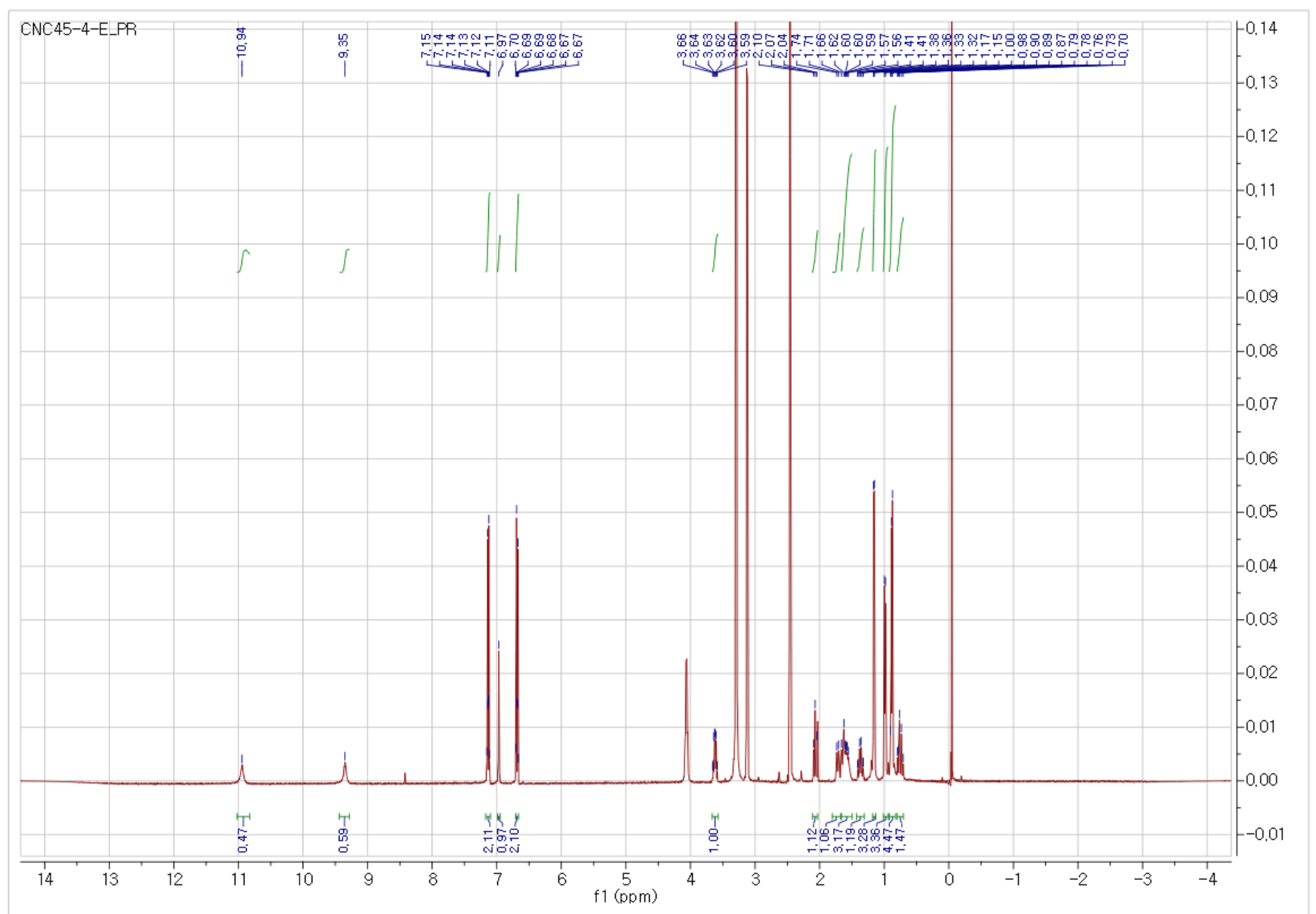

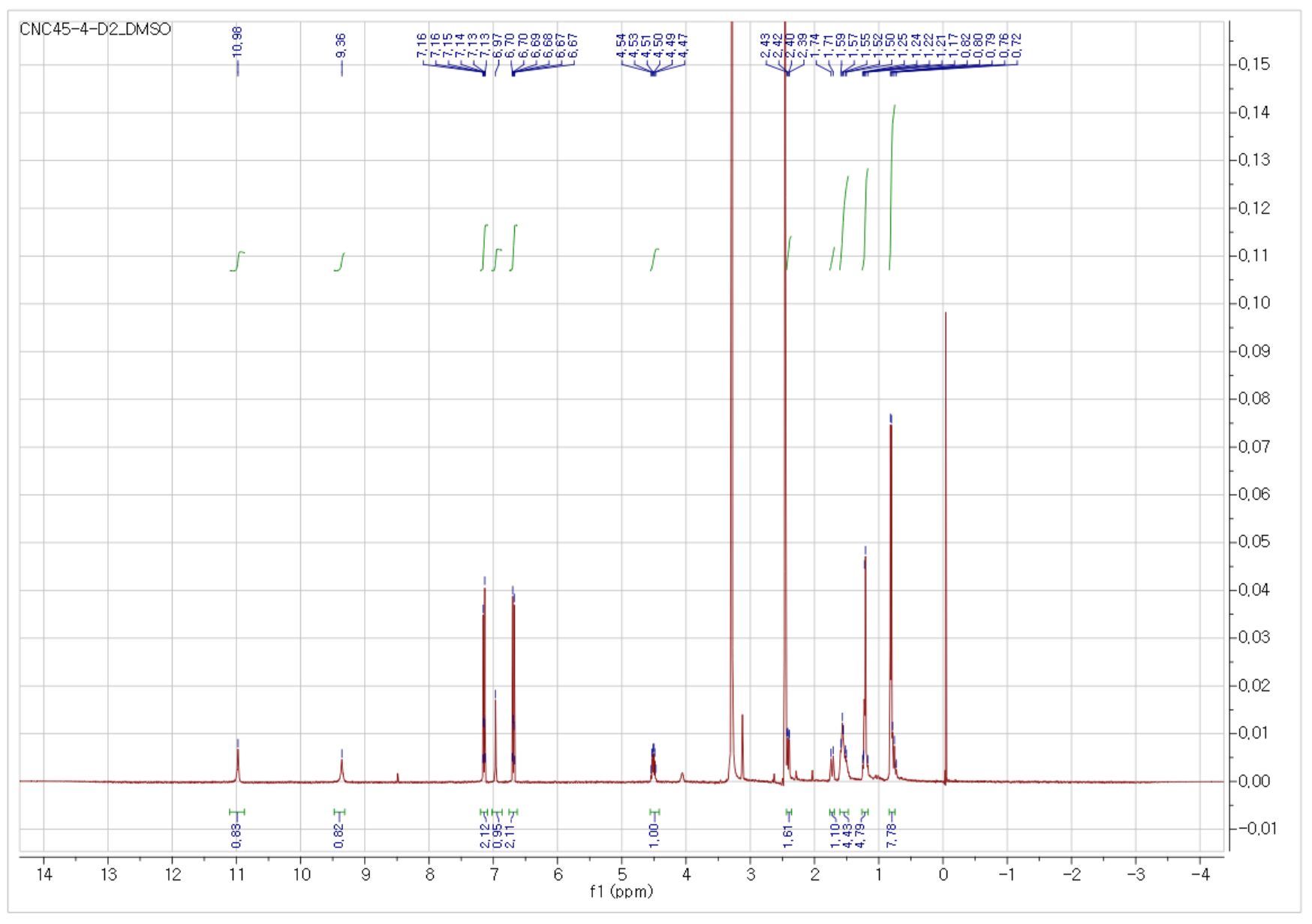


**Figure S37**. Conformer calculation & DP4+ calculation result of isomers **1** and **2**.

**
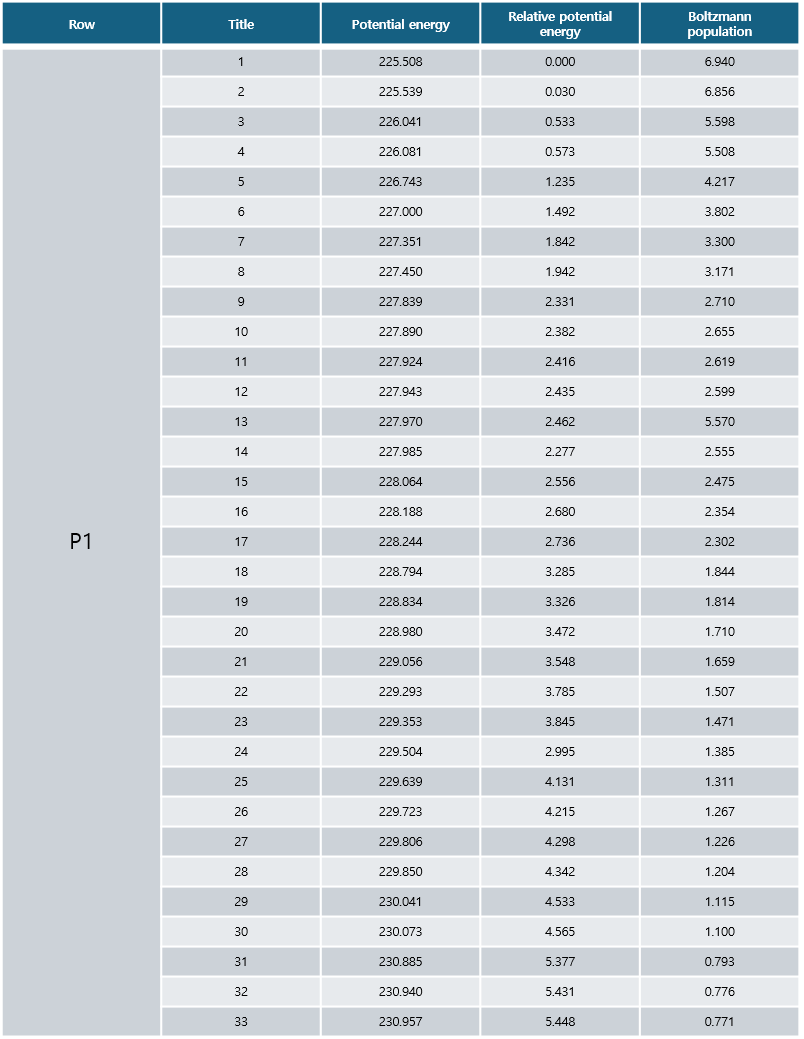

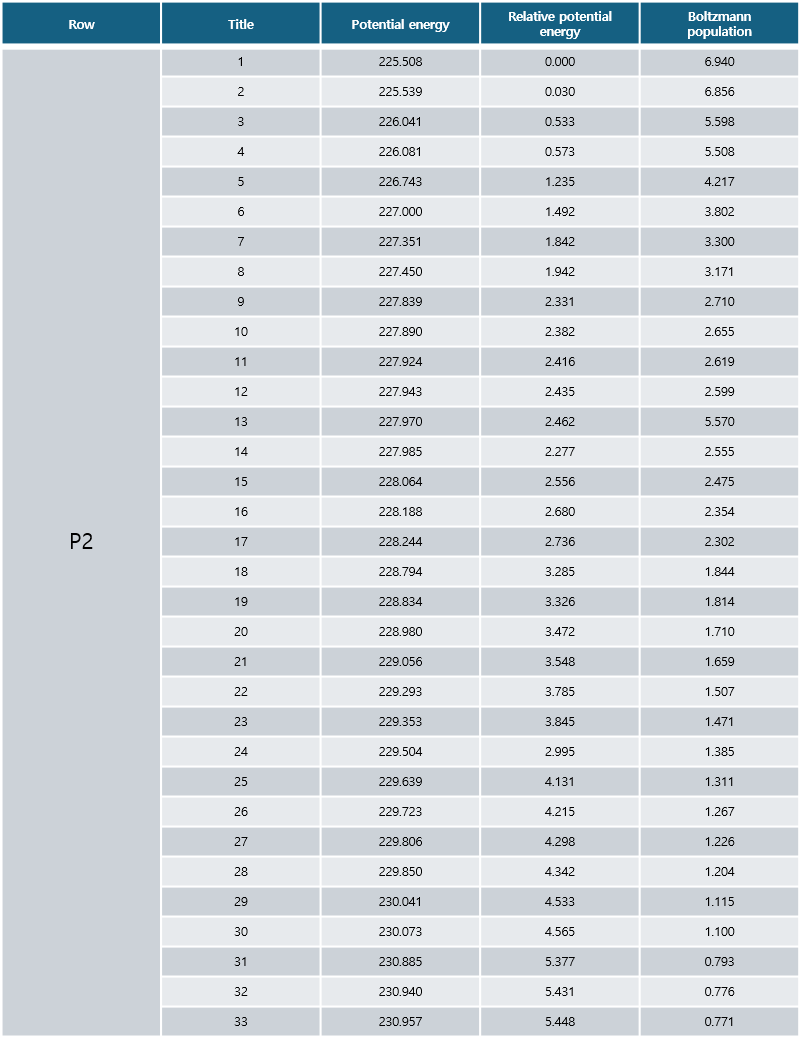
**

**
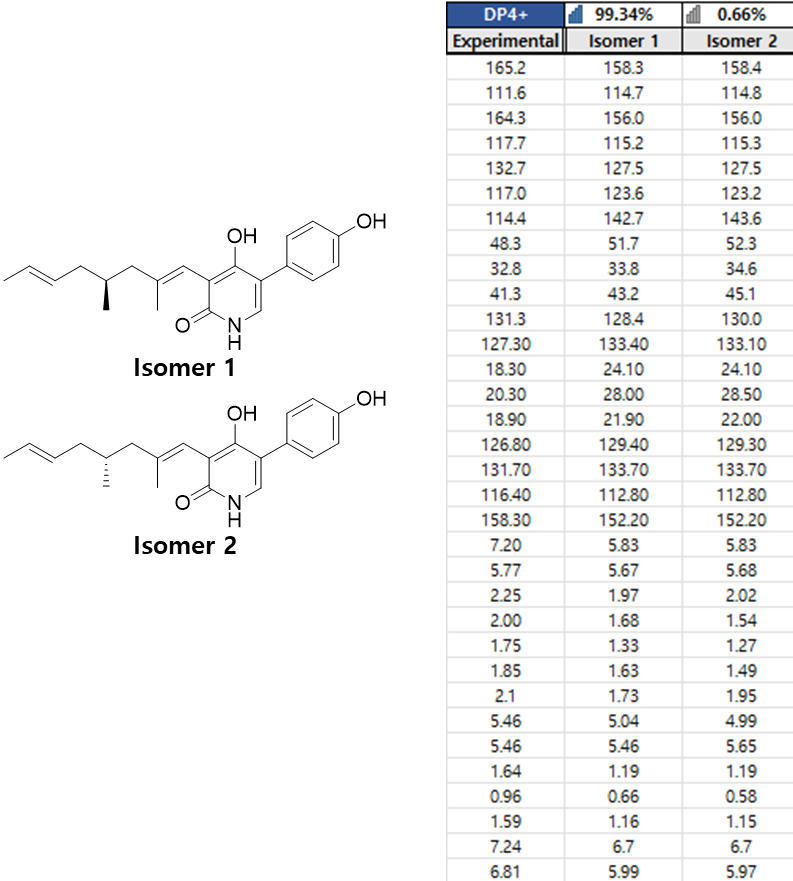
**

**Table S1.** Coordinates and energies of the low-energy conformers of **1** and **2** calculated with def2-TZVPP atomic attributes in vacuo.

| Isomers | | **P1** | | | **P2** | | | **P3** | | | **P4** | | |
| --- | --- | --- | --- | --- | --- | --- | --- | --- | --- | --- | --- | --- | --- |
| No | Atom | Standard Oreientation(Angstroms) | | | Standard Oreientation(Angstroms) | | | Standard Oreientation(Angstroms) | | | Standard Oreientation(Angstroms) | | |
|  |  | X | Y | Z | X | Y | Z | X | Y | Z | X | Y | Z |
| 1 | C | -9.4937 | 1.4952 | -2.768 | 6.2073 | 1.7702 | -0.4208 | -5.1763 | -0.3586 | -0.7657 | -6.071 | -0.5071 | 0.4615 |
| 2 | C | -8.2762 | 2.095 | -3.0631 | 5.9082 | 1.3995 | -1.7255 | -4.8879 | -1.7106 | -0.9104 | -6.0409 | -1.9005 | 0.5451 |
| 3 | C | -7.0891 | 1.4988 | -2.6335 | 4.5918 | 1.09 | -2.0724 | -3.6963 | -2.1766 | -1.3392 | -4.9155 | -2.6416 | 0.484 |
| 4 | C | -7.1124 | 0.2961 | -1.9083 | 3.5658 | 1.1463 | -1.1146 | -2.746 | -1.2696 | -1.651 | -3.7442 | -1.9806 | 0.3935 |
| 5 | C | -8.3565 | -0.2895 | -1.6203 | 3.8934 | 1.5265 | 0.1976 | -2.9171 | 0.1079 | -1.5904 | -3.6172 | -0.5973 | 0.4028 |
| 6 | C | -9.546 | 0.3068 | -2.0488 | 5.2108 | 1.8377 | 0.5462 | -4.1746 | 0.5462 | -1.1517 | -4.8184 | 0.1293 | 0.4703 |
| 7 | O | -10.627 | 2.1113 | -3.2071 | 7.5051 | 2.0631 | -0.1264 | -1.8241 | 1.011 | -1.9655 | -2.2894 | 0.0255 | 0.3478 |
| 8 | C | -5.8649 | -0.3362 | -1.4678 | 2.1853 | 0.8095 | -1.4763 | -1.4007 | 1.1126 | -3.3014 | -1.8943 | 0.7742 | -0.7734 |
| 9 | C | -5.5244 | -0.3576 | -0.1181 | 1.2112 | 1.802 | -1.5378 | -0.366 | 1.9807 | -3.662 | -0.6357 | 1.3766 | -0.8216 |
| 10 | N | -4.4075 | -0.9249 | 0.3861 | -0.086 | 1.5906 | -1.8479 | 0.2511 | 2.7514 | -2.6834 | 0.2379 | 1.2325 | 0.2484 |
| 11 | C | -3.5605 | -1.4895 | -0.4962 | -0.444 | 0.3243 | -2.1363 | -0.1474 | 2.6671 | -1.3556 | -0.1252 | 0.4944 | 1.369 |
| 12 | C | -3.7469 | -1.4918 | -1.8747 | 0.4346 | -0.7536 | -2.1681 | -1.1803 | 1.7989 | -0.9969 | -1.3862 | -0.1072 | 1.4161 |
| 13 | C | -4.9459 | -0.9359 | -2.3425 | 1.7584 | -0.4903 | -1.7887 | 1.2625 | 3.6119 | -2.9886 | 1.4555 | 1.8375 | 0.1591 |
| 14 | O | -5.1956 | -1.0183 | -3.6905 | 2.6181 | -1.5588 | -1.716 | -5.7954 | -2.7084 | -0.719 | -7.1601 | -2.6656 | 0.734 |
| 15 | O | -2.4873 | -2.1111 | 0.0857 | -1.7849 | 0.1666 | -2.3683 | -6.5257 | 0.0963 | -0.2602 | -7.3967 | 0.2463 | 0.4948 |
| 16 | C | -2.1328 | -3.2902 | -0.6415 | -2.2397 | -1.0903 | -1.86 | -7.5618 | -0.9794 | -0.6624 | -8.5984 | -0.7363 | 0.4178 |
| 17 | C | -1.6066 | -2.9022 | -2.0341 | -1.6027 | -2.2364 | -2.6646 | -7.0681 | -2.3559 | -0.1671 | -8.2533 | -1.9438 | 1.3047 |
| 18 | C | -2.7033 | -2.0935 | -2.7973 | -0.0469 | -2.135 | -2.5709 | -6.5563 | 0.3759 | 1.2748 | -7.554 | 1.4158 | -0.5222 |
| 19 | C | -0.2628 | -2.158 | -1.9932 | -2.091 | -2.3053 | -4.12 | -7.9855 | 0.7158 | 1.7414 | -7.9681 | 0.9593 | -1.9278 |
| 20 | C | 0.2903 | -1.9036 | -3.403 | -1.533 | -3.5356 | -4.8501 | -9.012 | -0.3472 | 1.3393 | -9.1452 | -0.0116 | -1.9982 |
| 21 | C | -0.8102 | -1.6328 | -4.4388 | -0.1112 | -3.9042 | -4.403 | -8.9625 | -0.5874 | -0.1716 | -8.9405 | -1.1708 | -1.0257 |
| 22 | C | -2.1185 | -1.0911 | -3.8441 | 0.6892 | -2.7338 | -3.8127 | -4.4842 | 1.8848 | -1.1147 | -4.7933 | 1.4948 | 0.6219 |
| 23 | C | -1.1343 | -4.0722 | 0.2065 | -3.7649 | -1.0769 | -1.8947 | -8.0148 | -3.4938 | -0.5487 | -9.4125 | -2.9222 | 1.4704 |
| 24 | C | 1.3489 | -0.8 | -3.378 | -1.638 | -3.3592 | -6.3657 | -6.8085 | 1.0192 | -0.7859 | -7.4177 | 0.6847 | 1.505 |
| 25 | C | -1.9439 | 0.3527 | -3.3488 | 1.0735 | -1.722 | -4.903 | -7.5979 | -1.0158 | -1.762 | -9.4784 | -0.2351 | 0.8404 |
| 26 | H | -3.2469 | -2.8594 | -3.3735 | 0.2115 | -2.7819 | -1.7172 | -5.6253 | 1.512 | 1.7151 | -8.4795 | 2.5029 | 0.0414 |
| 27 | H | -1.43 | -3.8344 | -2.591 | -1.9057 | -3.1808 | -2.1889 | -10.4172 | 0.0737 | 1.7699 | -10.5108 | 0.6527 | -1.8257 |
| 28 | H | -8.2503 | 3.0268 | -3.6217 | 6.6962 | 1.3559 | -2.4725 | -1.8019 | -1.7047 | -1.9705 | -2.8708 | -2.6252 | 0.3271 |
| 29 | H | -6.1413 | 1.9836 | -2.8637 | 4.3688 | 0.8091 | -3.1008 | -1.8828 | 0.5166 | -4.075 | -2.5664 | 0.8878 | -1.623 |
| 30 | H | -8.4056 | -1.2232 | -1.0618 | 3.1193 | 1.5767 | 0.9621 | -0.0609 | 2.0401 | -4.7021 | -0.337 | 1.9521 | -1.6936 |
| 31 | H | -10.4932 | -0.1685 | -1.8136 | 5.4357 | 2.1268 | 1.5682 | 0.3471 | 3.2717 | -0.6001 | 0.5512 | 0.3766 | 2.2099 |
| 32 | H | -11.3974 | 1.6001 | -2.9096 | 7.563 | 2.329 | 0.806 | -1.4776 | 1.7358 | 0.049 | -1.6647 | -0.6785 | 2.3006 |
| 33 | H | -6.1703 | 0.0974 | 0.6296 | 1.4557 | 2.8406 | -1.3265 | 1.4513 | 3.5493 | -3.9393 | 1.9669 | 1.6328 | 0.9591 |
| 34 | H | -6.0872 | -0.6755 | -3.8754 | 3.4749 | -1.2606 | -1.3645 | -6.9599 | -2.377 | 0.9227 | -7.9715 | -1.606 | 2.3126 |
| 35 | H | -3.0204 | -3.9322 | -0.7512 | -1.9517 | -1.1817 | -0.8014 | -6.2266 | -0.5202 | 1.8157 | -6.6001 | 1.9289 | -0.6702 |
| 36 | H | 0.486 | -2.746 | -1.4499 | -3.1846 | -2.3726 | -4.1558 | -7.9957 | 0.8309 | 2.8329 | -7.0996 | 0.4558 | -2.376 |
| 37 | H | -0.3676 | -1.2224 | -1.433 | -1.8341 | -1.3801 | -4.6474 | -8.2816 | 1.6876 | 1.3231 | -8.1616 | 1.8306 | -2.566 |
| 38 | H | 0.8077 | -2.8177 | -3.7269 | -2.1736 | -4.3919 | -4.5958 | -8.7743 | -1.2817 | 1.862 | -9.1484 | -0.4319 | -3.0138 |
| 39 | H | -1.0441 | -2.5823 | -4.9407 | -0.1903 | -4.6759 | -3.6242 | -9.699 | -1.3456 | -0.4592 | -8.1395 | -1.8033 | -1.4284 |
| 40 | H | -0.4459 | -0.9684 | -5.2321 | 0.4466 | -4.3756 | -5.2216 | -9.2665 | 0.3313 | -0.6921 | -9.8421 | -1.7947 | -1.0239 |
| 41 | H | -2.8309 | -1.0321 | -4.6785 | 1.638 | -3.161 | -3.4598 | -3.7557 | 2.3955 | -1.5086 | -3.8742 | 1.7951 | 0.729 |
| 42 | H | -0.7457 | -4.9411 | -0.3327 | -4.18 | -2.0478 | -1.6088 | -7.5718 | -4.4623 | -0.2899 | -9.1397 | -3.7046 | 2.1876 |
| 43 | H | -1.6216 | -4.4254 | 1.1225 | -4.1456 | -0.3213 | -1.198 | -8.9764 | -3.4168 | -0.0344 | -10.3114 | -2.4176 | 1.8368 |
| 44 | H | -0.2991 | -3.4452 | 0.5349 | -4.1479 | -0.7944 | -2.8806 | -8.1912 | -3.5089 | -1.6298 | -9.6464 | -3.4397 | 0.5346 |
| 45 | H | 1.8019 | -0.6745 | -4.3671 | -1.3164 | -4.2681 | -6.8852 | -5.743 | 1.7151 | 2.7855 | -8.6918 | 3.2703 | -0.7101 |
| 46 | H | 2.1506 | -1.0508 | -2.675 | -2.6739 | -3.1578 | -6.6594 | -4.5744 | 1.2525 | 1.5593 | -7.9963 | 3.0006 | 0.8901 |
| 47 | H | 0.9266 | 0.1628 | -3.0757 | -1.0207 | -2.53 | -6.7231 | -5.844 | 2.4389 | 1.1752 | -9.4275 | 2.1051 | 0.4069 |
| 48 | H | -2.9126 | 0.842 | -3.2071 | 1.8777 | -1.0616 | -4.5641 | -11.1497 | -0.6978 | 1.51 | -11.3045 | -0.0404 | -2.1271 |
| 49 | H | -1.3994 | 0.9511 | -4.0876 | 1.4503 | -2.2379 | -5.7931 | -10.4616 | 0.2261 | 2.8534 | -10.5964 | 1.5438 | -2.4558 |
| 50 | H | -1.4044 | 0.415 | -2.4016 | 0.2381 | -1.0892 | -5.2094 | -10.7215 | 1.007 | 1.284 | -10.7189 | 0.9382 | -0.7935 |

**Table S2.** ^1^H NMR data of **1**, **2**, **5**, and **6** in DMSO-*d*_6_ at 800 MHz.

| No. | **1** | **2** | **5** | **6** |
| --- | --- | --- | --- | --- |
|  | *δ*_H_, multi (*J* in Hz) | *δ*_H_, multi (*J* in Hz) | *δ*_H_, multi (*J* in Hz) | *δ*_H_, multi (*J* in Hz) |
| 1 | – | – | NH, 10.98 s | NH, 11.01 s |
| 6 | 7.52 s | 7.23 s | 7.01 s | 7.01 s |
| 7 | 2.46 t (9.9) | 2.53 dd (11, 3.8) | 2.11 t (10.1) | 2.45 dd (10.7, 3.8) |
| 8 | 1.52 m | 1.51 m | 1.41 m | 1.55 |
| 9 | 1.71 m  1.05 m | 1.78 m  1.26 m | 1.69 m  0.94 m | 1.77 m  1.24 m |
| 10 | 1.65 m | 1.60 m | 1.62 m | 1.60 m |
| 11 | 1.81 m  0.86 m | 1.63 m  0.83 m | 1.76 m  0.79 m | 1.62 m  0.81 m |
| 12 | 1.50 m | 1.63 m | 1.67 m | 1.62 m |
| 13 | 3.93 m | 4.66 m | 3.66 m | 4.55 m |
| 14 | 1.33 d (6.2) | 1.33 d (6.2) | 1.20 d (6.2) | 1.25 d (6.2) |
| 15 | 0.94 d (6.5) | 0.81 d (6.4) | 0.92 d (6.4) | 0.85 d (6.4) |
| 16 | 1.01 d (6.8) | 0.86 d (6.7) | 1.03 d (6.7) | 0.85 d (6.4) |
| 2'/6' | 7.30 (brd, 8.4) | 7.35 (brd, 8.7) | 7.17 brd (8.7) | 7.19 brd (8.4) |
| 3'/5' | 6.78 (brd, 8.4) | 6.71 (brd, 8.7) | 6.73 brd (8.7) | 6.73 brd (8.4) |
| 4'-OH | 9.48 brs | 9.34 brs | 9.39 s | 9.40 s |

**Table S3.** ^1^H and ^13^C NMR data of compound **4** and its isomers.

| No. | **Isomer 1 (with 10*S*)** | | **Isomer 2 (with 10*R*)** | | **4** | |
| --- | --- | --- | --- | --- | --- | --- |
|  | *δ*_C_ | *δ*_H_, multi (*J* in Hz) | *δ*_C_ | *δ*_H_, multi (*J* in Hz) | *δ*_C_ | *δ*_H_, multi (*J* in Hz) |
| 1 | - | - |  | - | – | – |
| 2 | 158.3 | - | 158.4 | - | 165.2 (C) | – |
| 3 | 114.7 | - | 114.8 | - | 111.6 (C) | – |
| 4 | 156.0 | - | 156.0 | - | 164.3 (C) | – |
| 5 | 115.2 | - | 115.3 | - | 117.7 (C) | – |
| 6 | 127.5 | 5.83 | 127.5 | 5.83 | 132.7 (CH) | 7.20 s |
| 7 | 123.6 | 5.67 | 123.2 | 5.68 | 117.0 (CH) | 5.77 s |
| 8 | 142.7 | - | 143.6 | - | 114.4 (C) | – |
| 9 | 51.7 | 1.97,1.68 | 52.3 | 2.02,1.54 | 48.3 (CH_2_) | 2.25 dd (13, 5.9)  2.00 dd (13, 8.3) |
| 10 | 33.8 | 1.33 | 34.6 | 1.27 | 32.8 (CH) | 1.75 m |
| 11 | 43.2 | 1.63,1.73 | 45.1 | 1.95,1.49 | 41.3 (CH_2_) | 2.10 m  1.85 m |
| 12 | 128.4 | 5.04 | 130.0 | 4.99 | 131.3 (CH) | 5.46 m |
| 13 | 133.4 | 5.46 | 133.1 | 5.65 | 127.3 (CH) | 5.46 m |
| 14 | 24.1 | 1.19 | 24.1 | 1.19 | 18.3 (CH_3_) | 1.64 d (4.1) |
| 15 | 28.0 | 0.66 | 28.5 | 0.58 | 20.3 (CH_3_) | 0.96 d (6.5) |
| 16 | 21.9 | 1.16 | 22.0 | 1.15 | 18.9 (CH_3_) | 1.59 s |
| 1' | 129.4 | - | 129.3 | - | 126.8 (C) | – |
| 2'/6' | 133.7 | 6.70 | 133.7 | 6.70 | 131.7 (CH) | 7.24 (brd, 8.4) |
| 3'/5' | 112.8 | 5.99 | 112.8 | 5.97 | 116.4 (CH) | 6.81 (brd, 8.4) |
| 4' | 152.2 | - | 152.2 |  | 158.3 (C) | – |
